# Chemical Proteomics Reveals Antibiotic Targets of Oxadiazolones in MRSA

**DOI:** 10.1021/jacs.2c10819

**Published:** 2022-12-30

**Authors:** Alexander T. Bakker, Ioli Kotsogianni, Liza Mirenda, Verena M. Straub, Mariana Avalos, Richard J. B. H. N. van den Berg, Bogdan I. Florea, Gilles P. van Wezel, Antonius P. A. Janssen, Nathaniel I. Martin, Mario van der Stelt

**Affiliations:** † Department of Molecular Physiology, Leiden Institute of Chemistry, Leiden University, Leiden 2300 RA, The Netherlands; ‡ Biological Chemistry Group, Institute of Biology Leiden, Leiden University, Leiden 2333 BE, The Netherlands; § Department of Molecular Biotechnology, Institute of Biology Leiden, Leiden University, Leiden 2333 BE, The Netherlands

## Abstract

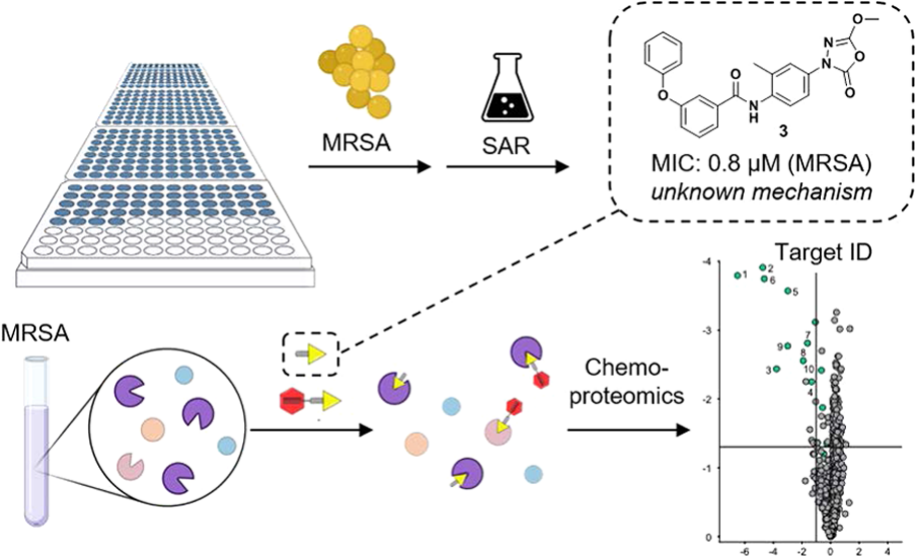

Phenotypic screening is a powerful approach to identify novel antibiotics, but elucidation of the targets responsible for the antimicrobial activity is often challenging in the case of compounds with a polypharmacological mode of action. Here, we show that activity-based protein profiling maps the target interaction landscape of a series of 1,3,4-oxadiazole-3-ones identified in a phenotypic screen to have high antibacterial potency against multidrug-resistant *Staphylococcus aureus*. In situ competitive and comparative chemical proteomics with a tailor-made activity-based probe, in combination with transposon and resistance studies, revealed several cysteine and serine hydrolases as relevant targets. Our data showcase oxadiazolones as a novel antibacterial chemotype with a polypharmacological mode of action, in which FabH, FphC, and AdhE play a central role.

## Introduction

The emergence of multidrug-resistant bacteria combined with a dearth of new antibiotics poses a serious threat to global health.^1−3^ Among Gram-positive pathogens, methicillin-resistant *Staphylococcus aureus* (MRSA) remains the most worrisome. Recent data indicate that in 2019, drug-resistant staphylococcal infections, due predominantly to MRSA, were associated with a staggering 750,000 deaths worldwide.^4^ New antibiotics with unprecedented modes of action (MoAs) are urgently required to counteract antimicrobial drug resistance.

While target-based screens are commonly applied to identify small molecule hits in traditional drug discovery projects, such strategies are less successful in antibiotic discovery.^5^ Phenotypic screening has instead emerged as a promising approach to identify antibiotics with novel MoAs.^6−8^ However, for hits found by phenotypic screening, target elucidation often presents a challenge. Recently, chemical proteomics has emerged as a powerful chemical biology technique to map target interaction landscapes of experimental drugs,^9−11^ including compounds with antibacterial activity.^12,13^ Inspired by these approaches, we set out to combine phenotypic screening with chemical proteomics to discover new anti-MRSA antibiotics and their interacting proteins.

## Results

**Figure 1:**
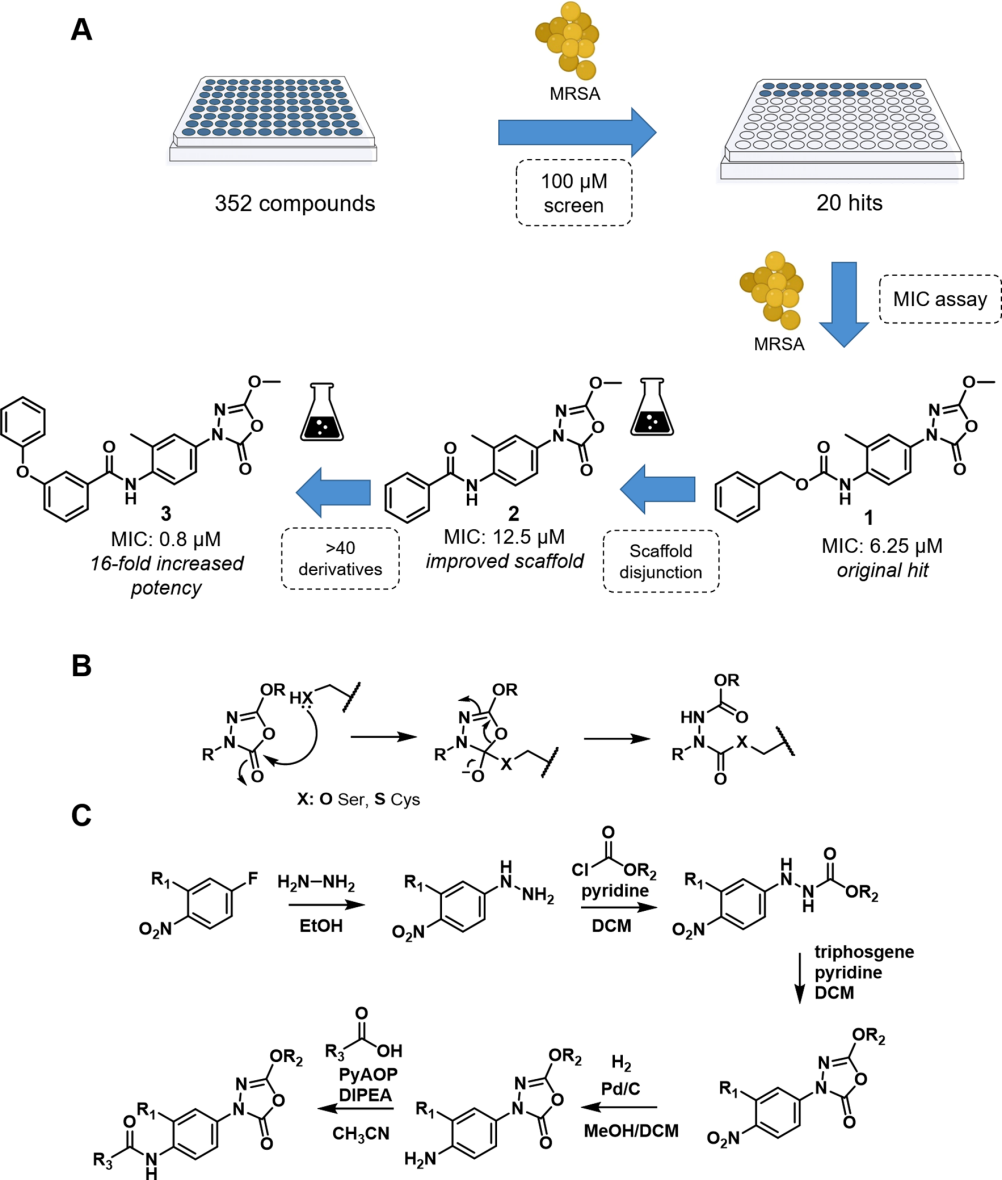
(A) Flowchart from the initial screen to lead compound **3**. (B) Proposed reaction mechanism of 1,3,4-oxadiazole-2-one derivatives toward reactive serine and cysteines. (C) General route for oxadiazolone synthesis. BioRender Premium with an academic license was used for Figure 1A.

To this end, a focused library of 352 small molecules derived from our in-house drug discovery programs was constructed. These compounds were selected based on structural diversity, drug-like properties, and their lack of previous antibiotic testing. This compound set was first screened at 100 μM for antibacterial activity against MRSA (Figure 1A). This revealed 25 compounds that prevented bacterial growth (Supporting Data 1). Subsequently, the minimum inhibitory concentration (MIC) was determined for each of the 25 hits. Benzyl (4-(5-methoxy-2-oxo-1,3,4-oxadiazol-3(2*H*)-yl)-2-methylphenyl)-carbamate **1** was most potent with an MIC of 6.25 μM (2.2 μg/mL). Notably, **1** contains an oxadiazolone moiety previously shown to covalently react with catalytically active serine and cysteine residues in enzymes (Figure 1B).^14−16^

To determine the structure–activity relationship and optimize the potency of **1**, 61 derivatives were synthesized and tested for anti-MRSA activity (Tables S1–S6 and Supporting Data 2). This led to the identification of **2** as a simplified scaffold with comparable antibacterial activity. Subsequent systematic modification of **2** (Figure 1C) resulted in the discovery of *N*-(4-(5-methoxy-2-oxo-1,3,4-oxadiazol-3(2*H*)-yl)-2-methylphenyl)-3-phenoxy-benzamide **3** as our lead compound with a 16-fold improvement in potency against both MRSA USA300 (MIC = 0.8 μM, 0.3 μg/mL) and *S. aureus* ATCC 29213 (MIC = 1.6 μM) compared to hit **1**.

**Figure 2:**
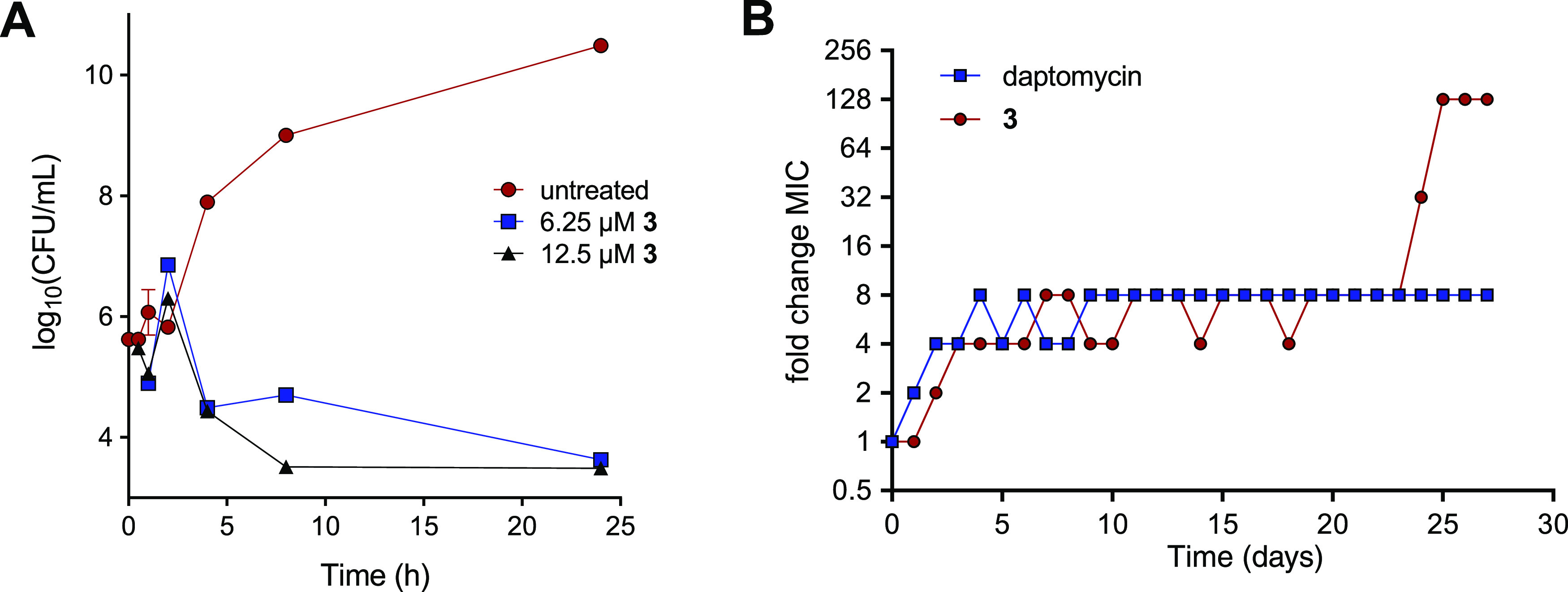
(A) Time-dependent killing by **3** of MRSA USA300. (B) Resistance development of MRSA USA300 against **3** and daptomycin during daily serial passaging with 0.25× MIC concentrations.

Extended screening of **3** revealed it to be highly active against a variety of clinical isolates, including vancomycin-resistant strains (Table 1 and Supporting Data 3). Time-kill experiments also showed that **3** killed 99% of bacteria within 24 h, starting from a 10^6^ CFU/mL inoculum (Figure 2A). Furthermore, **3** exhibits low cytotoxicity (Table S7: selectivity ratio HEK293T/MRSA USA300 = 10.5, HepG2/MRSA300 = 20.3) and is nonhemolytic (Figure S1).

**Table 1:** Minimum Inhibitory Concentration (MIC) of Compound **3**, and Clinically Relevant Antibiotics against a Bacterial Panel^a^

	MIC (μM)				
organism	strain	**3**	meropenem	vancomycin	daptomycin
*S. aureus*	MRSA USA300	0.8	1.1	1.4	1.2
	NY-155 (MRSA)	0.8	9.1	0.7	1.2
	MRSA131	1.6	2.3	0.7	1.2
	COL (MRSA)	1.6	293	1.4	2.4
		1.6	≤0.1	0.7	1.2
	SA MER (VISA)	1.6	0.3	2.8	4.9
	LIM3 (VISA)	0.8	≤2.3	2.8	2.4
	NRS126 (VISA)	3.1	293	2.8	2.4
	BR-VRSA	1.6	>293	88	1.2
	VRSA-1	3.1	293	88	1.2
	VRSA-2	0.8	293	88	≤0.6
*Enterococcus faecium*		>50	≤0.1	0.7	1.2
Gram-negative		>50	≤0.1–2.3	>88	>80

a MSSA: methicillin-susceptible *S. aureus*, VISA: vancomycin-intermediate *S. aureus*, and VRSA: vancomycin-resistant *S. aureus*.

Next, we set out to generate strains resistant to **3** to investigate both the rate and mechanism of resistance development. The serial passage of MRSA USA300 in sub-MIC concentrations of **3** yielded resistant mutants after 4 weeks (Figures 2B and S2). In comparison, resistance to daptomycin, a clinically used lipopeptide antibiotic, emerged more slowly and did not exceed 8× MIC, a finding in line with previous reports.^17,18^ Notably, resistance toward **3** initially increased and then stabilized for several weeks before progressing to significantly higher values. This may indicate that multiple mutations are required to fully induce resistance, suggesting a polypharmacological MoA. Also of note, the passaged strains exhibiting resistance to **3** did not show cross-resistance to other antibiotic classes (Table S8).

**Figure 3:**
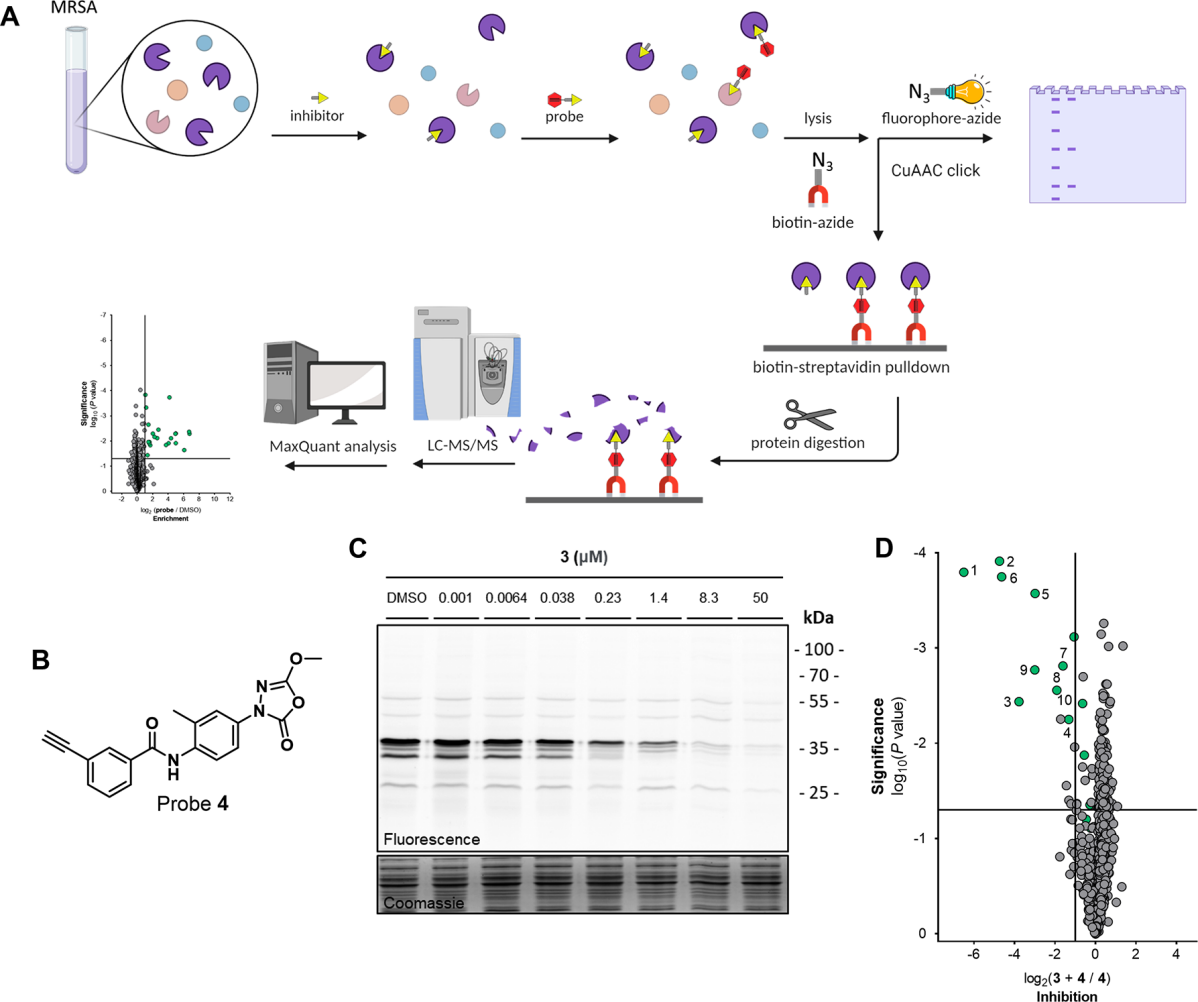
(A) In situ competitive ABPP workflow on MRSA with using either sodium dodecyl sulfate-polyacrylamide gel electrophoresis (SDS-PAGE) or mass spectrometry read-out. (B) Activity-based probe **4**. (C) Gel-based competitive ABPP over a concentration range of **3** versus 1 μM probe **4**. (D) MS data inhibition plot comparing the labeled proteome of samples preincubated with inhibitor **3** (10 μM) followed by probe **4** labeling (3 μM) to solely probe-labeled samples. The vertical and horizontal threshold lines represent a log_2_ change of −1 and a log_10_(*P* value) of −1.3 (two-sided two-sample *t*-test, *n* = 3 independent experiments per group), respectively. Green dots indicate proteins that are probe targets, as defined in Table S9. BioRender Premium with an academic license was used for (A).

Having established the potent anti-*S. aureus* activity of the oxadiazoloness, we set out to identify interaction partners using activity-based protein profiling (ABPP). Given the covalent mechanism ascribed to the oxadiazolones, we hypothesized that a strategically positioned ligation handle on **3** could be used to introduce a fluorescent or affinity tag (e.g., biotin) to visualize its interactions with proteins in living systems (Figure 3A). To this end, the meta-phenoxy group of **3** was substituted with an alkyne, resulting in activity-based probe **4** (Figure 3B). The antibacterial activity of **4** was confirmed in MRSA (MIC = 3.1 μM). The probe was subsequently used in an in situ competitive ABPP workflow (Figure 3A).^19^ To do so, MRSA cells at the exponential phase were treated with competitor **3** or dimethyl sulfoxide (DMSO), followed by labeling with probe **4**. Bacteria were lysed and the probe-labeled proteins conjugated to a Cy5 fluorophore-azide via copper-catalyzed azide–alkyne click chemistry. This resulted in the clear labeling of several proteins by **4**, of which most were dose-dependently outcompeted by **3** (Figure 3C). To identify the probe-labeled proteins, a biotin-azide reporter was also employed, allowing for affinity enrichment and identification of probe-labeled proteins by mass spectrometry (MS)-based proteomics.^20^ Around 30 proteins were found to be significantly enriched (*P* < 0.05, >2-fold enrichment) by probe treatment (Figure S3, Table S9, and Supporting Data 4A). Pretreatment with **3** significantly inhibited (*P* < 0.05, >2-fold inhibition) the labeling of 10 proteins by probe **4** (Figure 3D), suggesting that these proteins are interaction partners of oxadiazolone **3**.

Among the MRSA proteins thus identified, the Fph proteins (B, C, E, H) were recently annotated as fluorophosphonate-binding hydrolases.^21^ FphB was found to be a fatty acid metabolizing virulence factor, while FphE activity has been used to phenotypically characterize MRSA.^22^ HZ1 is reported to have hydrolase activity (Table 2), while IB7 has putative thiolase activity,^23^ and FI2 is an uncharacterized protein. HH9 has recently been annotated as a lipase of negatively charged fatty acids.^24^ FabH is essential for bacterial fatty acid synthesis and has been explored as a drug target,^26−28^ while AdhE is an aldehyde–alcohol dehydrogenase, essential in facultative anaerobic organisms in anaerobic conditions.^29,30^ Both FabH and AdhE are known to metabolize substrates using an active site cysteine.

**Table 2:** List of Probe Targets Significantly Outcompeted by **3**

#	uniprot ID	protein	description	sequence length (aa)	gene	essentiality	references
1	Q2FDS6	FphE	uncharacterized hydrolase	276	SAUSA300_2518	no	(21) and (22)
2	Q2FI93	FabH	3-oxoacyl-[acyl-carrier-protein] synthase 3	313	*fabH*	yes	(25−28)
3	A0A0H2XJL0	FphH	carboxylesterase	246	*est*	no	(21)
4	A0A0H2XHZ1	HZ1	putative lysophospholipase	271	SAUSA300_0070	no	
5	A0A0H2XHD0	FphC	hydrolase, α/β hydrolase fold family	304	SAUSA300_1194	no	(21)
6	A0A0H2XHH9	HH9	*S. aureus* lipase 3	347	SAUSA300_0641	no	(24)
7	A0A0H2XJG5	FphB	uncharacterized protein	322	SAUSA300_2473	no	(21)
8	A0A0H2XFI2	FI2	uncharacterized protein	275	SAUSA300_0321	no	
9	A0A0H2XIB7	IB7	acetyl-CoA c-acetyltransferase	379	*vraB*	no	(23)
10	A0A0H2XG10	AdhE	aldehyde–alcohol dehydrogenase	869	*adhE*	no	(29−31)

**Figure 4:**
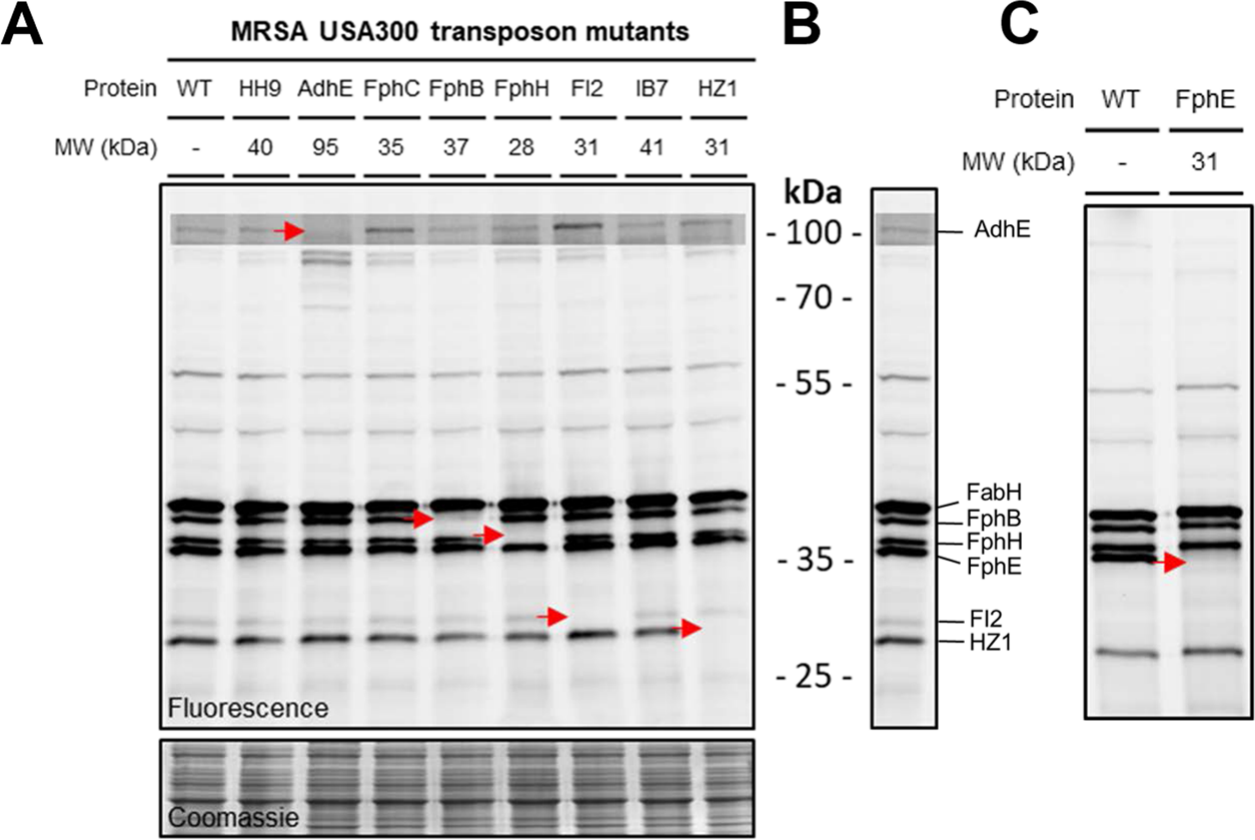
(A) Labeling of MRSA USA300 transposon mutants with **4**. All mutants shown except FphE. (B) Wild type (WT) MRSA USA300 treated with probe **4**. Bands annotated with corresponding proteins. (C) Labeling of the FphE transposon mutant.

We next screened the probe-labeled proteome of nine transposon mutants of MRSA that lack the gene encoding one of the identified target proteins of **3** (Figure 4A,C). The labeling of AdhE, FphB, FphH, FI2, HZ1, and FphE, but not FphC and HH9, could be attributed to specific fluorescent bands on SDS-PAGE (Figure 4B). The lower resolution of gel-based ABPP or insufficient sensitivity compared to MS-based ABPP may explain why FphC and HH9 were not identified on the gel. Since FabH is essential for MRSA viability, no transposon mutant is available for this protein. Instead, we confirmed the identity of FabH on the gel by competitive ABPP using the selective FabH inhibitor Oxa2 (Figure S4).

**Figure 5:**
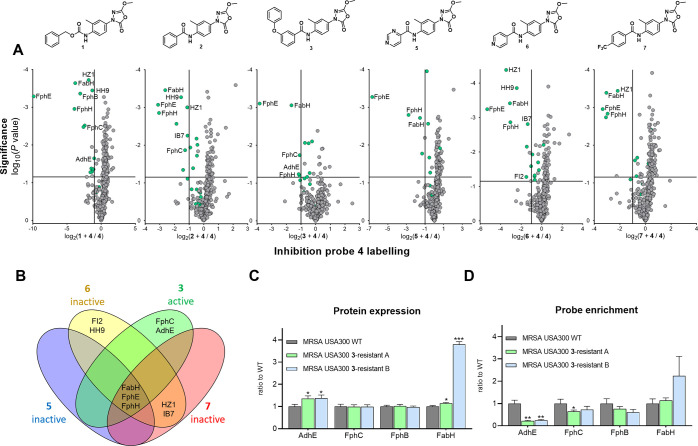
(A) Individual inhibition plots of active compounds **1**, **2**, and **3** and inactive compounds **5**, **6**, and **7**. **3** was dosed at 1 μM, while **1**, **2**, and the inactive analogues were dosed at 10 μM. The vertical and horizontal threshold lines represent a log_2_ change of −1 and a log_10_(*P* value) of −1.3 (two-sided two-sample *t*-test, *n* = 3 independent experiments per group), respectively. Green dots indicate proteins that are probe targets, as defined in Table S9. (B) Venn diagram showing the overlap of >50% inhibited proteins between **3** and inactive analogues **5**–**7**. (C) Relative general protein levels in strains resistant to **3** compared to WT. Each group was compared to WT using a two-sided two-sample *t*-test, *n* = 3 independent experiments per group. Statistical significance: ***P < 0.001; **P < 0.01; *P < 0.05; n.s. if *P* > 0.05. (D) Relative protein levels enriched by **4** in strains resistant to **3** (Figure 2B) compared to wild type. Each group was compared to WT using a two-sided two-sample *t*-test, *n* = 3 independent experiments per group.

To assess which target proteins were associated with the antibiotic effect, we hypothesized that the protein labeling profile of potent oxadiazolones (MIC ≤ 12.5 μM) would be different than that of analogues with weak activity (MIC > 50 μM). We, therefore, compared the interaction profile of three inactive derivatives (**5**–**7**) with that of three active compounds (**1**–**3**) in a competitive chemical proteomics assay (Figures 5A and S5 and Supporting Data 4B). Strong FphB labeling was observed upon treatment with **1**, but not by the other compounds. F12, IB7, HH9, and HZ1 were not significantly labeled by bioactive oxadiazolone **3** but did show engagement with inactive compounds **5**, **6**, or **7**. FphE and FphH were strongly labeled by all compounds at 10 μM. These observations, in combination with the viability of the transposon mutants, suggest that specific inhibition of FphB, IB7, HH9, F12, FphE, FphH, or HZ1 is not responsible for the antimicrobial activity of **3**. FabH was significantly engaged, but not fully, by all compounds. Since the transposon mutant of FabH is not viable, and because Oxa2, a specific FabH inhibitor, can inhibit bacterial growth, this implies that partial inhibition of FabH activity could contribute to the bioactivity of the oxadiazolones. For both FphC and AdhE, however, labeling was observed with one or more of the active compounds and not by the inactive analogues (Figures 5B and S6 and Table S10).

Thus, our chemical proteomics data reveal that multiple targets (in particular, FphC, AdhE, and FabH) play a role in the observed antimicrobial activity of the oxadiazolones. We also assessed the sensitivity of the AdhE and FphC transposon mutants to inactive analogues **5**–**7**. This revealed that both **6** and **7** showed increased antimicrobial activity in both transposon mutants (Tables 3 and S11), but not in a FphB transposon mutant (included as a negative control). Compound **5**, which does not inhibit AdhE and very weakly inhibits FphC labeling (<20%), remained inactive against all transposon mutants. No direct synergy of FabH inhibition with any other single target could be concluded, as the antibacterial activity of Oxa2 was not potentiated on any of the transposon mutants (Table S11). While one cannot exclude the role of additional proteins, we interpret these data to mean that the combined engagement of FabH, FphC, AdhE, and, to some extent, FphE, is responsible for the antimicrobial activity observed for the oxadiazolones.

**Table 3:** MIC Values of MRSA USA300 Transposon Mutants of Target Proteins

MIC (μM)			
	inactive **5**	inactive **6**	inactive **7**
MRSA USA300 WT	>50	>50	>50
AdhE transposon mutant	>50	**25**	**12.5**
FphC transposon mutant	>50	**50**	**25**
FphB transposon mutant	>50	>50	>50

To test this hypothesis, we investigated whether the oxadiazolone targets were changed in two MRSA strains passaged to become resistant to **3** compared to WT MRSA. Using chemical (Figures 5C and S7 and Supporting Data 4C,D) and global proteomics (Figures 5D and S8 and Supporting Data 5), it was observed that AdhE and FphE engagement by probe **4** was significantly decreased, while protein abundance was upregulated in the two resistant strains. FphC engagement was also reduced, but to a lower extent. Interestingly, FabH protein levels were significantly increased in the resistant strains, which was accompanied by cross-resistance of these strains to FabH inhibitor Oxa2 (32× increase in MIC, Table S8). Taken together, these data suggest that the combined inhibition of FabH, FphC, FphE, and AdhE contributes to the antimicrobial activity of compound **3**.

The two passaged MRSA strains exhibiting resistance to **3** were also screened for mutations in the genes that encode for the 10 target proteins (Supporting Data 6). This revealed a single point mutation in one of the resistant strains corresponding to Thr146Ile in the FabH protein. Of note, this is also the strain in which highly upregulated FabH levels were observed. Given the lack of other mutations in the strains resistant to **3**, we concluded that genetic changes are not responsible for the high resistance developed and that the observed resistance must come from other mechanisms. To assess the stability of this resistance phenotype, the resistant strains were cultured in the absence of compound **3** for several days and then tested for susceptibility (Figure S9). Within a few days, the MIC dropped from 128× MIC to 8× MIC. This suggests that the observed resistance to **3** is inducible upon exposure to the compound and is lost in the absence of the compound.

## Conclusions

To summarize, we here disclose oxadiazolones as a new chemotype with antibiotic activity against *S. aureus* strains including drug-resistant isolates. A medicinal chemistry program combined with chemical proteomics led to the identification of potent antibacterial compound **3** capable of interacting with multiple bacterial cysteine and serine hydrolases in a covalent manner. Three complementary lines of investigation point to FabH, FphC, AdhE, and to some extent FphE playing central roles in the antimicrobial activity of the oxadiazolones: (i) comparative chemical proteomics, (ii) gain of function in transposon mutants, and (iii) resistance-induced proteomic changes. FabH has previously been identified as a drug target, whereas the functions of AdhE and FphC have been less explored. Recent studies implicate AdhE as a virulence factor in *Escherichia coli*,^31^ while FphC is a predicted membrane-bound serine hydrolase of unknown function. We also cannot rule out that other factors, not detected by our chemical proteomics approach, might contribute to the antibacterial effect of **3**. Interestingly, the resistance developed to **3** was transient, as it quickly diminished upon removal of the compound, and genomic screening of target proteins in the resistant strains identified only a single FabH mutation. This evidence suggests that mutations in key targets are not responsible for resistance to **3**. Notably, this reversibility of resistance may be advantageous for the potential application of such compounds as antibiotics.

To conclude, our findings further highlight the value of synthetic compound libraries as an excellent source for antibiotic drug discovery complementary to natural products. By applying comparative and competitive chemical proteomics, using a new tailor-made activity-based probe with a strategically positioned ligation tag, we successfully highlighted the polypharmacological mode of action of the oxadiazolones and identified their targets in MRSA. Notably, a target-based approach alone would not have been able to uncover the mode of action of the oxadiazolones, thereby showcasing the power of chemical proteomics as a valuable chemical biology technique for antibiotic drug discovery. Future experiments are directed toward understanding the biological role of these targets, the nature of the resistance, and further optimization of the compounds as viable drug candidates.

## Supporting Information Available

SAR, cytotoxicity data, MIC values of selected compounds against resistant strains and transposon mutants, and list of enriched proteins (Tables S1–S11); additional data of the chemical proteomics, toxicity, and resistance experiments; and additional experimental details, materials, and methods, including synthetic procedures and NMR data of key compounds (Figures S1–S9) (PDB)
Supporting Data 1–6 (XLSX)
