# Supplementary material for: Chemical Proteomics Reveals Antibiotic Targets of Oxadiazolones in MRSA
Source: J Am Chem Soc. 2022 Dec 30;145(2):1136–43. doi: 10.1021/jacs.2c10819 (PMC9853856; doi:10.1021/jacs.2c10819)
Supplement: Supplementary file 1 — ja2c10819_si_001.pdf [file ja2c10819_si_001.pdf]

Supplementary information

Chemical Proteomics Reveals Antibiotic Targets of Oxadiazolones in MRSA

Alexander T. Bakker,‡,† Ioli Kotsogianni,‡,§ Liza Mirenda,† Verena M. Straub,† Mariana Avalos,⊥ Richard J. B. H. N. van den Berg,† Bogdan I. Florea,† Gilles P. van Wezel,⊥ Antonius P. A. Janssen,† Nathaniel I. Martin,*,§ Mario van der Stelt*,†

† Department of Molecular Physiology, Leiden Institute of Chemistry, Leiden University & Oncode Institute, Leiden 2300 RA, The Netherlands

§ Biological Chemistry Group, Institute of Biology Leiden, Leiden University; Leiden 2333 BE, The Netherlands

⊥ Department of Molecular Biotechnology, Institute of Biology Leiden, Leiden University; Leiden 2333 BE, The Netherlands

*e-mail: n.i.martin@biology.leidenuniv.nl; m.van.der.stelt@chem.leidenuniv.nl

‡ These authors contributed equally to this work.

Table of contents

Supplementary Tables 3
Supplementary Figures 13
Supplementary Data. 17
Materials and Methods 18
Reagents & materials 18
Bacterial strains 18
Hemolytic activity 18
Library screen 19
Minimum inhibitory concentration (MIC) 19
Minimum bactericidal concentration (MBC) 19
Time-kill assay 20
Resistance induction assay 20
Mammalian cell culture 20
Cytotoxicity assay (MTT) 21
Sample preparation ABPP 21
Gel-based ABPP and in-gel fluorescence analysis 22
Preparation for LC-MS based ABPP 22
Preparation for full proteome analysis 23
LC-MS measurement and analysis 23
MS data analysis and processing 25
Synthetic procedures 26
General remarks 26
NMR spectra data key compounds 73
References 80

Supplementary Tables

SAR tables

Table S1. Dissecting structure **1** for antibacterial activity.

ID	Structure	MIC (µM)		ID	Structure	MIC (µM)	
		MRSA	SA			MRSA	SA
**1**	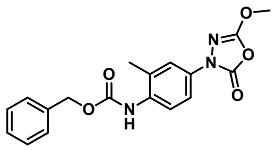	6.25	12.5	**13**	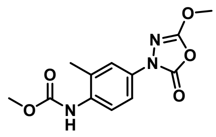	50	>50
**8**	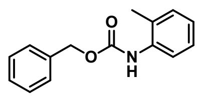	>50	>50	**14**	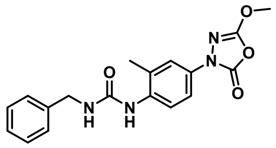	>50	>50
**9**	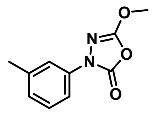	>50	>50	**15**	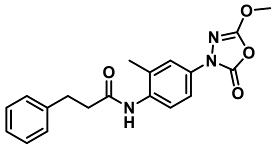	25	>50
**10**	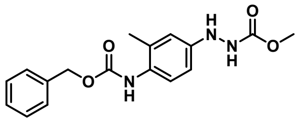	>50	>50	**2**	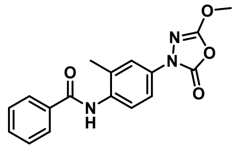	12.5	25
**11**	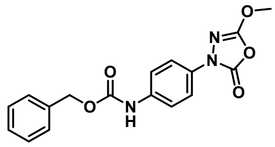	25	>50	**16**	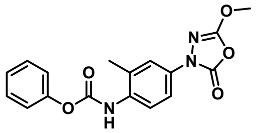	>50	>50
**12**	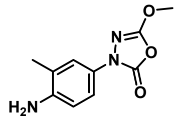	>50	>50	**17**	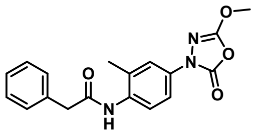	12.5	50

In this and subsequent tables: MRSA = MRSA USA300, SA = *S. aureus* ATCC 29213

Table S2. Middle ring derivatives.

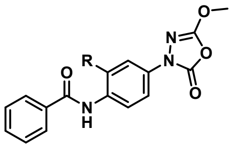

ID	R	MIC (μM)	
		MRSA	SA
**2**	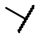	12.5	25
**18**	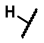	>50	>50
**19**	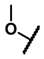	25	>50
**20**	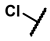	25	50
**21**	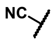	25	>50

Table S3. Left ring variations of compound **2**.

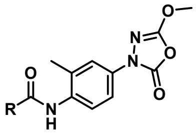

ID	R	MIC (µM) MRSA	MIC (µM) SA	ID	R	MIC (µM) MRSA	MIC (µM) SA
**2**	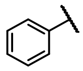	12.5	25	**31**	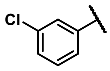	6.25	>50
**6**	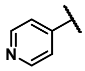	>50	>50	**32**	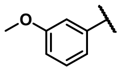	6.25	12.5
**22**	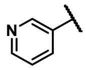	50	>50	**33**	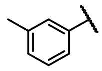	12.5	>50
**23**	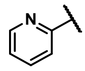	>50	>50	**34**	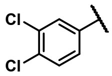	>50	>50
**24**	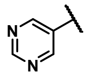	>50	>50	**35**	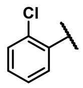	12.5	25
**5**	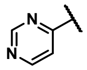	>50	>50	**36**	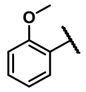	>50	>50
**25**	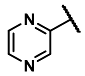	>50	>50	**37**	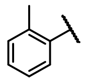	25	>50
**26**	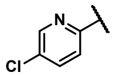	6.25	50	**4**	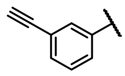	3.13	25
**27**	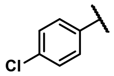	6.25	>50	**38**	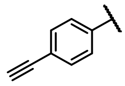	25	>50
**28**	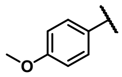	12.5	>50	**3**	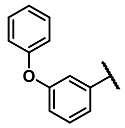	0.8	1.6
**29**	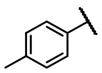	6.25	25 - 50	**39**	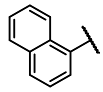	25	50
**30**	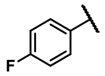	6.25	25	**40**	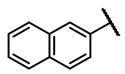	1.6	>50
**7**	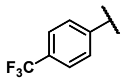	>50	>50				

Table S4. Variations of compound **3**.

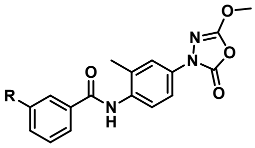

ID	R	MIC (μM)		ID	R	MIC (μM)	
		MRSA	SA			MRSA	SA
**3**	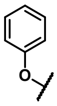	0.8	1.6	**48**	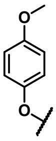	1.6	3.1
**41**	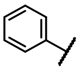	>12.5	>50	**49**	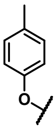	3.1	1.6
**42**	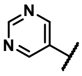	12.5	>50	**50**	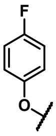	1.6	3.1
**43**	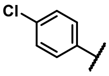	>12.5	>50	**51**	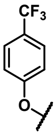	3.1	>12.5
**44**	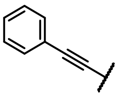	>12.5	>50	**52**	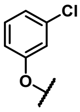	0.8 – 1.6	3.1 – 6.25
**45**	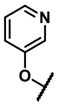	>12.5	>50	**53**	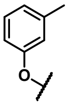	0.8	1.6 – 3.1
**46**	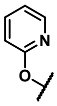	12.5	>50	**54**	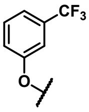	1.6	>12.5
**47**	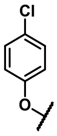	1.56	3.13				

Table S5. Warhead variations of compound **3**.

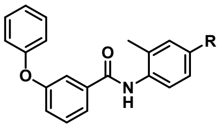

ID	R	MIC (μM)	
		**MRSA**	**SA**
**3**	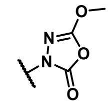	0.8	1.6
**55**	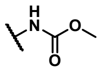	>12.5	>12.5
**56**	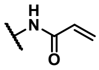	>12.5	>12.5
**57**	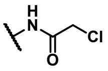	>12.5	>12.5
**58**	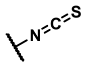	>12.5	>12.5

Table S6. Oxadiazolone side chain variations of compound **3**.

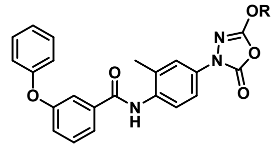

ID	R	MIC (μM)	
		MRSA	SA
**3**	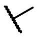	0.8	1.6
**59**	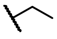	0.8	3.1
**60**	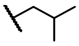	>12.5	>12.5
**61**	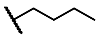	1.6	>12.5
**62**	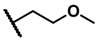	3.1	>12.5

Cytotoxicity data

Table S7. Human cytotoxicity testing on selected derivatives.

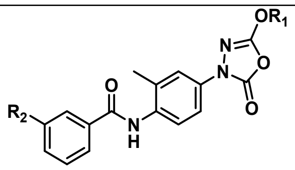

ID	R1	R2	IC50 (μM)		MIC (μM)		Selectivity ratio (HEK293/MRSA)	Selectivity ratio (HepG2/MRSA)
			HEK293T	HepG2	MRSA	SA		
**1**	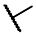	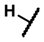	46	>50	12.5	25	3.7	>4.0
**3**	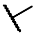	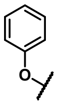	8.4	16.2	0.8	1.6	10.5	20.3
**4**	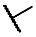	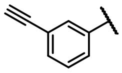	10.2	>50	3.1	25	3.3	>16.1
**50**	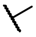	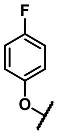	8.2	25	1.6	3.1	5.1	15.6
**52**	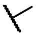	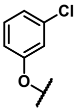	4.4	11	0.8 – 1.6	3.1 – 6.25	2.7 - 5.5	6.9 - 13.8
**53**	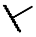	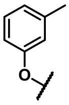	3.9	6.3	0.8	1.6 – 3.1	4.9	7.9
**54**	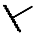	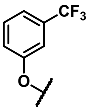	15	11	1.6	>12.5	9.4	6.9
**59**	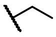	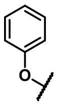	9.4	7.2	0.8	3.1	11.8	9.0
**60**	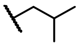	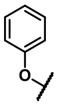	>50	>50	>12.5	>12.5	-	-
**61**	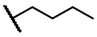	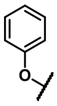	16	>50	1.6	>12.5	10.0	>31.3
**62**	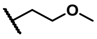	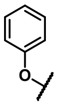	4.6	7.0	3.1	>12.5	1.5	2.3

Table S8. Minimum inhibitory concentrations of selected compounds against the **3**-resistant strains A/B obtained by serial passaging.

	**MIC** (µM)		
	USA300	**3**-resistant A	**3**-resistant B
Compound **3**	0.8	>50	>50
Probe **4**	3.1	>50	>50
Meropenem	2.3	2.3	2.3
Vancomycin	0.7	0.7	0.7
Daptomycin	1.2	1.2	1.2
Rifampicin	0.015	0.015	0.015
Novobiocin	0.13	0.13	0.13
Chloramphenicol	12.5	12.5	12.5
Ciprofloxacin	>100	>100	>100
Oxa2	0.4	12.5	12.5

Table S9. Significantly enriched proteins ($P < 0.05$, > 2-fold enrichment) in MRSA USA300 by probe **4** in comparison to DMSO-treated samples, as calculated by a two-sided two-sample t-test. These proteins are denoted by green dots in Figures 3D and S3. See Supplementary Data 4 for full table.

Protein IDs	Protein names	Protein description	Gene names	4 / DMSO	*P*-value
**Q2FDS6**	FphE	Uncharacterized hydrolase	SAUSA300_2518	108.24	0.000159
**Q2FI93**	FabH	3-oxoacyl-[acyl-carrier-protein] synthase 3	*fabH*	104.51	0.000109
**A0A0H2XJL0**	FphH	Carboxylesterase	*est*	67.42	0.002892
**A0A0H2XHZ1**	HZ1	Putative lysophospholipase	SAUSA300_0070	31.72	0.000860
**A0A0H2XHD0**	FphC	Hydrolase, alpha/beta hydrolase fold family	SAUSA300_1194	31.70	0.000178
**A0A0H2XHH9**	HH9	S. aureus lipase 3	SAUSA300_0641	30.60	0.000158
**A0A0H2XJG5**	FphB	Uncharacterized protein	SAUSA300_2473	20.71	0.000359
**A0A0H2XFI2**	FI2	Uncharacterized protein	SAUSA300_0321	16.51	0.001140
**A0A0H2XIB7**	IB7	Acetyl-CoA c-acetyltransferase	*vraB*	13.18	0.001232
**A0A0H2XGQ4**	A0A0H2XGQ4	Hydrolase, CocE/NonD family	SAUSA300_2531	6.67	0.000091
**Q2FK94**	ALDA	Putative aldehyde dehydrogenase AldA	*aldA*	5.99	0.000364
**A0A0H2XHF0**	FphA	Carboxylic ester hydrolase	*pnbA*	4.68	0.001104
**A0A0H2XHE0**	A0A0H2XHE0	Uncharacterized protein	SAUSA300_2093	4.06	0.000489
**A0A0H2XFJ9**	A0A0H2XFJ9	Peptidase, M20/M25/M40 family	SAUSA300_1460	3.21	0.001625
**A0A0H2XJF4**	A0A0H2XJF4	3-oxoacyl-[acyl-carrier-protein] synthase 2	*fabF*	2.99	0.001652
**Q2FF06**	ALDH	Putative aldehyde dehydrogenase	SAUSA300_2076	2.93	0.001234
**A0A0H2XI47**	FphI	Uncharacterized protein	SAUSA300_0430	2.73	0.000745
**A0A0H2XFW4**	A0A0H2XFW4	Peptidase, U32 family	SAUSA300_1569	2.73	0.002275
**A0A0H2XFN6**	FphF	Tributyrin esterase	*estA*	2.51	0.012576
**Q2FK11**	SCDA	Iron-sulfur cluster repair protein ScdA	*scdA*	2.23	0.000328
**A0A0H2XG10**	AdhE	Aldehyde-alcohol dehydrogenase	*adhE*	2.08	0.000095

Table S10. Loadings protein inhibition PC2.

Protein inhibiton	PC2 contribution (%)	direction
**AdhE**	**34**	**+**
**FphC**	**23**	**+**
IB7	16	-
FphB	11	+
FphH	6	-
F12	5	-
HZ1	5	-
HH9	0	-
FabH	0	-
FphE	0	-

Table S11. Antibacterial activity compounds **1**-**3**, **5**-**7** against transposon mutants.

			MIC (µM)						
NTML strain	Locus	Transposon	**1**	**2**	**3**	**5**	**6**	**7**	Oxa2
		WT	6.25	12.5	0.8	>200	>200	>50	0.4
NE114	151	AdhE	6.25	12.5	0.8	>200	**25**	**12.5**	0.4
NE204	1194	FphC	6.25	12.5	0.8	>200	50	**25**	0.4
NE532	2473	FphB	6.25	12.5	0.8	>200	100	>50	0.4
NE104	641	HH9	6.25	12.5	0.8 / 1.6	>200	50	50	0.4
NE1534	70	HZ1	6.25	12.5	0.8	>200	100	>50	0.8
NE1227	560	IB7	6.25	25	0.4 / 0.8	>200	50	>50	0.8
NE1122	763	FphH	6.25	25	0.8	>200	100	>50	0.8
NE1187	321	FI2	6.25	25	0.8	>200	100	>50	0.4
NE1779	2518	FphE	6.25	6.25	0.8	>200	50	**12.5**	0.4

Supplementary Figures

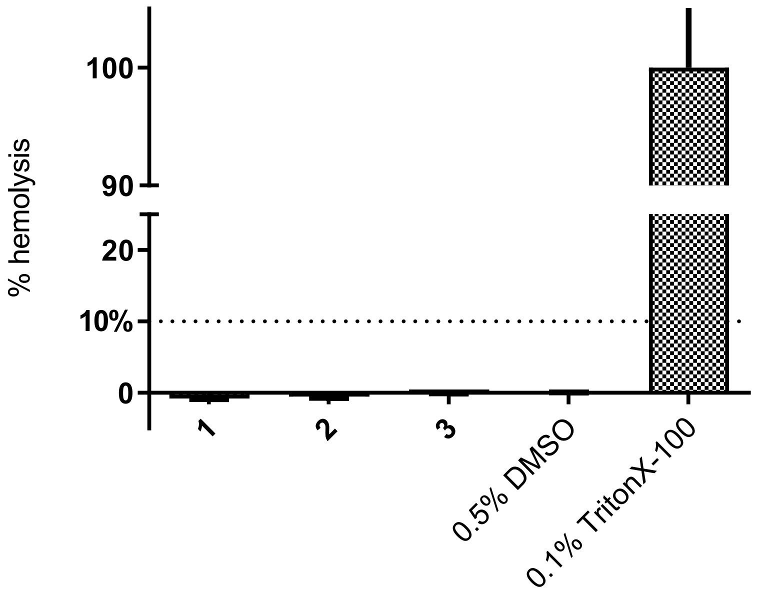

Figure S1. Normalized hemolytic activity of oxadiazolone compounds **1**-**3** at 50 µM after 20 h incubation time.

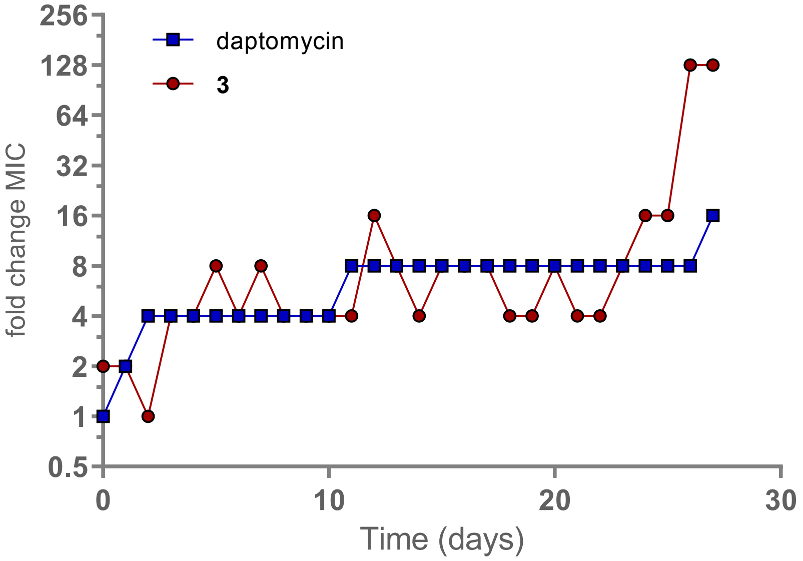

Figure S2. Resistance development of MRSA USA300 against **3** and daptomycin during daily serial passaging with 0.25× MIC concentrations. Second biological replicate (**3**-resistant B isolated at day 28).

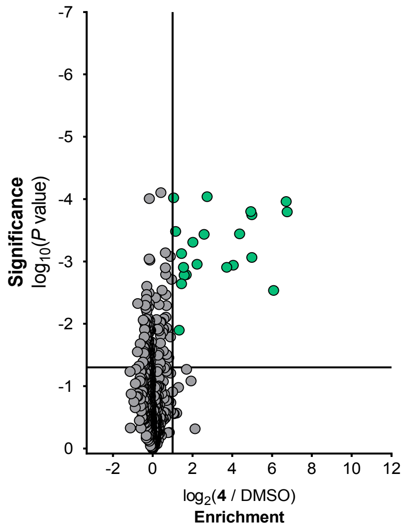

Figure S3. Mass spectrometry data enrichment plot comparing labelled proteome of 3 µM **4**-treated MRSA to DMSO-treated MRSA. The vertical and horizontal threshold lines represent a $\log_2$ change of 1 and a $\log_{10}(P$ value) of -1.3 (two-sided two-sample t-test, n=3 independent experiments per group), respectively. Green dots indicate proteins which are probe targets, as defined in Table S9. Green dots indicate proteins which are probe targets (>2-fold enriched, $P < 0.05$). Probe targets annotated in Table S9.

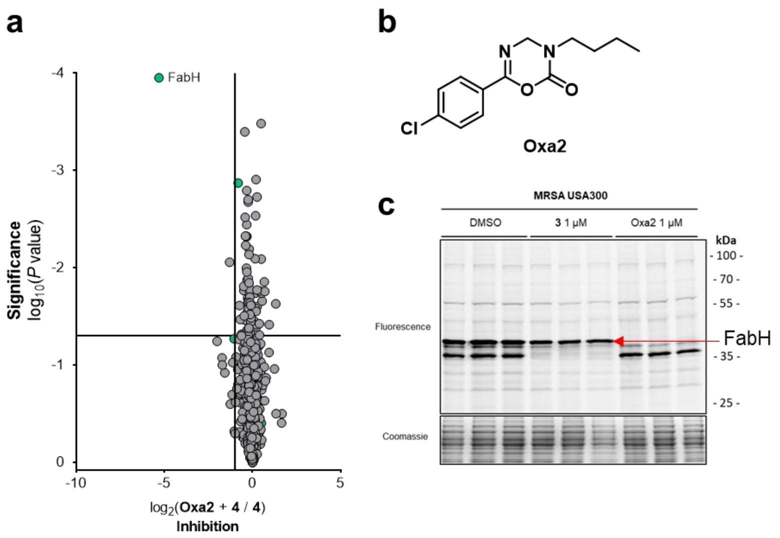

Figure S4. Chemical proteomics data Oxa2 reveals FabH band on SDS-PAGE. (a) Mass spectrometry data inhibition plot comparing labelled proteome of samples preincubated with Oxa2 (1 µM) followed by probe-labelling (3 µM) to solely probe-labelled samples. The vertical and horizontal threshold lines represent a $\log_2$ change of −1 and a $\log_{10}(P$ value) of -1.3 (two-sided two-sample t-test, n=3 independent experiments per group), respectively. (b) Structure Oxa2. (c) Gel-based competitive ABPP of indicated inhibitors followed by probe **4** (3 µM) incubation.

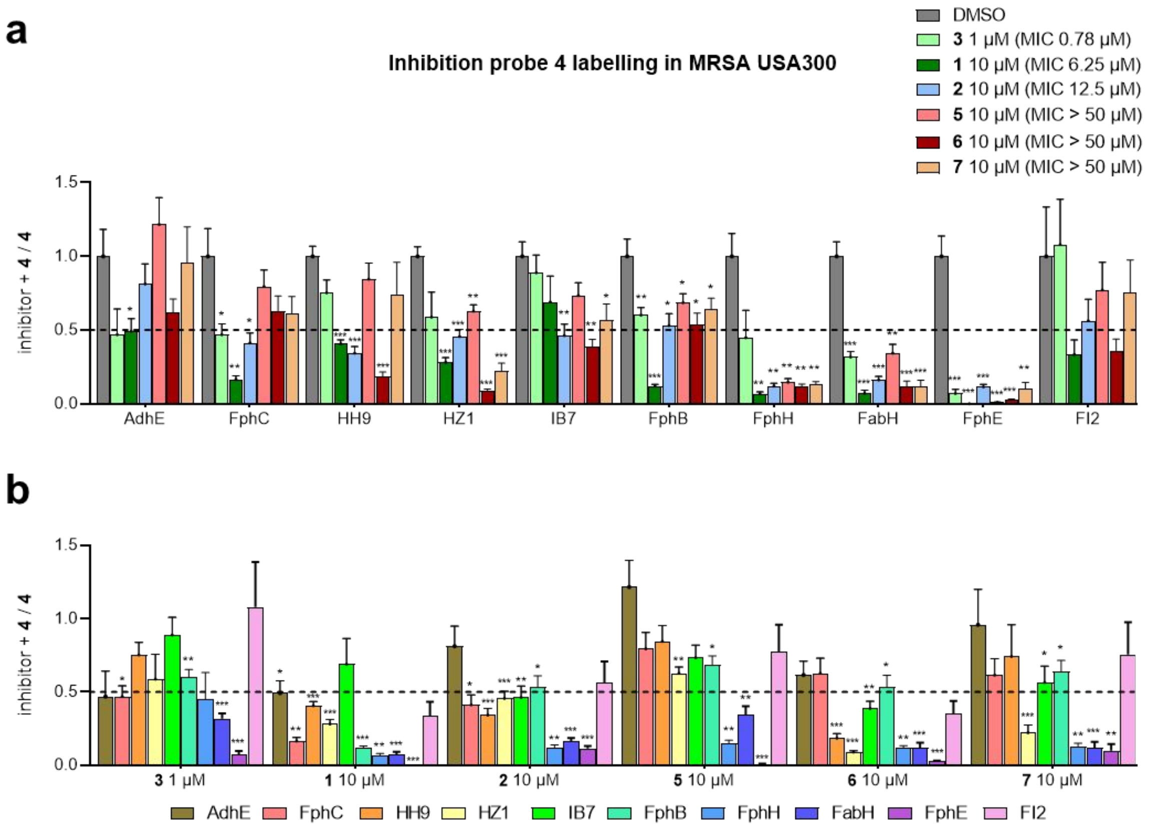

Figure S5. Bar graphs key protein inhibition in inactive ABPP experiment. Selected protein data found in Supplementary Data 4B plotted in GraphPad Prism 9.0.0. All groups consist of n=3 independent replicates. Statistical data was obtained by two-sided two-sample t-test, where every inhibitor-treated condition was tested against DMSO-treated control. Statistical significance: *** P <0.001; ** P < 0.01; * P < 0.05; n.s. if P > 0.05. (a) Bar graph inhibition profile per protein. (b) Bar graph inhibition profile per inhibitor.

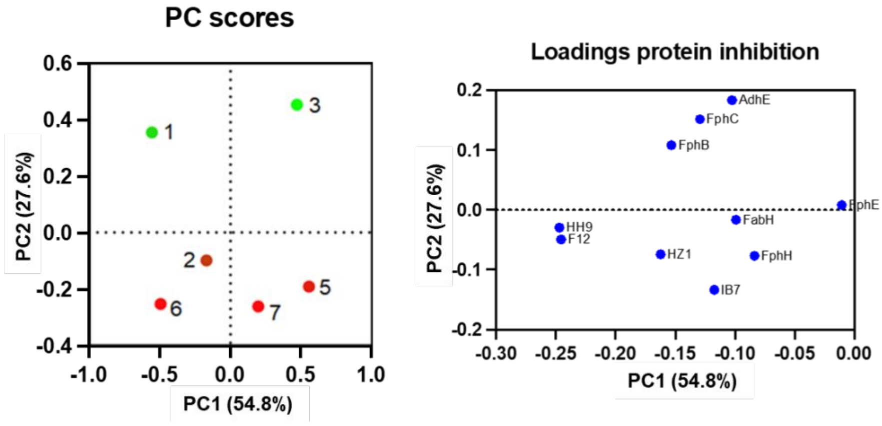

Figure S6. PCA analysis of the inhibition profiles of the six compounds. (left) Contribution of individual protein inhibition levels to PC1 and PC2 (right).

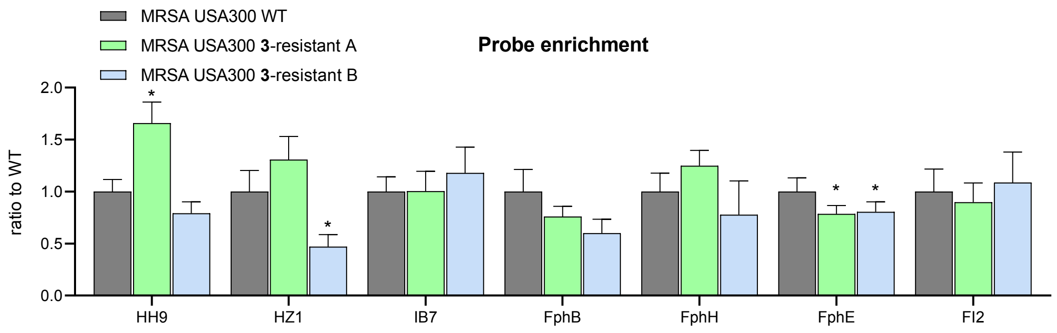

Figure S7. Graph probe enrichment remaining targets. Each group was compared to WT using a two-sided two-sample t-test, n=3 independent experiments per group. Statistical significance: *** P <0.001; ** P < 0.01; * P < 0.05; n.s. if P > 0.05.

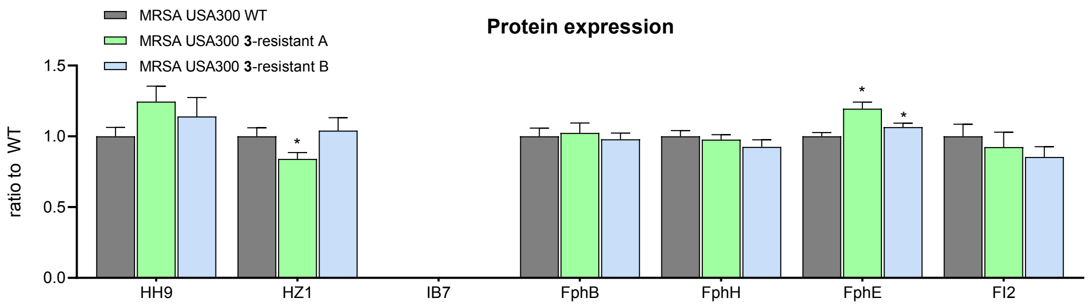

Figure S8. Full graph protein expression. IB7 was not identified. Each group was compared to WT using a two-sided two-sample t-test, n=3 independent experiments per group. Statistical significance: *** P <0.001; ** P < 0.01; * P < 0.05; n.s. if P > 0.05.

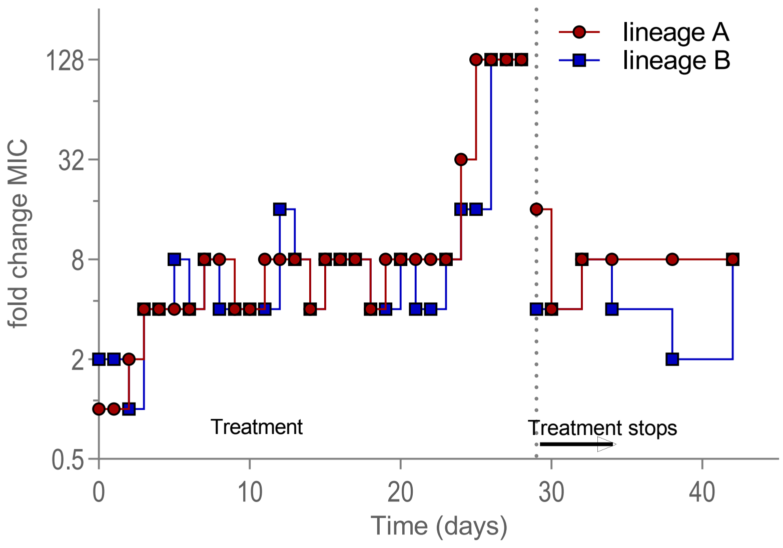

Figure S9. Fold change MIC to compound 3 compared to WT, of daily passage of USA300 cultures for 28 days, followed by antibiotic free subculture for 12 days (dashed line indicates MIC of first drug-free subculture).

Supplementary Data.

Supplementary Data is contained in an Excel file with several tabs. The contents are listed below.

Tab 1. Library hits.

Tab 2. Structure-activity relationship overview.

Tab 3. Extended MIC data.

Tab 4. Chemical proteomics experiments.

A) Initial experiment **3** vs. **4**
B) Experiment **1,2,3,5,6,7** vs. **4**
C) Experiment **3**-resistant A
D) Experiment **3**-resistant B

Tab 5. Full proteome analysis MRSA USA300 WT and **3**-resistant A/B.

Tab 6. Selected gene analysis.

Materials and Methods

Reagents & materials

Buffers and salts were of ACS reagent grade or higher and were purchased commercially, from Carl Roth GmbH (Karlsruhe, Germany) and Sigma-Aldrich (Darmstadt, Germany), biological materials and growth media were purchased from Sigma-Aldrich, Scharlab S.L. (Barcelona, Spain) and Fisher Scientific (Landsmeer, Netherlands). Antibiotics (TRC, Combi-Blocks, Sigma-Aldrich) were dissolved in ultrapure H_2O or DMSO, stock solutions were stored in -20°C, apart from meropenem which was used fresh. All test compounds were used from 10 mM DMSO stock solutions made from freeze dried powder and stored at -20°C.

Bacterial strains

The following reagents were obtained through BEI Resources, NIAID, NIH: *Staphylococcus aureus* BR-VRSA (Strain 880, NR-49120), *S. aureus* strain MRSA131 (HM-466, as part of the Human Microbiome Project). The following *S. aureus* strains were provided by the Network on Antimicrobial Resistance in *S. aureus* (NARSA) for distribution by BEI Resources, NIAID, NIH: COL (NR-45906), NY-155 (NR-46236), USA300-0114 (NR-46070), LIM 2 (NR-45881), LIM 3 (NR-45882), NRS126 (NR-45929), NRS17 (Strain HIP06297, NR-45868), SA MER (NR-45864), VRSA-1 (Strain HIP11714, NR-46410), VRSA-2 (Strain HIP11983, NR-46411) VRSA-3a (Strain HIP13170, NR-46412), and the JE2 Transposon Mutants: NE114 (SAUSA300_0151, NR-46657), NE204 (SAUSA300_1194, NR-46747), NE104 (SAUSA300_0641, NR-46647), NE1534 (SAUSA300_0070, NR-48076), NE1227 (SAUSA300_0560, NR-47770), NE532 (SAUSA300_2473, NR-47075) NE1122 (SAUSA300_0763, NR-47665), NE1187 (SAUSA300_0321, NR-47730), NE31 (SAUSA300_2093, NR-46574). *S. aureus* USA300 (ATCC BAA1717), *S. aureus* Rosenbach (ATCC 29213) *Klebsiella pneumoniae* ATCC 29665 (NCTC 11228), *Escherichia coli* ATCC 25922, *Pseudomonas aeruginosa* ATCC 27853; *Acinetobacter baumannii* ATCC BAA747 belong to the American Type Culture Collection (ATCC). *Enterococcus faecium* E980; *E. faecium* E155; *E. faecium* E7130, were kindly provided by Dr. A. P. A. Hendrickx, National Institute for Health and Environment, Utrecht, The Netherlands, as gifts.

Hemolytic activity

Whole defibrinated sheep blood (10631715, Fisher Scientific) was centrifuged (400 rcf) for 15 min at 4°C. The supernatant was discarded and the remaining blood cell suspension was mixed with phosphate buffered saline (PBS) and centrifuged (400 rcf) for 15 minutes at 4°C. Washing cycles were repeated at least three times, until the supernatant was clear after centrifugation. The packed blood cells were diluted 25-fold in PBS with 0.002% polysorbate 80 (p80). Test compounds were serially diluted 2-fold in U-bottom polypropylene 96-well microtiter plates in PBS with 0.002% p80 (75 µL). An equal volume (75 µL) of the blood cell suspension was added to all wells. Final concentrations of antibiotics ranged from 1.6 µM to 50 µM in triplicate. The well plates were incubated for 20 h at 37°C with continuous shaking (500 rpm).

After incubation, plates were centrifuged (800 rcf) for 5 minutes, and 25 µL of supernatant was transferred to a clear UV-star flat-bottom polystyrene 96-well plate containing 100 µL ultrapure water, per well. Absorption was measured at 415 nm with a Spark® multimode microplate reader (Tecan, Switzerland). Data were corrected for the background response of 0.5% DMSO in the presence of cells with no antibiotic and normalized using the absorbance of 0.1% Triton X-100 with blood cells, as 100% hemolytic activity.

Library screen

From glycerol stocks, USA300, as the Gram-positive representative strain, and W3110, as the Gram-negative representative strain, were cultured on blood agar plates (PB5039A, Thermo Scientific) by overnight (18 ± 2 h) aerobic incubation at 37°C. A single colony was transferred to tryptic soy broth (TSB, 02-200-500, Scharlab). The cultures were grown to exponential phase (OD_{600} = 0.5) at 37°C. The bacterial suspensions were diluted 200-fold in cation adjusted Mueller-Hinton broth (CAMHB) and 99 µL were added in a library of test compounds (1 µL DMSO stock solution, per well in technical duplicates) in polypropylene 96-well microtiter plates to reach a volume of 100 µL and a final concentration of 100 µM for each test compound and a maximum of 1% DMSO. The plates were sealed with breathable membranes and incubated at 37°C for 18 ± 2 h with constant shaking (600 rpm). Screening hits were selected from the wells where no visible bacterial growth was observed, as compared to the inoculum controls, containing 1% DMSO.

Minimum inhibitory concentration (MIC)

From glycerol stocks, bacterial strains were cultured on blood agar plates by overnight incubation at 37°C. A single colony was transferred to TSB. In case of VRSA strains, 6 µg/mL vancomycin was supplemented to the media. The cultures were grown to exponential phase (OD_{600}: 0.5) at 37°C. The bacterial suspensions were diluted 100-fold in CAMHB and 50 µL was added to a 2-fold serial dilution series of test compounds (50 µL per well) in polypropylene 96-well microtiter plates to reach a volume of 100 µL. The plates were sealed with breathable membranes and incubated overnight at 37°C with constant shaking (600 rpm). For *Enterococci species* direct colony suspension was used by immediately suspending multiple colonies from fresh blood agar plates in CAMHB to an OD_{600} of 0.5 and subsequent 100-fold dilution. The MIC was determined as the lowest concentration at which no visible bacterial growth was observed, as compared to the inoculum controls, from the median of a minimum of triplicates.

Minimum bactericidal concentration (MBC)

For minimum bactericidal concentration (MBC) determination, 96-well plates were prepared likewise in biological duplicate. After incubation, 100 µL of each bacterial culture corresponding to 1, 2, 4, 8 and 16× MIC was centrifuged for 5 min (12500 rpm). The supernatant was discarded and pellets were washed once with filter-sterilized PBS, then resuspended in an equal volume of fresh buffer and samples were diluted with a 10-fold factor. 10 µL of the appropriate dilutions were inoculated on Lysogeny Broth (LB) agar plates

in technical duplicates, allowed to evaporate and incubated at 37°C for 18 h. The colonies were counted and used to calculate the CFU/mL of the original culture. The MBC was determined as the lowest concentration of the test compound that was able to produce a 99.9% decrease in viable bacterial cells.

Time-kill assay

From glycerol stocks, bacterial strains were cultured on blood agar plates by overnight incubation at 37°C. Subsequently, a single colony was cultured in TSB overnight at 37°C. The culture was diluted 100-fold in fresh CAMHB and grown until early exponential phase (OD_{600}: 0.25) followed by 100-fold dilution in media (OD_{600}: 0.0025). The culture was split in separate culture tubes containing 2 mL. Test compounds were added to the cultures at concentration 3.1 µM and 6.2 µM (corresponding to 4 and 8× MIC) and incubated at 37°C for a total of 24 h. At indicated time points (t: 0, 1/2, 1, 2, 4, 8 and 24 h) 100 µL of each culture was centrifuged for 5 min (12500 rpm). The supernatant was discarded and pellets were washed once with filter-sterilized PBS, then resuspended in an equal volume of fresh buffer and samples were 10-fold serially diluted until 10^5 dilution. 10 µL of the appropriate dilutions were inoculated on LB agar plates in technical duplicates, allowed to evaporate and incubated at 37°C for 18 h. 10µL of the appropriate dilutions were inoculated on LB agar plates in technical duplicates, subsequently allowed to evaporate and incubated at 37°C for 18 ± 2 h. The colonies were counted and used to calculate the CFU/mL remaining in the original culture by taking the dilution factors into account. Experiment was performed in biological duplicates.

Resistance induction assay

From glycerol stocks, bacterial strains were cultured on blood agar plates by overnight incubation at 37°C. A single colony was grown to exponential phase ($OD_{600} = 0.5$) in TSB and diluted 100-fold in fresh media. In polypropylene 96-well microtiter plates, antibiotics were added in biological triplicates and serial diluted 2-fold by transfer and mixing from one well to the next to achieve a final volume of 50 µL per well. An equal volume of bacterial suspension was added to the wells and plates were incubated at 37°C for 18 h. Bacterial cultures corresponding to 0.25× MIC were diluted 100-fold in fresh media and added (50 µL per well) to a newly prepared antibiotic dilution series (50 µL per well) followed by incubation at 37°C for 18 h. This procedure was repeated for 30 days and the MIC was recorded daily. The experiment was performed in biological triplicates and for each replicate the MIC was determined from the median of a minimum of triplicates.

Mammalian cell culture

HepG2 and HEK293T cell lines (ATCC) were cultured at 37 °C and 7% CO_2 in DMEM (Sigma Aldrich, D6546) with GlutaMax, penicillin (100 µg ml^{-1}), streptomycin (100 µg ml^{-1}) and 10% Fetal Calf serum. Cells were passaged twice a week by first detaching using 0.05% trypsin in PBS, and then diluting to appropriate confluence.

Cytotoxicity assay (MTT)

Compound cytotoxicity was evaluated against HepG2 and HEK293T human cell lines using standard (3-(4,5-dimethylthiazol-2-yl)−2,5-diphenyltetrazolium bromide (MTT) assay protocol[1] with slight changes.

HepG2 and HEK293T cells were seeded at a density of $1.5×10^4$ cells per well in a clear 96-well tissue culture treated plate in a final volume of 100 µL in Dulbecco's Modified Eagle Medium (DMEM), supplemented with Fetal Bovine Serum (1%), Glutamax and Pen/Strep.

Cells were incubated for 24 h at 37°C, 7% CO_2 to allow cells to attach to the plates. In addition to a single vehicle control, compounds (diluted from DMSO stock) were added into each well at eight concentrations ranging from 100 µM to 0.046 µM in three-fold dilutions (final DMSO concentration 0.5%). Incubation was done for 24 h at 37°C, 7% CO_2. After the incubation, MTT was added to each well at a final concentration of 0.40 mg/mL. The plates were then incubated for 2 h at 37°C, 7% CO_2. Medium was carefully removed via suction, and purple formazan crystals were resuspended in 100 µL DMSO.

Absorbance was read at 570 nm using a Clariostar plate reader. The data was then analysed with GraphPad Prism software. IC_{50} values were calculated using non-linear fitted curve with variable slope settings, with values adjusted for background (plotted $ABS_{SAMPLE} = (ABS_{SAMPLE} – ABS_{BACKGROUND}) / (ABS_{VEHICLE} – ABS_{BACKGROUND})$). Technical triplicates for each condition were used, along with two biological replicates. The reported IC_{50} was obtained by averaging the calculated IC_{50} of both biological replicates.

Sample preparation ABPP

Several bacterial colonies were suspended in LB medium in sterile Erlenmeyer flasks and grown in aerobic conditions at 37°C shaking at 270 rpm. At an OD600 of at least 0.70, the cells were divided in 50 mL fractions and harvested by centrifugation (5000 rcf, 10 min, 4°C), and were then washed with PBS once. For each fraction, the pellet was resuspended in 1000 µl PBS, then the samples were pooled and divided into 396 µl samples in 1.5 mL Eppendorf tubes. To each sample 1.5 µl DMSO or inhibitor (200× concentrated) was added and the samples were incubated (600 rpm, 37°C, 1 h). 1.5 µl probe (200× concentrated, final concentration 3 µM) or DMSO was then added and the samples were incubated (37°C, 600 rpm, 30 min).

Cells were pelleted by centrifugation (5000 rcf, 10 min, 4°C) and washed with PBS once. Pellets were then resuspended in 300 µl PBS/0.2% SDS (+ Roche cOmplete protease inhibitor cocktail) and lysed by bead beating (3× 50 s at 6 m/s).

Gel-based ABPP and in-gel fluorescence analysis

18 µl of lysate was clicked with Alexa647-azide (Invitrogen, A10277) by adding 2 µl click mix (10×, 10 mM $CuSO_4$, 56.56 mM sodium ascorbate, 2 mM THPTA, 40 µM Alexa647-azide in Milli-Q) and incubating at rt for 1 h. Note: prepare the click mix separately and in the sequence listed. The reaction was quenched by addition of 5 µl Laemmli buffer (4×, 240 mM Tris-HCl pH

6.8, 8% w/v SDS, 40% glycerol, 5% v/v β-mercapto-ethanol, 0.04% v/v bromophenol blue), followed by heating (95°C, 5 min). The samples were resolved by SDS-PAGE (12.5% acrylamide gel, 15-wells, 10 µL per well) at 180V for 80 min, after which the gels were imaged at Cy3 and Cy5 channels on a ChemiDoc Imaging System (Bio-Rad). Gels are controlled for equal protein loading with Coomassie Brilliant Blue staining. Images were processed using ImageLab software (Bio-Rad).

Preparation for LC-MS based ABPP

Sample preparation was performed according to literature[2] with slight changes. 275 µl of lysate was clicked with biotin-azide (Cayman Chemical, 13040) by adding 25 µl click mix (10×, 10 mM $CuSO_4$, 56.56 mM sodium ascorbate, 2 mM THPTA, 0.4 mM biotin-azide in Milli-Q) and incubating at rt for 1 h. Note: prepare the click mix separately and in the sequence listed.

To each sample 170 µl Milli-Q was added to create a final volume of 500 µl. In the following steps samples were vortexed after each addition. First, 666 µl methanol was added then 166 µl chloroform and finally 150 µl of Milli-Q. Samples were centrifuged (10 min, 1500 g), and solvents were carefully removed respecting the integrity of the formed protein pellet. Methanol (600 µL) was added and the pellet was resuspended using a probe sonicator (30% amplitude, 10 s). The samples were centrifuged (5 min, 18,400 g), and the solvent was removed, again carefully. The residual protein pellet was then dissolved in urea buffer (250 µL, 6 M urea, 250 mM NH_4HCO_3) by means of pipetting.

To each sample DTT (2.5 µL 1 M stock, final concentration 10 mM) was added, followed by incubation at 65°C for 15 minutes while shaking (600 rpm). Samples were cooled to room temperature and iodoacetamide (20 µL 0.5 M stock, final concentration 40 mM) was added, afterwards keeping the samples dark for 15 minutes. SDS (70 µL 10% stock, final concentration 2%) was added, and the samples were incubated for 5 minutes at 65°C while shaking (600 rpm). 1.2 mL of avidin agarose beads (ThermoFisher, 20219) were divided over four 15 mL tubes, and washed with PBS (by adding PBS (10 mL per tube), centrifugation (2 min, 2500 g) and removal of the PBS. Bead solution was made by adding PBS (6 mL) to each of the four tubes. Each of the SDS treated samples were transferred to a 15 mL tube, along with bead solution (1 mL) and PBS (2 mL). The sample containing tubes were rotated for 3 h using an overhead shaker. After shaking, the samples were centrifuged (2 min, 2500 g) and the supernatant was removed. Samples were then resuspended in 0.5% SDS in PBS (6 mL), followed by similar centrifugation (2 min, 2500 g) and removal of supernatant. This process was then repeated thrice with PBS (6 mL), leaving a washed bead pellet. On-bead digestion buffer (250 µL, 100 mM Tris pH 8.0, 100 mM NaCl, 1 mM $CaCl_2$, 2% acetonitrile) was added to the each of the bead residues, and the beads were transferred to low binding tubes (1.5 mL, Sarstedt). Each sample was treated with trypsin solution (1 µL, 0.5 µg/µL Sequencing Grade Modified Trypsin, Porcine (Promega) in 0.1 mM HCl), and the samples were incubated at 37°C overnight while shaking (950 rpm).

To each sample formic acid (12.5 µL) was added, followed by filtering off the beads over biospin columns (Bio-Rad, 7326204) on top of 2 mL Eppendorf tubes using centrifugation (2 min, 300 g). Note: the 2 mL Eppendorf tubes now contain sample solution. StageTips were used for subsequent desalting of the samples. StageTips are punched through holes in Eppendorf tubes, which collect flow-through and each individual step is followed by centrifugation (2 min, 300 g). The StageTips were treated by first conditioning with MeOH (50 µL), washing with solution B (50 µL, 80% v/v acetonitrile, 0.5% v/v formic acid in Milli-Q), and solution A (0.5% v/v formic acid in Milli-Q). The sample solution is then loaded through StageTips, followed by a wash with solution A (50 µL). The StageTips are then transferred to low binding tubes, and the tips are flushed with solution B (100 µL). The collected flow-through is concentrated *in vacuo* using a SpeedVac (Eppendorf Concentrator 5301) at 45°C for 3 h. The samples are stored at at -20°C until LC-MS measurement.

Preparation for full proteome analysis

Bacterial cell lysate was obtained following the 'Sample preparation ABPP' protocol, with minor changes: 1) LB medium was supplemented with either DMSO or 0.78 µM inhibitor during initial bacterial culture; 2) after harvesting, the cells were directly lysed.

Following cell lysis, protein precipitation was performed as described, and protein concentration was determined using BCA assay. Per sample, an amount of protein solution was taken corresponding to 250 µg of protein, and this was diluted with urea buffer to 1 mg/mL protein (250 µL) total. This was followed by adding DTT (5 mM final concentration), and shaking (15 min, 900 rcf, 65°C). After this IAA (40 mM final concentration) was added and the samples were incubated in the dark for 30 min.

For digestion, 100 µL of sample was transferred to a LoBind Eppendorf tube and diluted with 500 µL OB-DIG buffer, and 1 µg trypsin (2 µL, 0.5 µg/uL solution in 1 mM HCl, Promega) was added. The samples were incubated o/n (950 rcf, 37°C). Formic acid (50 µL) was added to quench the reaction and the samples were desalted as reported in 'Preparation for LC-MS based ABPP'.

LC-MS measurement and analysis

Desalted peptide samples were reconstituted in LC-MS solution (3% v/v acetonitrile, 0.1% v/v formic acid in Milli-Q, 50 µL for ABPP samples, 100 µL for full proteome samples) containing 10 fmol/µL yeast enolase digest (cat. 186002325, Waters). Injection amount was titrated using a pooled quality control sample to prevent overloading the nanoLC system and the automatic gain control (AGC) of the QExactive mass spectrometer.

The desalted peptides were separated on an UltiMate 3000 RSLCnano system set in a trap-elute configuration with a nanoEase M/Z Symmetry C18 100 Å, 5 µm, 180 µm × 20 mm (Waters) trap column for peptide loading/retention and nanoEase M/Z HSS C18 T3 100 Å, 1.8 µm, 75 µm × 250 mm (Waters) analytical column for peptide separation. The column was kept at 40 °C in a column oven.

Samples were injected on the trap column at a flow rate of 15 µL/min for 2 min with 99% mobile phase A (0.1% FA in ULC-MS grade water (Biosolve)), 1% mobile phase B (0.1% FA in ULC-MS grade acetonitrile (Biosolve)) eluent.

For ABPP experiments, an 85 min LC method, using mobile phase A and mobile phase B controlled by a flow sensor at 0.3 µL/min with average pressure of 400-500 bar (5500-7000 psi), was programmed as gradient with linear increment to 1% B from 0 to 2 min, 5% B at 5 min, 22% B at 55 min, 40% B at 64 min, 90% B at 65 to 74 min and 1% B at 75 to 85 min. The eluent was introduced by electro-spray ionization (ESI) via the nanoESI source (Thermo) using stainless steel Nano-bore emitters (40 mm, OD 1/32", ES542, Thermo Scientific).

For full proteome experiments, a 123 min LC method, using mobile phase A and mobile phase B controlled by a flow sensor at 0.3 µL/min with average pressure of 400-500 bar (5500-7000 psi), was programmed as gradient with linear increment to 1% to 5% B from 0 to 2 min, 5% to 13% B from 2 to 63 min, 13% to 22% B from 63 to 85 min, 22% to 40% B from 85 to 104 min, 90% at 105 min and kept at 90% to 113 min. The eluent was introduced by electro-spray ionization (ESI) via the nanoESI source (Thermo) using stainless steel Nano-bore emitters (40 mm, OD 1/32", ES542, Thermo Scientific).

The QExactive HF was operated in positive mode with data dependent acquisition without the use of lock mass, default charge of 2+ and external calibration with LTQ Velos ESI positive ion calibration solution (88323, Pierce, Thermo) every 5 days to less than 2 ppm. The tune file for the survey scan was set to scan range of 350 – 1400 m/z, 120,000 resolution (m/z 200), 1 microscan, automatic gain control (AGC) of 3e6, max injection time of 100 ms, no sheath, aux or sweep gas, spray voltage ranging from 1.7 to 3.0 kV, capillary temp of 250 °C and an S-lens value of 80. For the 10 data dependent MS/MS events the loop count was set to 10 and the general settings were resolution to 15,000, AGC target 1e5, max IT time 50 ms, isolation window of 1.6 m/z, fixed first mass of 120 m/z and normalized collision energy (NCE) of 28 eV. For individual peaks the data dependent settings were 1.00e3 for the minimum AGC target yielding an intensity threshold of 2.0e4 that needs to be reached prior of triggering an MS/MS event. No apex trigger was used, unassigned, +1 and charges >+8 were excluded with peptide match mode preferred, isotope exclusion on and dynamic exclusion of 10 sec.

In between experiments, routine wash and control runs were done by injecting 5 µl LC-MS solution containing 5 µL of 10 fmol/µL BSA or enolase digest and 1 µL of 10 fmol/µL angiotensin III (Fluka, Thermo)/oxytocin (Merck) to check the performance of the platform on each component (nano-LC, the mass spectrometer (mass calibration/quality of ion selection and fragmentation) and the search engine)

MS data analysis and processing

Raw files were analyzed using MaxQuant software (version 1.6.17.0) with the Andromeda search engine. The following settings were applied: fixed modification: carbamidomethylation (cysteine); variable modification: oxidation (methionine), acetylation (N-terminus);

proteolytic enzyme: trypsin/P; missed cleavages: 2; main search tolerance: 4.5 ppm; MS/MS tolerance: 0.5 Da; false discovery rates: 0.01. The options "LFQ" and "match between runs" (0.7 min match and 20 min alignment time windows) were enabled; "second peptides" was disabled. Searches were performed against the UniProt database for the S. aureus USA300 proteome (Uniprot ID: UP000001939, downloaded 03-05-2019). Data was extracted from the "peptides.txt" and "proteingroups.txt" files.

Pretreatment of the MaxQuant output was performed using Perseus (version 1.6.15.0). Putative contaminants, reverse peptides and peptides only identified by site were deleted. LFQ intensities were log2-transformed and data was filtered for two valid values in at least one group (each group contains triplicates). To solve the problem of missing values, data imputation was performed over the total matrix. For statistical evaluation, -log10(P values) were obtained by a two-sided two sample homoscedastic Student's t-test (Excel: =T.TEST(range1,range2,2,2)).

PCR amplification and sequencing

The genes encoding for the proteins outcompeted by **3** were amplified by polymerase chain reaction (PCR) using the primers in Supplementary Data 6. DNA was extracted from the type strain *S. aureus* USA300 ATCC BAA1717 and its mutants using a classical phenol-chloroform extraction.[3] PCR reactions were performed using Q5® High-Fidelity DNA Polymerase (New England Biolabs) following the manufacturer protocols and their thermocycling conditions. Sequencing of the PCR products was performed at Macrogen Europe BV and sequences were analyzed and compared to the wild type (WT) *S. aureus* USA300 with reference sequence GCF_000013465.1 using SnapGene® version 6.0.

Synthetic procedures

General remarks

All chemicals (Sigma-Aldrich, Fluka, Acros, Merck, Combi-Blocks, Fluorochem, TCI) were used as received. All solvents used for reactions were of analytical grade. THF, Et_2O, DMF, ACN and DCM were dried over activated 4 Å molecular sieves, MeOH over 3 Å molecular sieves. H_2O used in synthesis procedures was of Milli-Q-grade quality. Column chromatography was performed on silica gel (Screening Devices BV, 40-63 µm, 60 Å). The eluent EtOAc was of technical grade and distilled before use. Triethylamine was distilled over KOH, and triethylamine and pyridine were stored over KOH pellets. Starting materials were coevaporated with toluene (3×) before use in water-sensitive reactions.

Reactions were monitored by thin layer chromatography (TLC) analysis using Merck aluminium sheets (Silica gel 60, F254). Compounds were visualized by UV-absorption (254 nm) and spraying for general compounds: $KMnO_4$ (20 g/L) and K_2CO_3 (10 g/L) in H_2O, or for amines: ninhydrin (0.75 g/L) and acetic acid (12.5 mL/L) in ethanol, followed by charring at 150°C. ^{1}H and ^{13}C NMR experiments were recorded on a Bruker AV-300 (300/75 MHz), Bruker AV-400 (400/101 MHz), Bruker DMX-400 (400/101 MHz), Bruker AV- 500 (500/126 MHz) and Bruker AV-600 (600/151 MHz). Chemical shifts are given in ppm (δ) relative to tetramethylsilane, as internal standard. Multiplicity: s = singlet, bs = broad singlet, d = doublet, dd = doublet of doublet, t = triplet, q = quartet, quint = quintet, non = nonet m = multiplet. Coupling constants (*J*) are given in Hz. LC-MS measurements were performed on a Thermo Finnigan LCQ Advantage MAX ion-trap mass spectrometer (ESI+) coupled to a Surveyor HPLC system (Thermo Finnigan) equipped with a standard C18 (Gemini, 4.6 mm D × 50 mm L, 5 µm particle size, Phenomenex) analytical column and buffers A: H_2O, B: ACN, C: 0.1% aq. TFA. High resolution mass spectra were recorded on a LTQ Orbitrap (Thermo Finnigan) mass spectrometer or a Synapt G2-Si high-definition mass spectrometer (Waters) equipped with an electrospray ion source in positive mode (source voltage 3.5 kV, sheath gas flow 10 mL/min, capillary temperature 250°C) with resolution R = 60000 at m/z 400 (mass range m/z = 150-2000) and dioctylphtalate (m/z = 391.28428) as a lock mass. Preparative HPLC was performed on a Waters Acquity Ultra Performance LC with a C18 column (Gemini, 150 × 21.2 mm, Phenomenex) using a ACN in H_2O (+0.2% TFA) gradient. All final compounds were determined to be > 95% pure by LC-UV analysis.

General Procedure A: Amide/carbamate formation from acyl chloride

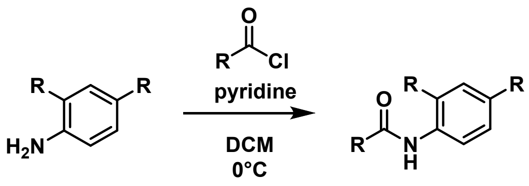

Aniline (1 equiv.) was dissolved in DCM (0.05 M) along with pyridine (1.1 equiv.) and the mixture was cooled to 0°C, after which acyl chloride (1.1 equiv.) was added. The mixture was stirred until TLC analysis indicated full conversion of the starting material. The mixture was then washed with H_2O (3×), and the organic layer was dried ($MgSO_4$), filtered and concentrated *in vacuo* to obtain the crude product.

General Procedure B: HOBt amide coupling

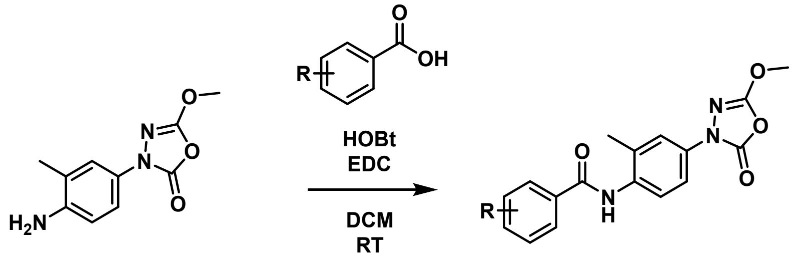

Benzoic acid (1.6 equiv.), EDC (2.7 equiv.) and HOBt (1 equiv.) were dissolved in DCM (0.2 M). The mixture was stirred for 30 min at 0°C before the substituted aniline (1 equiv.) was added and the reaction was stirred until TLC analysis showed full conversion of the aniline derivative. Additional DCM was added and the mixture was washed with 10% aq. $NaHCO_3$ (2×), dried ($MgSO_4$), filtered and concentrated *in vacuo* to obtain the crude product.

General Procedure C: HATU amide coupling

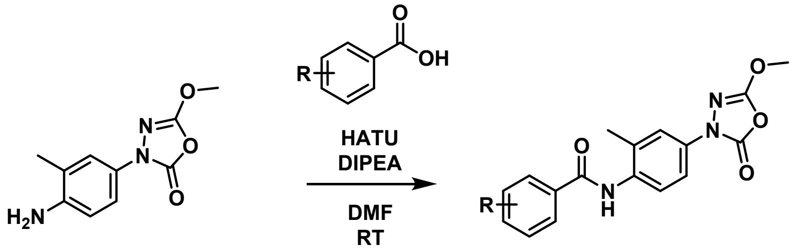

Substituted aniline (1 equiv.), benzoic acid (1.5 equiv.), HATU (2 equiv.) and DIPEA (2 equiv.) were dissolved in DMF (0.1 M), and the mixture was stirred at RT until TLC analysis indicated complete conversion of the starting material. H_2O was added and the aqueous layer was extracted with DCM (2×). The organic layers were combined, dried ($MgSO_4$), filtered, and concentrated *in vacuo* to obtain the crude product.

General Procedure D: PyAOP amide coupling

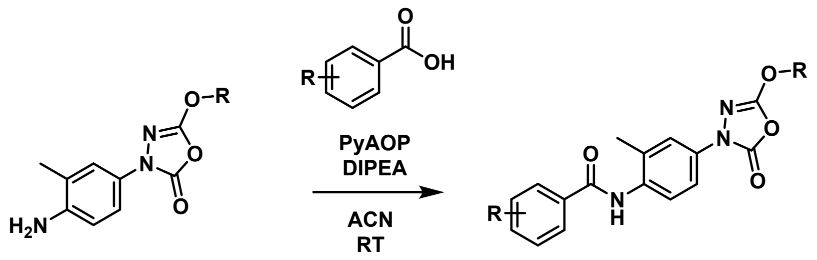

Benzoic acid (2 equiv.), PyAOP (2 equiv.) and DIPEA (4 equiv.) were dissolved in ACN (0.034 M) and the mixture was stirred for 3 min in a dry round bottom flask under nitrogen atmosphere. Substituted aniline (1 equiv.) was dissolved in acetonitrile and this solution was added dropwise to the stirring mixture at RT. The mixture was stirred until TLC analysis indicated full conversion of the starting material. An excess of EtOAc was added and the mixture was washed with brine, sat. aq. $NaHCO_3$ (2×), 1 M aq. HCl and brine again, dried ($MgSO_4$), filtered, and concentrated *in vacuo* to obtain the crude product.

General Procedure E: Oxadiazolone formation

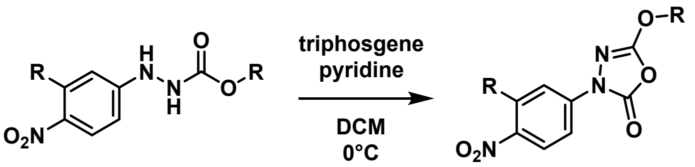

Hydrazine carboxylate (1 equiv.) and pyridine (2 equiv.) were dissolved in DCM (0.2 M), and cooled to 0°C after which a solution of triphosgene (1 equiv.) in DCM (0.5 – 1 M) was added dropwise. The mixture was stirred until TLC analysis indicated full conversion of the starting material. Then, 5% aq. NH_4OH (equal volume to DCM) was added, and the mixture was stirred for 10 min. The resulting organic layer was washed with 1 M aq. HCl, subsequently dried ($MgSO_4$), filtered and concentrated *in vacuo* to obtain the crude product.

General Procedure F: Nitrophenyl hydrogenation

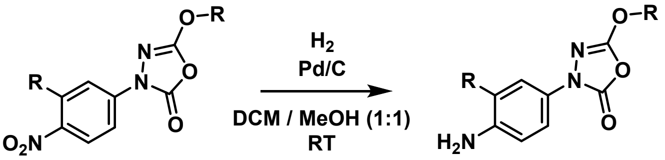

Nitrophenyl (1 equiv.) was dissolved in DCM/MeOH, and under nitrogen atmosphere Pd/C catalyst (± 0.1 equiv.) was added. The mixture was stirred, and hydrogen gas was bubbled through the solution. Once TLC analysis showed complete conversion of the starting material, the atmosphere was displaced with nitrogen, followed by filtering over Celite®, and concentration of the filtrate *in vacuo* to obtain the crude product.

General Procedure G: Chan-Lam coupling

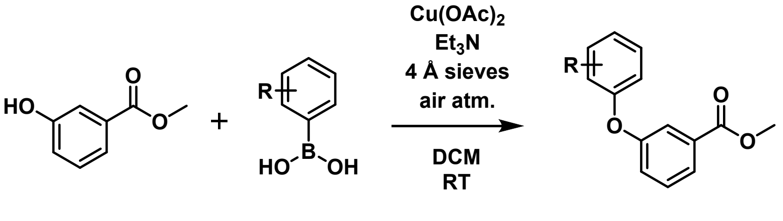

In a round-bottom flask, ethyl-3-hydroxybenzoate (**84**) (1 equiv.) and the corresponding boronic acid (1 equiv.) were dissolved in DCM (0.2 M), after which crushed 4 Å molecular

sieves (1 g/mmol reactant) and $Cu(OAc)_2$ (1 equiv.) were added. Triethylamine (2.5 equiv.) was added and the mixture was stirred for 2 h under air atmosphere. The mixture was washed with 1 M aq. HCl, and sieves were filtered off. The filtrate was dried ($MgSO_4$) and filtered again, followed by concentration *in vacuo* to obtain the crude product.

General Procedure H: Saponification

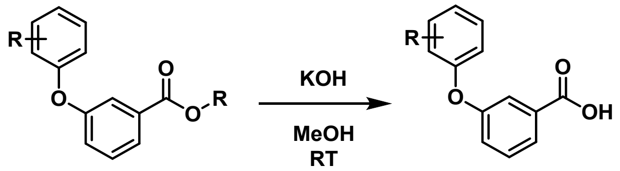

KOH (10 equiv.) was added to a stirring solution of ester (1 equiv.) in MeOH (0.3 M). When TLC indicated complete consumption of the starting ester, the mixture was concentrated *in vacuo*. The crude material was redissolved in H_2O and 50% sulfuric acid was added dropwise until precipitate was fully formed. The suspension was stirred for 30 min and was filtered, washed with H_2O and the residue was isolated and dried *in vacuo*. The resulting benzoic acid was used in the next reaction without further purification.

Benzyl (4-(5-methoxy-2-oxo-1,3,4-oxadiazol-3(2*H*)-yl)-2-methylphenyl)carbamate (1)

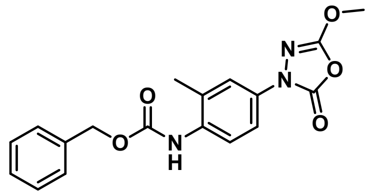

12 (40 mg, 0.18 mmol) and benzyl chloroformate (28 µL, 0.20 mmol) were reacted following General Procedure A. The title compound was obtained as a white solid (59 mg, 0.17 mmol, 92%) without need for further purification.

^{1}H NMR (300 MHz, $CDCl_3$) δ 7.90 – 7.76 (m, 1H), 7.62 – 7.54 (m, 2H), 7.44 – 7.30 (m, 5H), 6.52 (bs, 1H), 5.20 (s, 2H), 4.08 (s, 3H), 2.25 (s, 3H).

^{13}C NMR (75 MHz, $CDCl_3$) δ 155.86, 153.69, 148.35, 136.06, 133.51, 133.47, 128.74, 128.52, 128.48, 119.89, 116.67, 67.32, 57.79, 17.96.zfh

HRMS $[C_{18}H_{17}N_3O_5 + H]^+$: 356.12410 calculated, 356.12409 found.

***N*-(4-(5-Methoxy-2-oxo-1,3,4-oxadiazol-3(2*H*)-yl)-2-methylphenyl)benzamide (2)**

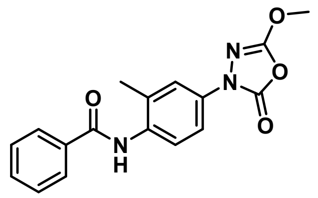

12 (40 mg, 0.18 mmol) was reacted with benzoyl chloride (23 µL, 0.20 mmol) following General Procedure A. The resulting crude material was purified by column chromatography (DCM) to obtain **2** as a white solid. (35 mg, 0.11 mmol, 59%).

^{1}H NMR (400 MHz, $CDCl_3$) δ 8.03 (d, *J* = 8.9 Hz, 1H), 7.93 – 7.86 (m, 2H), 7.72 – 7.62 (m, 2H), 7.62 – 7.55 (m, 1H), 7.55 – 7.47 (m, 2H), 4.12 (s, 3H), 2.38 (s, 3H).

^{13}C NMR (101 MHz, CDCl3) δ 165.68, 155.96, 148.39, 134.89, 133.44, 133.23, 132.16, 130.44, 129.04, 127.18, 123.86, 119.91, 116.65, 57.87, 18.21.

HRMS $[C_{17}H_{15}N_3O_4 + H]^+$: 326.11353 calculated, 326.11306 found.

N-(4-(5-Methoxy-2-oxo-1,3,4-oxadiazol-3(2H)-yl)-2-methylphenyl)-3-phenoxybenzamide (3)

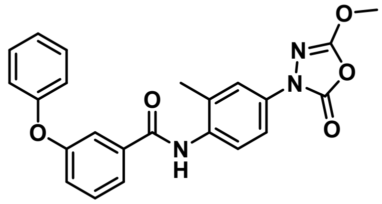

3-Phenoxybenzoic acid (194 mg, 0.904 mmol) was reacted with **12** (100 mg, 0.452 mmol) following General Procedure D. The crude product was purified with column chromatography (20% → 30% EtOAc in pentane) to obtain **3** as a white powder (60 mg, 0.144 mmol, 32%).

^{1}H NMR (400 MHz, $CDCl_3$) δ 8.00 (d, *J* = 8.9 Hz, 1H), 7.71 (s, 1H), 7.68 – 7.60 (m, 2H), 7.58 (d, *J* = 7.7 Hz, 1H), 7.51 (s, 1H), 7.46 (t, *J* = 7.9 Hz, 1H), 7.38 (t, *J* = 7.7 Hz, 2H), 7.22 – 7.13 (m, 2H), 7.06 (d, *J* = 8.0 Hz, 2H), 4.11 (s, 3H), 2.35 (s, 3H).

^{13}C NMR (101 MHz, $CDCl_3$) δ 165.15, 158.27, 156.47, 155.96, 148.38, 136.74, 133.29, 133.27, 130.43, 130.16, 124.22, 123.82, 122.04, 121.40, 119.90, 119.56, 117.26, 116.62, 57.87, 18.17.

HRMS $[C_{23}H_{19}N_3O_5 + H]^+$: 418.13975 calculated, 418.13967.

3-Ethynyl-N-(4-(5-methoxy-2-oxo-1,3,4-oxadiazol-3(2H)-yl)-2-methylphenyl)benzamide (4)

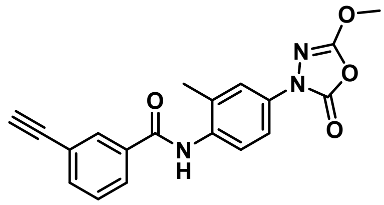

3-Ethynylbenzoic acid (66 mg, 0.452 mmol) was reacted with **12** (50 mg, 0.226 mmol) following General Procedure D. The crude product was purified with column chromatography (0% → 10% MeOH in DCM) to obtain title compound **4** as a white solid (40 mg, 0.114 mmol, 51%).

^{1}H NMR (400 MHz, $CDCl_3$) δ 7.98 (s, 1H), 7.94 – 7.81 (m, 2H), 7.76 – 7.58 (m, 4H), 7.46 (t, *J* = 7.8 Hz, 1H), 4.11 (s, 3H), 3.16 (s, 1H), 2.36 (s, 3H).

^{13}C NMR (101 MHz, $CDCl_3$) δ 164.98, 155.95, 148.36, 135.48, 135.12, 133.50, 133.07, 131.04, 130.69, 129.13, 127.65, 124.27, 123.11, 119.89, 116.54, 82.60, 78.74, 57.89, 18.24.

HRMS $[C_{19}H_{15}N_3O_4 + H]^+$: 350.11353 calculated, 350.11321 found.

N-(4-(5-Methoxy-2-oxo-1,3,4-oxadiazol-3(2H)-yl)-2-methylphenyl)pyrimidine-4-carboxamide (5)

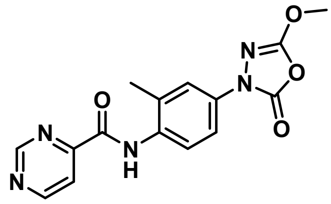

12 (20 mg, 90 µmol) and pyrimidine-4-carboxylic acid (18.7 mg, 0.151 mmol) were reacted following General Procedure B. The resulting crude material was purified by column chromatography (0.1% → 0.5% MeOH in DCM) to obtain **5** as a yellow solid (4.9 mg, 15 µmol, 17%).

^1H NMR (400 MHz, $CDCl_3$) δ 9.96 (s, 1H), 9.33 (d, *J* = 1.4 Hz, 1H), 9.06 (d, *J* = 5.0 Hz, 1H), 8.34 (d, *J* = 8.9 Hz, 1H), 8.24 (dd, *J* = 5.0, 1.4 Hz, 1H), 7.74 (d, *J* = 2.5 Hz, 1H), 7.69 (dd, *J* = 8.9, 2.6 Hz, 1H), 4.12 (s, 3H), 2.47 (s, 3H).

^{13}C NMR (101 MHz, $CDCl_3$) δ 160.24, 159.81, 157.91, 156.49, 155.99, 148.38, 133.09, 132.83, 129.37, 122.11, 119.97, 118.74, 116.73, 57.89, 18.01.

HRMS $[C_{15}H_{13}N_5O_4 + H]^+$: 328.10403 calculated, 328.10384 found.

_N_-(4-(5-Methoxy-2-oxo-1,3,4-oxadiazol-3(2_H_)-yl)-2-methylphenyl)isonicotinamide (6)

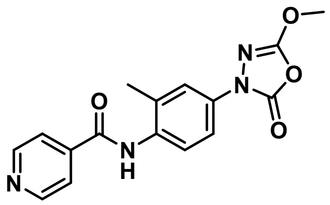

Isonicotinic acid (18.6 mg, 0.151 mmol) and **12** (20 mg, 90 µmol) were reacted following General Procedure B. The resulting crude material was purified by column chromatography (0.1% → 0.5% MeOH in DCM) to obtain **6** as a yellow solid (5.0 mg, 15 µmol, 17%).

^1H NMR (400 MHz, DMSO) δ 10.23 (s, 1H), 8.82 – 8.76 (m, 2H), 7.91 – 7.86 (m, 2H), 7.66 – 7.56 (m, 2H), 7.47 (d, *J* = 8.6 Hz, 1H), 4.09 (s, 3H), 2.29 (s, 3H).

^{13}C NMR (101 MHz, DMSO) δ 163.99, 155.45, 150.38, 148.49, 141.36, 135.00, 134.01, 133.01, 127.45, 121.60, 119.73, 115.53, 58.17, 18.11.

HRMS $[C_{16}H_{14}N_4O_4 + H]^+$: 327.10878 calculated, 327.10850 found.

_N_-(4-(5-Methoxy-2-oxo-1,3,4-oxadiazol-3(2_H_)-yl)-2-methylphenyl)-4-(trifluoromethyl)benzamide (7)

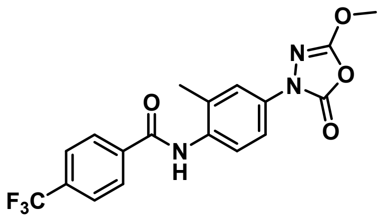

12 (20 mg, 90 µmol) was reacted with 4-(trifluoromethyl)benzoyl chloride (15 µL, 99 µmol) according to General Procedure A. Purification of the crude material by column chromatography (0% → 2% MeOH in DCM) gave the title compound (28 mg, 71 µmol, 79%).

^1H NMR (400 MHz, $CDCl_3$) δ 7.98 (d, *J* = 8.0 Hz, 2H), 7.89 (s, 1H), 7.84 (d, *J* = 8.8 Hz, 1H), 7.73 (d, *J* = 8.1 Hz, 2H), 7.68 (d, *J* = 2.6 Hz, 1H), 7.62 (dd, *J* = 8.8, 2.6 Hz, 1H), 4.12 (s, 3H), 2.34 (s, 3H).

^{13}C NMR (101 MHz, $CDCl_3$) δ 164.72, 155.97, 148.35, 138.00, 133.74 (t, *J* = 15.1 Hz), 132.80, 131.46, 127.75, 125.98 (q, *J* = 3.6 Hz), 125.06, 124.53, 122.35, 119.87, 116.44, 57.91, 18.18.

HRMS $[C_{18}H_{14}F_3N_3O_4 + H]^+$: 394.10092 calculated, 394.10077 found.

Benzyl _o_-tolylcarbamate (8)

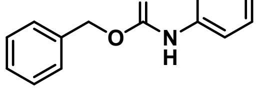

o-Toluidine (107 µL, 1.0 mmol) and benzyl chloroformate (165 µL, 1.1 mmol) were reacted following General Procedure A. Title compound

was obtained as a white solid (242 mg, 1.0 mmol, quant.) without need for further purification.

^{1}H NMR (400 MHz, $CDCl_3$) δ 7.80 (bs, 1H), 7.45 – 7.27 (m, 5H), 7.25 – 7.16 (m, 1H), 7.13 (d, *J* = 7.5, 1H), 7.01 (td, *J* = 7.5, 1.3 Hz, 1H), 6.49 (bs, 1H), 5.19 (s, 2H), 2.21 (s, 3H).

^{13}C NMR (101 MHz, $CDCl_3$) δ 153.78, 136.19, 135.85, 130.50, 128.82, 128.45, 126.97, 124.30, 67.18, 17.75.

HRMS $[C_{15}H_{15}NO_2 + H]^+$: 242.11756 calculated, 242.11762 found.

5-Methoxy-3-(*m*-tolyl)-1,3,4-oxadiazol-2(3*H*)-one (9)

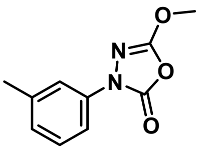

70 (90 mg, 0.50 mmol) and pyridine (0.20 mL) were dissolved in dry DCM (3 mL). The mixture was cooled to 0°C, after which phosgene (20 wt% in toluene, 528 µL, 1.0 mmol) was added dropwise. After stirring for 2 h the mixture was washed with H_2O (3×). The organic layer was dried ($MgSO_4$), filtered, and concentrated *in vacuo*. Purification of the crude material by column chromatography (5% → 10% EtOAc in pentane) afforded the desired product as a yellow oil (60 mg, 0.29 mmol, 58%).

^{1}H NMR (400 MHz, $CDCl_3$) δ 7.63 – 7.55 (m, 2H), 7.29 (t, *J* = 7.8 Hz, 1H), 7.03 (d, *J* = 7.5, 1H), 4.09 (s, 3H), 2.39 (s, 3H).

^{13}C NMR (101 MHz, $CDCl_3$) δ 155.87, 148.39, 139.28, 136.17, 129.03, 126.47, 117.93, 115.18, 60.02, 21.64.

HRMS $[C_{10}H_{10}N_2O_3 + Na]^+$: 229.05836 calculated, 228.96498 found.

Methyl 2-(4-(((benzyloxy)carbonyl)amino)-3-methylphenyl)hydrazine-1-carboxylate (10)

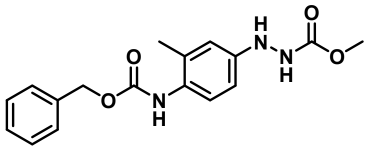

71 (23 mg, 0.12 mmol) and benzyl chloroformate (18 µL, 0.13 mmol) were reacted following General Procedure A. Purification of the crude material by column chromatography (0% → 1% MeOH in DCM) yielded the title compound as a white powder (17 mg, 52 µmol, 45%).

^{1}H NMR (500 MHz, DMSO) δ 9.02 (s, 1H), 8.70 (s, 1H), 7.64 – 7.27 (m, 6H), 6.99 (d, *J* = 7.6 Hz, 1H), 6.60 – 6.31 (m, 2H), 5.09 (s, 2H), 3.59 (s, 3H), 2.09 (s, 3H).

^{13}C NMR (126 MHz, DMSO) δ 157.40, 154.83, 147.15, 137.12, 128.40, 127.85, 127.78 127.31, 127.30, 113.20, 109.57, 65.45, 51.73, 18.01.

HRMS $[C_{17}H_{19}N_3O_4 + Na]^+$: 352.12678 calculated, 352.12661 found.

Benzyl (4-(5-methoxy-2-oxo-1,3,4-oxadiazol-3(2*H*)-yl)phenyl)carbamate (11)

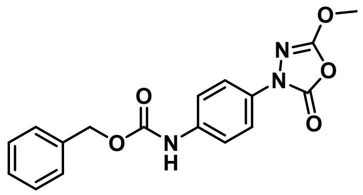

67b (35 mg, 0.17 mmol) and benzyl chloroformate (27 µL, 0.19 mmol) were reacted following General Procedure A. The title compound was obtained as a yellow solid (58 mg, 0.15 mmol, 90%) without further need for purification.

^{1}H NMR (400 MHz, $CDCl_3$) δ 7.73 – 7.66 (m, 2H), 7.43 (d, *J* = 8.5 Hz, 2H), 7.39 – 7.27 (m, 5H), 6.91 (bs, 1H), 5.19 (s, 2H), 4.08 (s, 3H).

^{13}C NMR (101 MHz, $CDCl_3$) δ 155.88, 153.43, 148.37, 136.02, 135.52, 131.78, 128.72, 128.48, 128.37, 125.95, 118.89, 67.19, 57.80.

HRMS $[C_{17}H_{15}N_3O_5 + H]^+$: 342.10845 calculated, 342.10844 found.

3-(4-Amino-3-methylphenyl)-5-methoxy-1,3,4-oxadiazol-2(3*H*)-one (12)

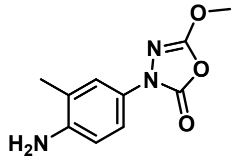

66a (470 mg, 1.87 mmol) was dissolved in DCM (25 mL) and MeOH (15 mL), and was reacted with Pd/C catalyst (10 wt%, 150 mg, 0.14 mmol) following General Procedure F. This yielded the title compound as an off-white solid (408 mg, 1.84 mmol, 99%) without further purification.

^{1}H NMR (500 MHz, $CDCl_3$) δ 7.40 (d, *J* = 2.5 Hz, 1H), 7.36 (dd, *J* = 8.5, 2.5 Hz, 1H), 6.67 (d, *J* = 8.5 Hz, 1H), 4.05 (m, 3H), 3.65 (bs, 2H), 2.19 (s, 3H).

^{13}C NMR (126 MHz, $CDCl_3$) δ 155.65, 148.65, 143.03, 127.28, 122.90, 121.33, 118.08, 114.94, 57.59, 17.51.

HRMS $[C_{10}H_{11}N_3O_3 + H]^+$: 222.08732 calculated, 222.08730 found.

Methyl (4-(5-methoxy-2-oxo-1,3,4-oxadiazol-3(2*H*)-yl)-2-methylphenyl)carbamate (13)

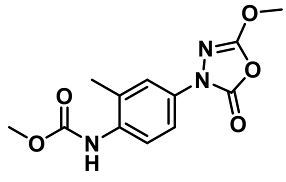

12 (40 mg, 0.18 mmol) and methyl chloroformate (15 µL, 0.20 mmol) were reacted following General Procedure A. The title compound was obtained as a white solid (50 mg, 0.18 mmol, 99%) without need for further purification.

^{1}H NMR (500 MHz, $CDCl_3$) δ 7.80 (bs, 1H), 7.61 (d, *J* = 2.6, Hz, 1H), 7.59 (dd, *J* = 8.7, 2.6 Hz, 1H), 6.45 (bs, 1H), 4.10 (s, 3H), 3.78 (s, 3H), 2.28 (s, 3H).

^{13}C NMR (101 MHz, $CDCl_3$) δ 155.89, 154.46, 148.38, 133.52, 132.30, 132.16, 119.91, 116.70, 57.81, 52.61, 17.95.

HRMS $[C_{12}H_{13}N_3O_5 + H]^+$: 280.09280 calculated, 280.09272 found.

1-Benzyl-3-(4-(5-methoxy-2-oxo-1,3,4-oxadiazol-3(2*H*)-yl)-2-methylphenyl)urea (14)

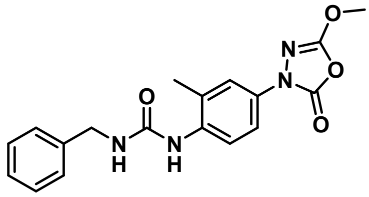

12 (40 mg, 0.18 mmol) and triethylamine (25 µL, 0.18 mmol) were dissolved in DCM (2 mL). The mixture was cooled to 0°C,

after which a solution of triphosgene (32 mg, 0.11 mmol) in DCM (2 mL) was added dropwise. After stirring for 15 min, a solution of benzylamine (58 mg, 0.54 mmol) in DCM (2 mL) was added. 3 h later more triethylamine was added (51 µL, 0.36 mmol), and 2 h hereafter the reaction mixture was washed with 1 M aq. HCl and 5% aq. NH_4OH. The organic layer was dried ($MgSO_4$), filtered and concentrated *in vacuo*. Purification of the residue by preparative HPLC was performed to give the final product as a white solid (12 mg, 34 µmol, 19%).

^{1}H NMR (400 MHz, DMSO) δ 7.94 (dd, *J* = 8.9, 2.5 Hz, 1H), 7.83 (s, 1H), 7.51 – 7.21 (m, 6H), 7.04 (t, *J* = 5.8 Hz, 1H), 6.68 (bs, 1H), 4.31 (d, *J* = 5.8 Hz, 2H), 4.06 (s, 3H), 2.22 (s, 3H).

^{13}C NMR (101 MHz, DMSO) δ 171.50, 155.34, 148.06, 140.18, 135.96, 130.05, 128.40, 127.75, 127.29, 126.85, 120.81, 119.84, 116.19, 58.08, 42.91, 18.09.

HRMS $[C_{18}H_{18}N_4O_4 + H]^+$: 355.14008 calculated, 355.13990 found.

_N_-(4-(5-Methoxy-2-oxo-1,3,4-oxadiazol-3(2*H*)-yl)-2-methylphenyl)-3-phenylpropanamide (15)

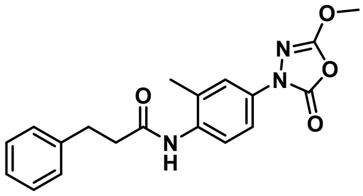

12 (40 mg, 0.18 mmol) and hydrocinnamoyl chloride (30 µL, 0.20 mmol) were reacted following General Procedure A. Purification of the crude material by column chromatography (0% → 0.25% MeOH in DCM) yielded the title compound as a white powder (39 mg, 0.11 mmol, 61%).

^{1}H NMR (400 MHz, $CDCl_3$) δ 7.69 (d, *J* = 8.7 Hz, 1H), 7.55 (d, *J* = 2.5 Hz, 1H), 7.52 (dd, *J* = 8.7, 2.6 Hz, 1H), 7.34 – 7.17 (m, 5H), 7.02 (s, 1H), 4.09 (s, 3H), 3.05 (t, *J* = 7.5 Hz, 2H), 2.70 (t, *J* = 7.5 Hz, 2H), 2.05 (s, 3H).

^{13}C NMR (101 MHz, $CDCl_3$) δ 170.69, 155.88, 148.32, 140.61, 133.12, 133.08, 130.69, 128.80, 128.53, 126.58, 124.20, 119.71, 116.33, 57.84, 39.23, 31.80, 17.85.

HRMS $[C_{19}H_{19}N_3O_4 + H]^+$: 354.14483 calculated, 354.14464 found.

Phenyl (4-(5-methoxy-2-oxo-1,3,4-oxadiazol-3(2*H*)-yl)-2-methylphenyl)carbamate (16)

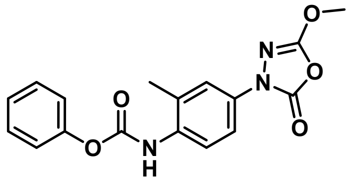

12 (40 mg, 0.18 mmol) and phenyl carbonochloridate (25 µL, 0.20 mmol) were reacted following General Procedure A. The resulting crude material was purified by column chromatography (DCM). The product was additionally redissolved in DCM, washed with 1 M aq. HCl, dried ($MgSO_4$) and concentrated *in vacuo* to obtain **16** as a white solid (28 mg, 82 µmol, 45%).

^{1}H NMR (400 MHz, DMSO) δ 9.57 (s, 1H), 7.60 – 7.52 (m, 3H), 7.47 – 7.39 (m, 2H), 7.30 – 7.20 (m, 3H), 4.08 (s, 3H), 2.35 (s, 3H).

^{13}C NMR (101 MHz, DMSO) δ 155.42, 152.76, 150.78, 148.03, 133.26, 132.88, 129.43, 125.38, 121.92, 119.44, 115.73, 58.15, 18.07.

HRMS $[C_{17}H_{17}N_3O_5 + H]^+$: 342.10845 calculated, 342.10835 found.

_N_-(4-(5-Methoxy-2-oxo-1,3,4-oxadiazol-3(2_H_)-yl)-2-methylphenyl)-2-phenylacetamide (17)

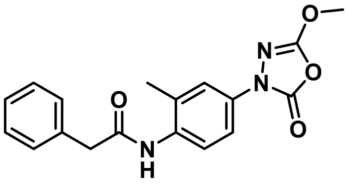

12 (40 mg, 0.18 mmol) was reacted with 2-phenylacetyl chloride (26 µL, 0.20 mmol) according to General Procedure A. The resulting crude material was purified by column chromatography (0% → 0.5% MeOH in DCM). The purified compound was redissolved in DCM, washed with sat aq. $NaHCO_3$ (2×), dried ($MgSO_4$), filtered, and concentrated *in vacuo* to obtain **17** as a white solid (43 mg, 0.13 mmol, 70%).

^{1}H NMR (400 MHz, $CDCl_3$) δ 7.98 – 7.89 (m, 1H), 7.56 (m, 2H), 7.47 – 7.32 (m, 5H), 6.97 (s, 1H), 4.09 (s, 3H), 3.79 (s, 2H), 1.93 (s, 3H).

^{13}C NMR (101 MHz, $CDCl_3$) δ 169.14, 155.77, 148.21, 134.51, 133.10, 132.76, 130.46, 129.70, 129.35, 127.94, 123.38, 119.57, 116.37, 57.73, 44.82, 16.95.

HRMS $[C_{18}H_{17}N_3O_4 + H]^+$: 340.12918 calculated, 340.12908 found.

_N_-(4-(5-Methoxy-2-oxo-1,3,4-oxadiazol-3(2_H_)-yl)phenyl)benzamide (18)

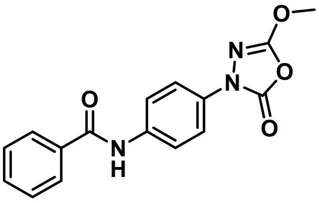

67b (11 mg, 84 µmol) was reacted with benzoyl chloride (6 µL, 53 µmol) using solely pyridine (0.7 mL) as solvent following General Procedure A. Purification of the crude material by column chromatography (0% → 2% MeOH in DCM) gave the final compound as a yellow solid (11 mg, 35 µmol, 67%).

^{1}H NMR (400 MHz, DMSO) δ 10.39 (s, 1H), 7.99 – 7.95 (m, 2H), 7.92 – 7.86 (m, 2H), 7.71 – 7.66 (m, 2H), 7.63 – 7.57 (m, 1H), 7.56 – 7.49 (m, 2H), 4.08 (s, 3H).

^{13}C NMR (101 MHz, DMSO) δ 165.52, 155.42, 148.02, 136.65, 134.76, 131.68, 131.57, 128.43, 127.68, 120.98, 118.19, 58.12.

HRMS $[C_{16}H_{13}N_3O_4 + H]^+$: 312.09788 calculated, 312.09768 found.

_N_-(2-Methoxy-4-(5-methoxy-2-oxo-1,3,4-oxadiazol-3(2_H_)-yl)phenyl)benzamide (19)

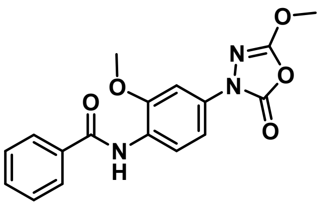

67c (20 mg, 84 µmol) was reacted with benzoyl chloride (11 µL, 93 µmol) according to General Procedure A. Purification of the crude material by column chromatography (0% → 2% MeOH in DCM) gave the title compound as a yellow solid (25 mg, 73 µmol, 87%).

^{1}H NMR (400 MHz, $CDCl_3$) δ 8.59 (d, *J* = 8.9 Hz, 1H), 8.51 (s, 1H), 7.93 – 7.84 (m, 2H), 7.63 – 7.45 (m, 4H), 7.37 (dd, *J* = 8.9, 2.4 Hz, 1H), 4.11 (s, 3H), 3.97 (s, 3H).

^{13}C NMR (101 MHz, $CDCl_3$) δ 165.27, 155.85, 148.52, 148.32, 135.12, 132.23, 131.94, 128.90, 127.13, 125.45, 120.07, 110.44, 100.68, 57.90, 56.24.

HRMS $[C_{17}H_{15}N_3O_5 + H]^+$: 342.10845 calculated, 342.10832 found.

_N_-(2-Chloro-4-(5-methoxy-2-oxo-1,3,4-oxadiazol-3(2_H_)-yl)phenyl)benzamide (20)

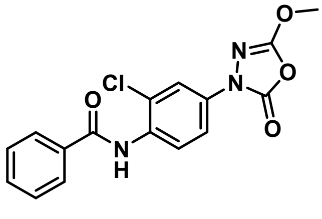

67d (19 mg, 79 µmol) was reacted with benzoyl chloride (10 µL, 86 µmol) following General Procedure A using solely pyridine (0.7 mL) as solvent. Purification of the crude material by column chromatography (0% → 2% MeOH in DCM) gave the final compound as a yellow solid (7.0 mg, 20 µmol, 26%).

^{1}H NMR (400 MHz, $CDCl_3$) δ 8.67 (d, *J* = 9.1 Hz, 1H), 8.46 (s, 1H), 7.98 (d, *J* = 2.5 Hz, 1H), 7.97 – 7.92 (m, 2H), 7.80 (dd, *J* = 9.1, 2.5 Hz, 1H), 7.66 – 7.60 (m, 1H), 7.60 – 7.52 (m, 2H), 4.16 (s, 3H).

^{13}C NMR (101 MHz, $CDCl_3$) δ 165.29, 156.01, 148.05, 134.44, 132.46, 132.40, 132.24, 129.07, 127.15, 123.53, 121.88, 118.34, 117.21, 57.94.

HRMS $[C_{16}H_{12}ClN_3O_4 + H]^+$: 346.05891 calculated, 346.05886 found.

_N_-(2-Cyano-4-(5-methoxy-2-oxo-1,3,4-oxadiazol-3(2_H_)-yl)phenyl)benzamide (21)

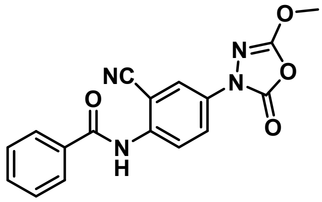

67e (6.0 mg, 26 µmol) and benzoyl chloride (6.6 µL, 57 µmol) were reacted following General Procedure A, with the addition that a catalytic amount of DMAP was used. Purification of the crude material by column chromatography (0% → 0.25% MeOH in DCM) gave the final compound as a white solid (6.8 mg, 20 µmol, 78%).

^{1}H NMR (400 MHz, $CDCl_3$) δ 8.73 – 8.65 (m, 1H), 8.41 (s, 1H), 8.15 – 8.06 (m, 2H), 7.98 – 7.89 (m, 2H), 7.67 – 7.59 (m, 1H), 7.59 – 7.47 (m, 2H), 4.15 (s, 3H).

^{13}C NMR (101 MHz, $CDCl_3$) δ 165.47, 156.18, 147.95, 137.89, 133.53, 132.87, 132.34, 129.22, 127.27, 123.31, 122.12, 120.63, 115.81, 102.80, 58.08.

HRMS $[C_{17}H_{12}N_4O_4 + H]^+$: 337.09313 calculated, 337.09304 found.

_N_-(4-(5-Methoxy-2-oxo-1,3,4-oxadiazol-3(2_H_)-yl)-2-methylphenyl)nicotinamide (22)

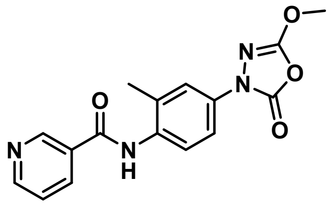

12 (20 mg, 90 µmol) and nicotinic acid (16.7 mg, 0.136 mmol) were reacted following General Procedure C. The resulting crude material was purified by column chromatography (0.1% → 0.5%

MeOH in DCM) to obtain **22** as a yellow solid (13 mg, 0.40 mmol, 44%).

^{1}H NMR (400 MHz, $CDCl_3$) δ 9.14 – 9.07 (m, 1H), 8.77 (dd, *J* = 4.9, 1.7 Hz, 1H), 8.22 (dt, *J* = 8.0, 2.0 Hz, 1H), 7.92 (s, 1H), 7.86 (d, *J* = 8.8 Hz, 1H), 7.69 (d, *J* = 2.5 Hz, 1H), 7.64 (dd, *J* = 8.7, 2.6 Hz, 1H), 7.44 (dd, *J* = 8.0, 4.9 Hz, 1H), 4.11 (s, 3H), 2.35 (s, 3H).

^{13}C NMR (101 MHz, $CDCl_3$) δ 164.07, 155.97, 152.81, 148.35, 148.03, 135.56, 133.77, 132.77, 131.47, 130.54, 125.15, 123.89, 119.92, 116.49, 57.91, 18.23.

HRMS $[C_{16}H_{14}N_4O_4 + H]^+$: 327.10878 calculated, 327.10855 found.

***N*-(4-(5-Methoxy-2-oxo-1,3,4-oxadiazol-3(2*H*)-yl)-2-methylphenyl)picolinamide (23)**

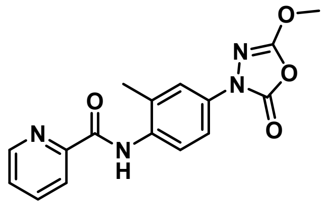

12 (20 mg, 90 µmol) and picolinic acid (18.6 mg, 0.151 mmol) were reacted following General Procedure B. The resulting crude material was purified by column chromatography (0.1% → 2% MeOH in DCM) to obtain **23** as a yellow solid (12 mg, 38 µmol, 42%).

^{1}H NMR (300 MHz, DMSO) δ 10.33 (s, 1H), 8.75 (ddd, *J* = 4.8, 1.7, 0.9 Hz, 1H), 8.17 (dt, *J* = 7.8, 1.2 Hz, 1H), 8.08 (td, *J* = 7.6, 1.7 Hz, 1H), 7.90 (d, *J* = 8.6 Hz, 1H), 7.70 (ddd, *J* = 7.5, 4.8, 1.4 Hz, 1H), 7.63 – 7.54 (m, 2H), 4.08 (s, 3H), 2.35 (s, 3H).

^{13}C NMR (75 MHz, DMSO) δ 162.15, 154.73, 149.55, 148.63, 147.36, 138.29, 133.41, 132.81, 131.93, 127.11, 124.19, 122.25, 119.49, 115.81, 58.15, 17.81.

HRMS $[C_{16}H_{14}N_4O_4 + H]^+$: 327.10878 calculated, 327.10853 found.

***N*-(4-(5-Methoxy-2-oxo-1,3,4-oxadiazol-3(2*H*)-yl)-2-methylphenyl)pyrimidine-5-carboxamide (24)**

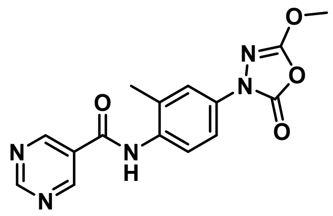

12 (20 mg, 90 µmol) and pyrazine-2-carbonyl chloride (14 mg, 99 µmol) were reacted following General Procedure A. The resulting crude material was purified by column chromatography (1% → 10% MeOH in DCM) to obtain **24** as a yellow solid (18 mg, 55 µmol, 61%).

^{1}H NMR (400 MHz, DMSO) δ 10.27 (s, 1H), 9.36 (s, 1H), 9.27 (s, 2H), 7.64 – 7.54 (m, 2H), 7.49 (d, *J* = 8.6 Hz, 1H), 4.07 (s, 3H), 2.29 (s, 3H).

^{13}C NMR (101 MHz, DMSO) δ 162.32, 159.95, 156.29, 155.45, 148.04, 134.77, 133.98, 132.82, 128.07, 127.17, 119.33, 115.53, 58.17, 18.16.

HRMS $[C_{15}H_{13}N_5O_4 + H]^+$: 328.10403 calculated, 328.10399 found.

***N*-(4-(5-Methoxy-2-oxo-1,3,4-oxadiazol-3(2*H*)-yl)-2-methylphenyl)pyrazine-2-carboxamide (25)**

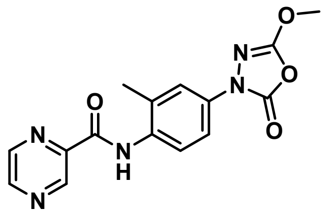

Pyrazinoic acid (18.6 mg, 0.151 mmol) and **12** (20 mg, 90 µmol) were reacted following General Procedure B. The resulting crude material was purified by column chromatography (0.1% → 0.5% MeOH in DCM) to obtain **25** as a yellow solid (18 mg, 55 µmol, 61%).

^{1}H NMR (400 MHz, $CDCl_3$) δ 9.96 (s, 1H), 9.33 (d, *J* = 1.4 Hz, 1H), 9.06 (d, *J* = 5.0 Hz, 1H), 8.34 (d, *J* = 8.9 Hz, 1H), 8.24 (dd, *J* = 5.0, 1.4 Hz, 1H), 7.74 (d, *J* = 2.6 Hz, 1H), 7.69 (dd, *J* = 8.9, 2.6 Hz, 1H), 4.12 (s, 3H), 2.47 (s, 3H).

^{13}C NMR (101 MHz, $CDCl_3$) δ 160.24, 159.81, 157.91, 156.49, 155.99, 148.38, 133.09, 132.83, 129.37, 122.11, 119.97, 118.74, 116.73, 57.89, 18.01.

HRMS $[C_{19}H_{15}N_3O_4 + H]^+$: 328.10403 calculated, 328.10380 found.

6-Chloro-*N*-(4-(5-methoxy-2-oxo-1,3,4-oxadiazol-3(2*H*)-yl)-2-methylphenyl)nicotinamide (26)

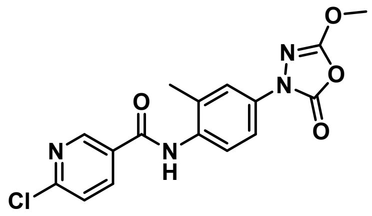

12 (20 mg, 90 µmol) and 6-chloronicotinoyl chloride (14 mg, 99 µmol) were reacted following General Procedure A. The resulting crude material was purified by column chromatography (1% → 10% MeOH in DCM) to obtain **26** as a yellow solid (11 mg, 30 µmol, 33%).

^{1}H NMR (400 MHz, DMSO) δ 10.20 (s, 1H), 8.97 (d, *J* = 2.6 Hz, 1H), 8.36 (dd, *J* = 8.3, 2.5 Hz, 1H), 7.72 (dd, *J* = 8.3, 0.7 Hz, 1H), 7.65 – 7.55 (m, 2H), 7.51 – 7.44 (m, 1H), 4.08 (s, 3H), 2.29 (s, 3H).

^{13}C NMR (101 MHz, DMSO) δ 163.00, 155.44, 152.89, 149.34, 148.03, 139.06, 134.89, 133.94, 133.03, 129.43, 127.34, 124.26, 119.30, 115.52, 58.16, 18.15.

HRMS $[C_{16}H_{13}ClN_4O_4 + H]^+$: 361.06981 calculated, 361.06955 found.

4-Chloro-*N*-(4-(5-methoxy-2-oxo-1,3,4-oxadiazol-3(2*H*)-yl)-2-methylphenyl)benzamide (27)

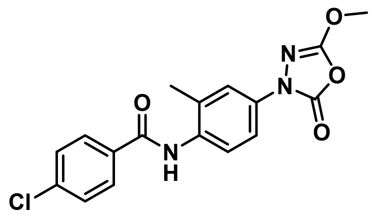

12 (20 mg, 90 µmol) was reacted with 4-chlorobenzoyl chloride (13 µL, 99 µmol) according to General Procedure A. Purification of the crude material by column chromatography (0% → 2% MeOH in DCM) gave the final compound (29 mg, 81 µmol, 89%).

^{1}H NMR (400 MHz, $CDCl_3$) δ 7.81 (m, 4H), 7.66 (d, *J* = 2.5 Hz, 1H), 7.60 (dd, *J* = 8.8, 2.5 Hz, 1H), 7.44 (d, *J* = 8.6 Hz, 2H), 4.12 (s, 3H), 2.32 (s, 3H).

^{13}C NMR (101 MHz, $CDCl_3$) δ 164.93, 155.93, 148.33, 138.34, 133.51, 133.05, 133.03, 131.32, 129.16, 128.68, 124.44, 119.82, 116.40, 57.89, 18.18.

HRMS $[C_{17}H_{14}ClN_3O_4 + H]^+$: 360.07456 calculated, 360.07431 found.

4-Methoxy-*N*-(4-(5-methoxy-2-oxo-1,3,4-oxadiazol-3(2*H*)-yl)-2-methylphenyl)benzamide (28)

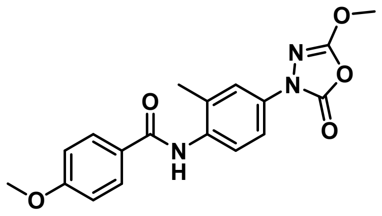

12 (20 mg, 90 µmol) was reacted with 4-methoxybenzoyl chloride (17 mg, 99 µmol) according to General Procedure A. Purification of the crude material by column chromatography (0% → 2% MeOH in DCM) gave the title compound (26 mg, 73 µmol, 81%).

^{1}H NMR (400 MHz, $CDCl_3$) δ 7.96 (d, *J* = 8.8 Hz, 1H), 7.85 (dd, *J* = 9.0, 2.3 Hz, 2H), 7.76 – 7.65 (m, 2H), 7.62 (dd, *J* = 8.8, 2.6 Hz, 1H), 7.03 – 6.94 (m, 2H), 4.11 (s, 3H), 3.88 (s, 3H), 2.35 (s, 3H).

^{13}C NMR (101 MHz, $CDCl_3$) δ 165.35, 162.69, 155.91, 148.37, 133.62, 133.04, 130.57, 129.07, 126.97, 123.95, 119.84, 116.53, 114.14, 57.86, 55.62, 18.19.

HRMS $[C_{18}H_{17}N_3O_5 + H]^+$: 356.12410 calculated, 356.12390 found.

***N*-(4-(5-Methoxy-2-oxo-1,3,4-oxadiazol-3(2*H*)-yl)-2-methylphenyl)-4-methylbenzamide (29)**

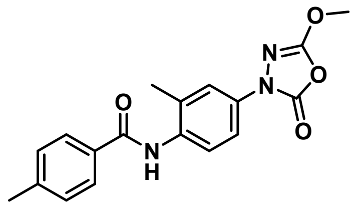

12 (20 mg, 90 µmol) was reacted with 4-methylbenzoyl chloride (13 µL, 99 µmol) according to General Procedure A. Purification of the crude material by column chromatography (0% → 2% MeOH in DCM) gave the title compound (27 mg, 80 µmol, 88%).

^{1}H NMR (400 MHz, $CDCl_3$) δ 7.96 (d, *J* = 8.9 Hz, 1H), 7.78 (d, *J* = 8.4 Hz, 2H), 7.74 (s, 1H), 7.68 (d, *J* = 2.6 Hz, 1H), 7.62 (dd, *J* = 8.8, 2.6 Hz, 1H), 7.29 (d, *J* = 7.9 Hz, 2H), 4.11 (s, 3H), 2.43 (s, 3H), 2.35 (s, 3H).

^{13}C NMR (101 MHz, $CDCl_3$) δ 165.78, 155.91, 148.35, 142.66, 133.52, 133.09, 131.94, 130.59, 129.62, 127.20, 123.94, 119.83, 116.50, 57.86, 21.64, 18.17.

HRMS $[C_{18}H_{17}N_3O_4 + H]^+$: 340.12918 calculated, 340.12896 found.

4-Fluoro-*N*-(4-(5-methoxy-2-oxo-1,3,4-oxadiazol-3(2*H*)-yl)-2-methylphenyl)benzamide (30)

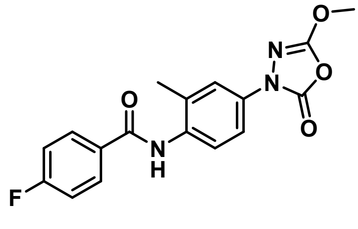

12 (20 mg, 90 µmol) was reacted with 4-fluorobenzoyl chloride (12 µL, 99 µmol) according to General Procedure A. Purification of the crude material by column chromatography (0% → 2% MeOH in DCM) gave the title compound (24 mg, 70 µmol, 77%).

^{1}H NMR (400 MHz, $CDCl_3$) δ 7.99 – 7.86 (m, 3H), 7.71 (d, *J* = 2.7 Hz, 1H), 7.65 (dd, *J* = 8.8, 2.6 Hz, 2H), 7.23 – 7.13 (m, 2H), 4.12 (s, 3H), 2.37 (s, 3H).

^{13}C NMR (101 MHz, $CDCl_3$) δ 165.14 (d, *J* = 253 Hz), 164.80, 155.97, 148.38, 133.41, 133.23, 130.99, 130.78, 129.60 (d, *J* = 9.2 Hz), 124.12, 119.92, 116.61, 116.11 (d, *J* = 22.2 Hz), 57.88, 18.21.

HRMS $[C_{17}H_{14}FN_3O_4 + H]^+$: 344.10411 calculated, 344.10395 found.

3-Chloro-*N*-(4-(5-methoxy-2-oxo-1,3,4-oxadiazol-3(2*H*)-yl)-2-methylphenyl)benzamide (31)

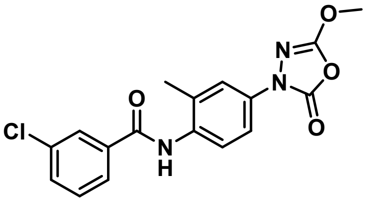

12 (20 mg, 90 µmol) was reacted with 3-chlorobenzoyl chloride (13 µL, 99 µmol) according to General Procedure A. Purification of the crude material by column chromatography (0% → 2% MeOH in DCM) gave the title compound (30 mg, 83 µmol, 92%).

^{1}H NMR (400 MHz, $CDCl_3$) δ 7.92 – 7.83 (m, 2H), 7.80 (d, *J* = 8.8 Hz, 1H), 7.74 (dt, *J* = 7.8, 1.4 Hz, 1H), 7.66 (d, *J* = 2.5 Hz, 1H), 7.60 (dd, *J* = 8.8, 2.6 Hz, 1H), 7.55 – 7.49 (m, 1H), 7.41 (t, *J* = 7.9 Hz, 1H), 4.12 (s, 3H), 2.33 (s, 3H).

^{13}C NMR (101 MHz, $CDCl_3$) δ 164.69, 155.93, 148.33, 136.48, 135.12, 133.61, 132.88, 132.10, 131.52, 130.23, 127.67, 125.25, 124.56, 119.82, 116.38, 57.90, 18.21.

HRMS $[C_{17}H_{14}ClN_3O_4 + H]^+$: 360.07456 calculated, 360.07426 found.

3-Methoxy-*N*-(4-(5-methoxy-2-oxo-1,3,4-oxadiazol-3(2*H*)-yl)-2-methylphenyl)benzamide (32)

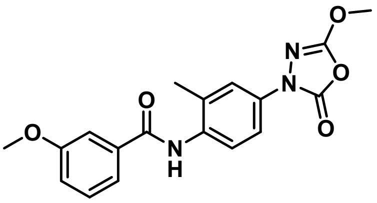

12 (20 mg, 90 µmol) and 3-methoxybenzoic acid (27.5 mg, 0.181 mmol), were reacted following General Procedure D using DMF (2.5 mL) instead of acetonitrile as solvent. The resulting crude material was purified by column chromatography (DCM → 25% EtOAc in pentane) to obtain **32** as white solid (14.4 mg, 41 µmol, 45%).

^{1}H NMR (400 MHz, $CDCl_3$) δ 7.70 (d, *J* = 2.4 Hz, 1H), 7.68 – 7.57 (m, 2H), 7.54 – 7.46 (m, 2H), 7.45 – 7.37 (m, 1H), 7.17 – 7.08 (m, 1H), 4.14 (s, 3H), 3.88 (s, 3H), 2.37 (s, 3H).

^{13}C NMR (101 MHz, $CDCl_3$) δ 166.84, 159.70, 155.77, 148.43, 135.59, 133.72, 133.56, 133.05, 129.61, 125.82, 119.77, 119.21, 117.84, 116.04, 112.62, 57.68, 55.23, 17.85.

HRMS $[C_{18}H_{17}N_3O_5 + H]^+$: 356.12410 calculated, 356.12423 found.

***N*-(4-(5-Methoxy-2-oxo-1,3,4-oxadiazol-3(2*H*)-yl)-2-methylphenyl)-3-methylbenzamide (33)**

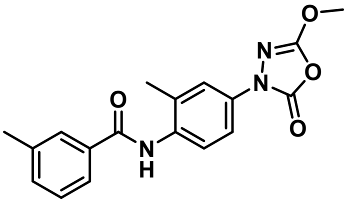

12 (20.0 mg, 90.0 µmol) and 3-methylbenzoic acid (18.5 mg, 0.136 mmol) were reacted following General Procedure C. The resulting crude material was purified by column chromatography (20% → 25% EtOAc in pentane) to obtain **33** as a white solid (10.9 mg, 32.0 µmol, 36%).

^{1}H NMR (400 MHz, $CDCl_3$) δ 7.99 (d, *J* = 9.0 Hz, 1H), 7.73 – 7.68 (m, 3H), 7.68 – 7.62 (m, 2H), 7.40 – 7.35 (m, 2H), 4.11 (s, 3H), 2.44 (s, 3H), 2.37 (s, 3H).

^{13}C NMR (101 MHz, $CDCl_3$) δ 166.02, 155.94, 148.38, 138.97, 134.86, 133.47, 133.19, 132.87, 130.57, 128.86, 128.00, 124.06, 123.94, 119.88, 116.59, 57.87, 21.56, 18.23.

HRMS $[C_{18}H_{17}N_3O_4 + H]^+$: 340.12918 calculated, 340.12912 found.

3,4-Dichloro-*N*-(4-(5-methoxy-2-oxo-1,3,4-oxadiazol-3(2*H*)-yl)-2-methylphenyl)benzamide (34)

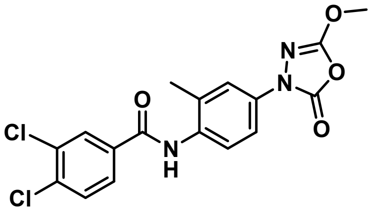

12 (20 mg, 90 µmol) was reacted with 3,4-dichlorobenzoyl chloride (21 mg, 99 µmol) according to General Procedure A. Purification of the crude material by column chromatography (0% → 2% MeOH in DCM) gave the title compound (23 mg, 58 µmol, 65%).

^1H NMR (400 MHz, DMSO) δ 10.13 (s, 1H), 8.22 (d, *J* = 2.1 Hz, 1H), 7.95 (dd, *J* = 8.4, 2.1 Hz, 1H), 7.83 (d, *J* = 8.3 Hz, 1H), 7.62 (d, *J* = 2.6 Hz, 1H), 7.57 (dd, *J* = 8.6, 2.6 Hz, 1H), 7.44 (d, *J* = 8.6 Hz, 1H), 4.08 (s, 3H), 2.27 (s, 3H).

^{13}C NMR (101 MHz, DMSO) δ 163.28, 155.46, 148.06, 135.03, 134.70, 134.48, 133.94, 133.23, 131.41, 130.88, 129.68, 128.02, 127.48, 119.32, 115.55, 58.18, 18.16.

HRMS $[C_{17}H_{13}Cl_2N_3O_4 + H]^+$: 394.03559 calculated, 394.03551 found.

2-Chloro-*N*-(4-(5-methoxy-2-oxo-1,3,4-oxadiazol-3(2*H*)-yl)-2-methylphenyl)benzamide (35)

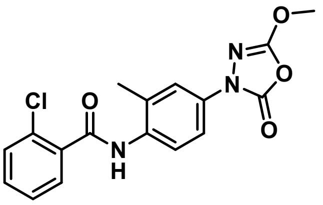

12 (20 mg, 90 µmol) was reacted with 2-chlorobenzoic acid (28.3 mg, 0.181 mmol) following General Procedure D, using DMF (2.5 mL) as solvent. The resulting crude material was purified by column chromatography (10% EtOAc in pentane → DCM) to obtain **35** as a white solid (7.0 mg, 19 µmol, 22%).

^1H NMR (400 MHz, $CDCl_3$) δ 8.09 (d, *J* = 8.8 Hz, 1H), 7.90 – 7.80 (m, 2H), 7.72 (d, *J* = 2.6 Hz, 1H), 7.67 (dd, *J* = 8.8, 2.6 Hz, 1H), 7.51 – 7.37 (m, 3H), 4.12 (s, 3H), 2.39 (s, 3H).

^{13}C NMR (101 MHz, $CDCl_3$) δ 164.53, 155.96, 148.37, 134.92, 133.41, 133.13, 132.04, 131.04, 130.63, 130.60, 130.56, 127.58, 123.90, 119.92, 116.55, 57.88, 18.49.

HRMS $[C_{17}H_{14}ClN_3O_4 + H]^+$: 360.07456 calculated, 360.07449 found.

2-Methoxy-*N*-(4-(5-methoxy-2-oxo-1,3,4-oxadiazol-3(2*H*)-yl)-2-methylphenyl)benzamide (36)

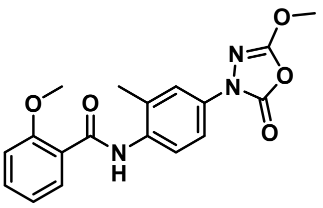

12 (20.0 mg, 90.0 µmol) and 2-methoxybenzoic acid (20.6 mg, 0.136 mmol), were reacted following General Procedure C. The resulting crude material was purified by column chromatography

(0.5% → 1% MeOH in DCM) to obtain **36** as a white solid (7.00 mg, 20.0 µmol, 22%).

^{1}H NMR (400 MHz, $CDCl_3$) δ 9.88 (s, 1H), 8.41 (d, *J* = 8.9 Hz, 1H), 8.33 (dd, *J* = 7.9, 1.9 Hz, 1H), 7.70 (d, *J* = 2.6 Hz, 1H), 7.63 (dd, *J* = 8.9, 2.6 Hz, 1H), 7.52 (ddd, *J* = 8.3, 7.3, 1.9 Hz, 1H), 7.16 (ddd, *J* = 8.1, 7.4, 1.1 Hz, 1H), 7.06 (dd, *J* = 8.5, 1.0 Hz, 1H), 4.11 (s, 3H), 4.08 (s, 3H), 2.40 (s, 3H).

^{13}C NMR (101 MHz, $CDCl_3$) δ 163.18, 157.31, 155.91, 148.43, 134.77, 133.46, 132.83, 132.13, 128.42, 122.53, 121.89, 121.79, 119.77, 116.72, 111.56, 57.84, 56.34, 18.40.

HRMS $[C_{18}H_{17}N_3O_5 + H]^+$: 356.12412 calculated, 356.12410.

_N_-(4-(5-Methoxy-2-oxo-1,3,4-oxadiazol-3(2*H*)-yl)-2-methylphenyl)-2-methylbenzamide (37)

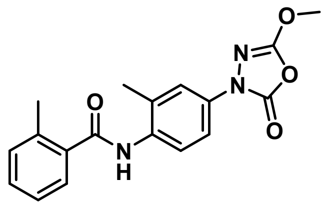

12 (20 mg, 90 µmol) was reacted with 2-methylbenzoic acid (25 mg, 0.181 mmol) following General Procedure D using DMF (2.5 mL) instead of acetonitrile as solvent. The resulting crude material was purified by column chromatography (DCM → 25% EtOAc in pentane) to obtain **37** as a white solid (6.0 mg, 18 µmol, 20%).

^{1}H NMR (400 MHz, MeOD) δ 7.70 (d, *J* = 2.3 Hz, 1H), 7.68 – 7.60 (m, 2H), 7.57 – 7.50 (m, 1H), 7.41 – 7.32 (m, 1H), 7.28 (d, *J* = 7.5 Hz, 2H), 4.14 (s, 3H), 2.51 (s, 3H), 2.37 (s, 3H).

^{13}C NMR (101 MHz, MeOD) δ 169.86, 155.71, 148.36, 135.91, 135.78, 133.73, 133.49, 132.78, 130.72, 129.95, 126.69, 125.82, 125.52, 119.69, 115.87, 57.54, 19.22, 17.80.

HRMS $[C_{18}H_{17}N_3O_4 + H]^+$: 340.12918 calculated, 340.12904 found.

4-Ethynyl-*N*-(4-(5-methoxy-2-oxo-1,3,4-oxadiazol-3(2*H*)-yl)-2-methylphenyl)benzamide (38)

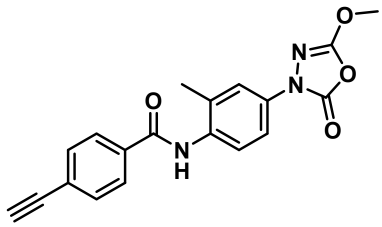

12 (20 mg, 90 µmol) and 4-ethynylbenzoic acid (22.0 mg, 0.151 mmol) were reacted following General Procedure B. Due to accidental addition of DMF (1 mL) work-up was slightly modified: brine was added and the mixture was extracted with Et_2O (3×). The organic layers were combined and washed with 10% aq. $NaHCO_3$, dried ($MgSO_4$), filtered and concentrated *in vacuo*. The resulting crude material was purified by column chromatography (5% → 40% EtOAc in pentane) to obtain **38** as a yellow solid (7.1 mg, 20 µmol, 22%).

^{1}H NMR (400 MHz, $CDCl_3$) δ 7.95 – 7.80 (m, 4H), 7.69 (d, *J* = 2.5 Hz, 1H), 7.63 (dd, *J* = 8.8, 2.6 Hz, 1H), 7.61 – 7.56 (m, 2H), 4.11 (s, 3H), 3.23 (s, 1H), 2.35 (s, 3H).

^{13}C NMR (101 MHz, $CDCl_3$) δ 165.04, 155.97, 148.37, 134.74, 133.43, 133.16, 132.69, 130.74, 127.19, 126.05, 124.07, 119.92, 116.61, 82.71, 80.09, 57.88, 18.21.

HRMS [$C_{19}H_{15}N_3O_4$ + H]$^+$: 350.11353 calculated, 350.11332 found.

***N*-(4-(5-Methoxy-2-oxo-1,3,4-oxadiazol-3(2*H*)-yl)-2-methylphenyl)-1-naphthamide (39)**

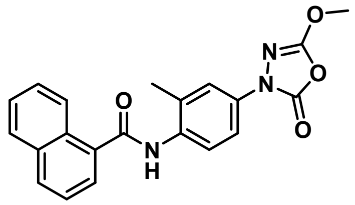

12 (20 mg, 90 µmol) and 1-naphthoic acid (23.4 mg, 0.136 mmol) were reacted following General Procedure C. The crude product was purified with preparative HPLC to obtain **39** as a white solid (19.6 mg, 52.0 µmol, 58%).

^{1}H NMR (400 MHz, DMSO) δ 10.12 (s, 1H), 8.29 (d, *J* = 8.0 Hz, 1H), 8.08 (d, *J* = 8.2 Hz, 1H), 8.05 – 7.98 (m, 1H), 7.83 (d, *J* = 7.0 Hz, 1H), 7.66 – 7.56 (m, 6H), 4.09 (s, 3H), 2.38 (s, 3H).

^{13}C NMR (101 MHz, DMSO) δ 174.47, 167.63, 155.50, 148.12, 134.52, 133.67, 133.27, 130.24, 129.89, 129.73, 128.41, 127.18, 127.05, 126.44, 125.67, 125.29, 125.14, 119.45, 115.63, 58.22, 18.37.

HRMS [$C_{21}H_{17}N_3O_4$ + H]$^+$: 376.12918 calculated, 376.12926.

***N*-(4-(5-Methoxy-2-oxo-1,3,4-oxadiazol-3(2*H*)-yl)-2-methylphenyl)-2-naphthamide (40)**

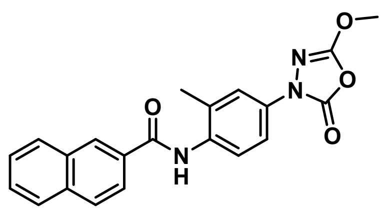

12 (20 mg, 90 µmol) and 2-naphthoic acid (23.4 mg, 0.136 mmol were reacted following General Procedure C. The resulting crude material was purified by column chromatography (10% → 15% EtOAc in pentane) to obtain **40** as a white solid (10 mg, 27 µmol, 30%).

^{1}H NMR (400 MHz, $CDCl_3$) δ 8.40 (s, 1H), 8.03 (d, *J* = 8.8 Hz, 1H), 7.98 – 7.83 (m, 5H), 7.72 (dd, *J* = 2.5, Hz, 1H), 7.66 (dd, *J* = 8.8, 2.6 Hz, 1H), 7.63 – 7.53 (m, 2H), 4.12 (s, 3H), 2.41 (s, 3H).

^{13}C NMR (101 MHz, $CDCl_3$) δ 165.93, 155.95, 148.38, 135.05, 133.47, 133.29, 132.76, 132.03, 130.72, 129.15, 128.98, 128.13, 127.97, 127.82, 127.15, 124.07, 123.61, 119.91, 116.60, 57.87, 18.28.

HRMS [$C_{21}H_{17}N_3O_4$ + H]$^+$: 376.12918 calculated, 376.12938 found.

***N*-(4-(5-Methoxy-2-oxo-1,3,4-oxadiazol-3(2*H*)-yl)-2-methylphenyl)-[1,1'-biphenyl]-3-carboxamide (41)**

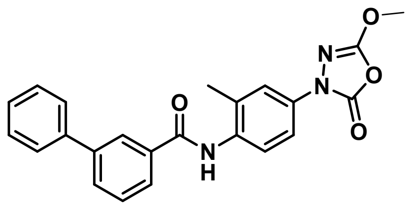

In a microwave tube, **73** (20 mg, 49 µmol), phenylboronic acid (7.24 mg, 32 µmol) and K_2CO_3 (27.4 mg, 0.198 mmol) were dissolved in 1,4-dioxane (0.4 mL) and H_2O (0.133 mL). The mixture was flushed with nitrogen gas using a sonicator, followed by addition of $Pd(PPh_3)_4$ (0.86 mg, 0.74 µmol). Subsequently the tube was capped and the mixture was stirred at 40°C for 28 h, after which the reaction mixture was filtered over Celite®, flushed with EtOAc and concentrated *in vacuo*. The resulting crude material was

purified by column chromatography (15% → 30% EtOAc in pentane) to obtain **41** as a white solid (13 mg, 32 µmol, 64%).

^{1}H NMR (400 MHz, $CDCl_3$) δ 8.11 (t, *J* = 1.8 Hz, 1H), 8.01 (d, *J* = 8.9 Hz, 1H), 7.84 (dt, *J* = 7.8, 1.4 Hz, 1H), 7.79 (ddd, *J* = 7.7, 1.9, 1.1 Hz, 1H), 7.76 (s, 1H), 7.73 – 7.70 (m, 1H), 7.69 – 7.61 (m, 3H), 7.57 (t, *J* = 7.7 Hz, 1H), 7.51 – 7.45 (m, 2H), 7.43 – 7.37 (m, 1H), 4.12 (s, 3H), 2.38 (s, 3H).

^{13}C NMR (101 MHz, $CDCl_3$) δ 165.84, 155.97, 148.39, 142.23, 140.19, 135.50, 133.39, 133.32, 130.82, 130.68, 129.46, 129.13, 128.07, 127.36, 126.17, 125.74, 124.03, 119.92, 116.62, 57.87, 18.26.

HRMS $[C_{23}H_{19}N_3O_4 + H]^+$: 402.14483 calculated, 402.14497 found.

N-(4-(5-Methoxy-2-oxo-1,3,4-oxadiazol-3(2H)-yl)-2-methylphenyl)-3-(pyrimidin-5-yl)benzamide (42)

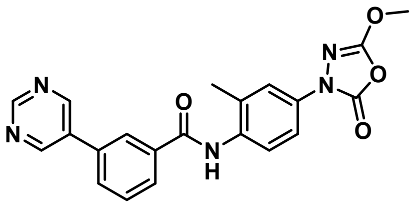

In a microwave tube, **73** (60 mg, 0.15 mmol), pyrimidin-5-ylboronic acid (31 mg, 0.25 mmol) and K_2CO_3 (82 mg, 0.59 mmol) were dissolved in 1,4-dioxane (1.2 mL) and H_2O (0.40 mL). The mixture was flushed with nitrogen gas using a sonicator, followed by addition of $Pd(PPh_3)_4$ (2.6 mg, 2.2 µmol). Subsequently the tube was capped and the reaction mixture was stirred at 45°C overnight. Additional pyrimidin-5-ylboronic acid (16 mg, 0.10 mmol) and $Pd(PPh_3)_4$ (1.3 mg, 1.1 µmol) were added and the reaction mixture was stirred at 45°C for over the weekend. The reaction mixture was filtered over a glass filter, flushed with EtOAc, and the filtrate was concentrated *in vacuo*. The resulting crude material was purified by column chromatography (0% → 3% MeOH in DCM) to obtain **42** as a white solid (15 mg, 38 µmol, 26%).

^{1}H NMR (400 MHz, MeOD) δ 9.18 (s, 1H), 9.07 (s, 2H), 8.26 (t, *J* = 1.9 Hz, 1H), 8.08 (d, *J* = 7.8 Hz, 1H), 7.87 – 7.80 (m, 1H), 7.72 – 7.61 (m, 3H), 7.53 – 7.47 (m, 2H), 4.14 (s, 3H), 2.36 (s, 3H).

^{13}C NMR (101 MHz, MeOD) δ 166.66, 157.08, 155.95, 155.05, 148.57, 135.55, 134.92, 134.32, 134.23, 134.05, 133.05, 130.22, 129.85, 128.27, 126.86, 126.39, 119.88, 116.02, 57.81, 18.01.

HRMS $[C_{21}H_{17}N_5O_4 + H]^+$: 404.13533 calculated, 404.13516 found.

4'-Chloro-N-(4-(5-methoxy-2-oxo-1,3,4-oxadiazol-3(2H)-yl)-2-methylphenyl)-[1,1'-biphenyl]-3-carboxamide (43)

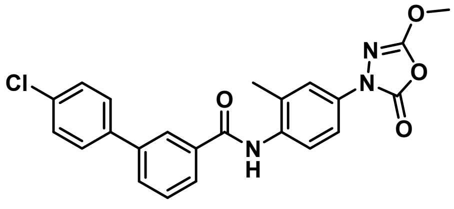

In a microwave tube, **73** (60.0 mg, 0.148 mmol), (4-chlorophenyl)boronic acid (27.9 mg, 0.178 mmol) and K_2CO_3 (82.0 mg, 0.594 mmol) were dissolved in 1,4-dioxane (0.4 mL) and H_2O (0.13 mL). The mixture was flushed with nitrogen gas using a sonicator, followed by addition of $Pd(PPh_3)_4$ (2.57 mg, 2.23 µmol). Subsequently the tube was capped and the

reaction mixture was stirred at 45°C over the weekend, after which it was filtered over a glass filter, flushed with EtOAc and concentrated *in vacuo*. The resulting crude material was purified by column chromatography (10% → 30% EtOAc in pentane) to obtain **43** as a white solid (39.7 mg, 91.0 µmol, 61%).

^{1}H NMR (400 MHz, $CDCl_3$) δ 8.09 (t, *J* = 1.8 Hz, 1H), 7.99 (d, *J* = 8.8 Hz, 1H), 7.83 (dt, *J* = 7.7, 1.5 Hz, 1H), 7.77 – 7.73 (m, 2H), 7.72 (d, *J* = 2.6 Hz, 1H), 7.66 (dd, *J* = 8.8, 2.6 Hz, 1H), 7.60 – 7.53 (m, 3H), 7.47 – 7.41 (m, 2H), 4.12 (s, 3H), 2.38 (s, 3H).

^{13}C NMR (101 MHz, $CDCl_3$) δ 165.68, 155.97, 148.38, 141.02, 138.60, 135.61, 134.26, 133.40, 133.28, 130.76, 130.59, 129.55, 129.29, 128.59, 126.17, 125.86, 124.09, 119.92, 116.62, 57.88, 18.26.

HRMS $[C_{23}H_{18}ClN_3O_4 + H]^+$: 436.10586 calculated, 436.10587 found.

***N*-(4-(5-Methoxy-2-oxo-1,3,4-oxadiazol-3(2*H*)-yl)-2-methylphenyl)-3-(phenylethynyl)benzamide (44)**

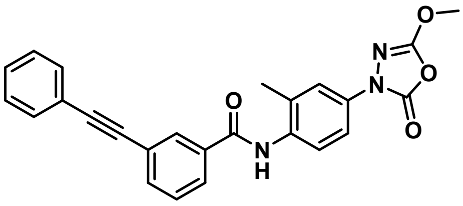

3-(Phenylethynyl)benzoic acid (**77**) (75 mg, 0.34 mmol) was reacted with **12** (38 mg, 0.17 mmol) following General Procedure D. The resulting crude material was purified by column chromatography (20% → 30% EtOAc in pentane) to obtain **44** as a white solid (51 mg, 0.12 mmol, 71%).

^{1}H NMR (400 MHz, $CDCl_3$) δ 8.02 (t, *J* = 1.7 Hz, 1H), 7.94 (d, *J* = 8.8 Hz, 1H), 7.85 (d, *J* = 7.9 Hz, 1H), 7.80 – 7.68 (m, 3H), 7.65 (dd, *J* = 8.8, 2.6 Hz, 1H), 7.57 – 7.53 (m, 2H), 7.50 (t, *J* = 7.8 Hz, 1H), 7.41 – 7.33 (m, 3H), 4.11 (s, 3H), 2.38 (s, 3H).

^{13}C NMR (101 MHz, $CDCl_3$) δ 165.18, 155.94, 148.36, 135.11, 134.94, 133.47, 133.14, 131.82, 131.06, 130.13, 129.15, 128.82, 128.73, 128.58, 127.06, 124.29, 122.83, 119.89, 116.54, 90.83, 88.33, 57.87, 18.28.

HRMS $[C_{25}H_{19}N_3O_4 + H]^+$: 426.14483 calculated, 426.14495 found.

***N*-(4-(5-Methoxy-2-oxo-1,3,4-oxadiazol-3(2*H*)-yl)-2-methylphenyl)-3-(pyridin-3-yloxy)benzamide (45)**

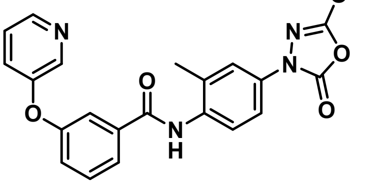

80 (58 mg, 0.27 mmol) was reacted with **12** (30 mg, 0.135 mmol) following General Procedure D using 5 equivalents of DIPEA. The resulting crude material was purified by column chromatography (0.1% → 1.2% MeOH in DCM) to obtain **45** as a white solid (5.0 mg, 12 µmol, 9%).

^{1}H NMR (500 MHz, $CDCl_3$) δ 8.45 – 8.37 (m, 2H), 7.90 – 7.84 (m, 2H), 7.73 – 7.61 (m, 3H), 7.56 (m, 1H), 7.49 (t, *J* = 7.9 Hz, 1H), 7.39 – 7.28 (m, 2H), 7.21 (ddd, *J* = 8.2, 2.6, 0.9 Hz, 1H), 4.12 (s, 3H), 2.35 (s, 3H).

^{13}C NMR (126 MHz, $CDCl_3$) δ 165.05, 157.26, 155.95, 153.31, 148.37, 145.17, 141.81, 136.94, 133.55, 133.10, 131.30, 130.68, 126.24, 124.48, 124.40, 122.24, 122.20, 119.93, 117.79, 116.51, 57.91, 18.18.

HRMS $[C_{22}H_{18}N_4O_5 + H]^+$: 419.13500 calculated, 419.13459 found.

N-(4-(5-Methoxy-2-oxo-1,3,4-oxadiazol-3(2*H*)-yl)-2-methylphenyl)-3-(pyridin-2-yloxy)benzamide (46)

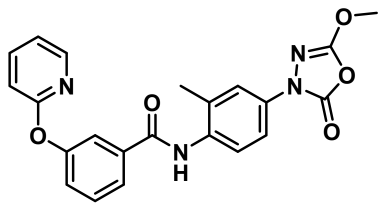

83 (58 mg, 0.27 mmol) was reacted with **12** (30 mg, 0.135 mmol) following General Procedure D using 5 equivalents of DIPEA. The resulting crude material was purified by column chromatography (0.5% → 1% MeOH in DCM) to obtain **46** as a yellow solid (6.0 mg, 14 µmol, 11%).

^{1}H NMR (400 MHz, MeOD) δ 8.16 (ddd, *J* = 5.1, 2.0, 0.8 Hz, 1H), 7.93 – 7.82 (m, 2H), 7.73 (d, *J* = 2.4 Hz, 2H), 7.67 (dd, *J* = 8.6, 2.6 Hz, 1H), 7.58 (t, *J* = 8.0 Hz, 1H), 7.41 (d, *J* = 8.7 Hz, 1H), 7.35 (ddd, *J* = 8.2, 2.4, 1.0 Hz, 1H), 7.18 – 7.13 (m, 1H), 7.04 (d, *J* = 8.3, 1H), 4.13 (s, 3H), 2.34 (s, 3H).

^{13}C NMR (101 MHz, MeOD) δ 168.22, 164.72, 157.54, 155.99, 149.95, 148.44, 141.77, 137.35, 136.80, 136.21, 134.35, 131.22, 128.52, 125.67, 124.91, 121.37, 120.82, 120.54, 116.98, 113.19, 58.63, 18.46.

HRMS $[C_{22}H_{18}N_4O_5 + H]^+$: 419.13500 calculated, 419.13481 found.

3-(4-Chlorophenoxy)-*N*-(4-(5-methoxy-2-oxo-1,3,4-oxadiazol-3(2*H*)-yl)-2-methylphenyl)benzamide (47)

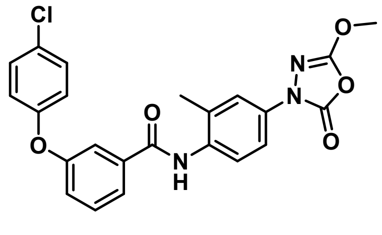

86b (67 mg, 0.27 mmol) was reacted with **12** (30 mg, 0.135 mmol) following General Procedure D. The resulting crude material was purified by column chromatography (10% → 50% Et_2O in pentane) to obtain **47** as a yellow solid (35 mg, 77 µmol, 57%).

^{1}H NMR (400 MHz, $CDCl_3$) δ 7.85 (s, 1H), 7.81 (d, *J* = 8.8 Hz, 1H), 7.63 (d, *J* = 2.5 Hz, 1H), 7.61 – 7.54 (m, 2H), 7.54 – 7.48 (m, 1H), 7.42 (t, *J* = 7.9 Hz, 1H), 7.34 – 7.27 (m, 2H), 7.15 – 7.10 (m, 1H), 7.00 – 6.91 (m, 2H), 4.09 (s, 3H), 2.30 (s, 3H).

^{13}C NMR (101 MHz, $CDCl_3$) δ 165.10, 157.69, 155.87, 155.17, 148.28, 136.70, 133.40, 133.06, 130.39, 130.06, 129.12, 128.31, 125.38, 122.00, 121.80, 120.61, 117.60, 116.31, 57.86, 18.13.

HRMS $[C_{23}H_{18}ClN_3O_5 + H]^+$: 452.10077 calculated, 452.10059 found.

N-(4-(5-Methoxy-2-oxo-1,3,4-oxadiazol-3(2*H*)-yl)-2-methylphenyl)-3-(4-methoxyphenoxy)benzamide (48)

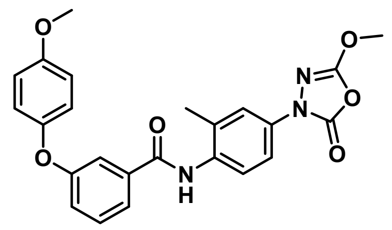

86d (67 mg, 0.27 mmol) was reacted with **12** (30 mg, 0.135 mmol) following General Procedure D. The crude product was purified by column chromatography (10% → 50% Et_2O in pentane) to obtain **48** as a white solid (11 mg, 23 µmol, 17%).

^{1}H NMR (400 MHz, $CDCl_3$) δ 8.00 (d, *J* = 9.0 Hz, 1H), 7.70 (d, *J* = 2.6 Hz, 1H), 7.64 (dd, *J* = 8.8, 2.6 Hz, 2H), 7.51 (dt, *J* = 7.9, 1.3 Hz, 1H), 7.48 – 7.37 (m, 2H), 7.13 (ddd, *J* = 8.2, 2.5, 1.0 Hz, 1H), 7.07 – 6.97 (m, 2H), 6.96 – 6.87 (m, 2H), 4.11 (s, 3H), 3.82 (s, 3H), 2.35 (s, 3H).

^{13}C NMR (101 MHz, $CDCl_3$) δ 165.30, 159.34, 156.48, 155.90, 149.33, 148.32, 136.52, 133.25, 130.72, 130.21, 123.96, 121.31, 121.26, 120.81, 120.66, 119.79, 116.44, 116.12, 115.18, 57.86, 55.77, 18.12.

HRMS $[C_{24}H_{21}N_3O_6 + H]^+$: 448.15031 calculated, 448.15003 found.

N-(4-(5-Methoxy-2-oxo-1,3,4-oxadiazol-3(2*H*)-yl)-2-methylphenyl)-3-(p-tolyloxy)benzamide (49)

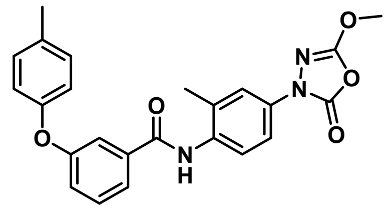

86c (62 mg, 0.27 mmol) was reacted with **12** (30 mg, 0.135 mmol) following General Procedure D. The resulting crude material was purified by column chromatography (10% → 50% Et_2O in pentane) to obtain **49** as a white solid (34 mg, 79 µmol, 58%).

^{1}H NMR (400 MHz, $CDCl_3$) δ 7.94 – 7.86 (m, 1H), 7.78 – 7.71 (m, 1H), 7.68 – 7.65 (m, 1H), 7.61 (dd, *J* = 9.0, 2.5 Hz, 1H), 7.53 (d, *J* = 7.8, 1H), 7.47 (t, *J* = 2.1 Hz, 1H), 7.40 (td, *J* = 7.9, 1.4 Hz, 1H), 7.20 – 7.11 (m, 3H), 6.97 – 6.91 (m, 2H), 4.10 (s, 3H), 2.34 (s, 3H), 2.31 (s, 3H).

^{13}C NMR (101 MHz, $CDCl_3$) δ 165.30, 158.71, 155.90, 153.92, 148.32, 136.54, 133.91, 133.27, 133.23, 130.79, 130.61, 130.52, 130.25, 124.02, 121.46, 121.03, 119.79, 119.68, 116.76, 116.43, 57.85, 20.86, 18.12.

HRMS $[C_{24}H_{21}N_3O_5 + H]^+$: 432.15540 calculated, 432.15520 found.

3-(4-Fluorophenoxy)-N-(4-(5-methoxy-2-oxo-1,3,4-oxadiazol-3(2*H*)-yl)-2-methylphenyl)benzamide (50)

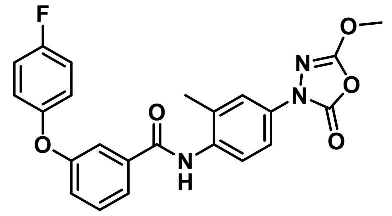

86e (63 mg, 0.27 mmol) was reacted with **12** (30 mg, 0.135 mmol) following General Procedure D. The crude product was purified by column chromatography (0% → 0.5% MeOH in DCM) to obtain **50** as a white solid (18 mg, 41 µmol, 31%).

^{1}H NMR (400 MHz, $CDCl_3$) δ 7.91 (d, *J* = 8.8 Hz, 1H), 7.74 (s, 1H), 7.70 – 7.65 (m, 1H), 7.62 (dd, *J* = 8.8, 2.6 Hz, 1H), 7.55 (dt, *J* = 7.7, 1.3 Hz, 1H), 7.48 (t, *J* = 2.1 Hz, 1H), 7.43 (t, *J* = 7.9 Hz, 1H), 7.14 (ddd, *J* = 8.2, 2.5, 1.0 Hz, 1H), 7.11 – 6.97 (m, 4H), 4.11 (s, 3H), 2.33 (s, 3H).

^{13}C NMR (101 MHz, $CDCl_3$) δ 165.14, 159.34 (d, *J* = 243 Hz), 158.56, 155.92, 152.12 (d, *J* = 2.6 Hz), 148.33, 136.71, 133.34, 133.17, 130.78, 130.39, 124.03, 121.42, 121.24, 121.19 (d, *J* = 8.2 Hz), 119.83, 116.88, 116.73 (d, *J* = 23 Hz), 116.47, 57.87, 18.15.

HRMS $[C_{23}H_{18}FN_3O_5 + H]^+$: 436.13033 calculated, 436.13014 found.

_N_-(4-(5-Methoxy-2-oxo-1,3,4-oxadiazol-3(2_H_)-yl)-2-methylphenyl)-3-(4-(trifluoromethyl)phenoxy)benzamide (51)

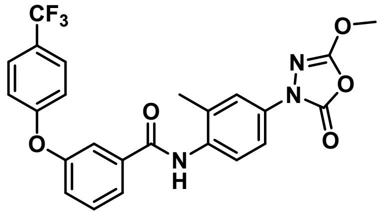

86a (77 mg, 0.27 mmol) was reacted with **12** (30 mg, 0.135 mmol) following General Procedure D. The resulting crude material was purified by column chromatography (10% → 50% Et_2O in pentane) to obtain **51** as a white solid (38 mg, 78 µmol, 58%).

^{1}H NMR (400 MHz, $CDCl_3$) δ 7.88 – 7.81 (m, 2H), 7.67 – 7.64 (m, 2H), 7.63 – 7.55 (m, 4H), 7.49 (t, *J* = 8.0 Hz, 1H), 7.22 (ddd, *J* = 8.2, 2.5, 1.0 Hz, 1H), 7.08 (d, *J* = 8.4 Hz, 2H), 4.11 (s, 3H), 2.32 (s, 3H).

^{13}C NMR (101 MHz, $CDCl_3$) δ 164.98, 159.77, 156.61, 155.92, 148.32, 136.93, 133.50, 133.01, 131.24, 130.63, 127.43 (q, *J* = 3.8 Hz), 125.70 (32 Hz), 124.34, 124.14 (q, *J* = 273 Hz), 123.07, 122.63, 119.81, 118.69, 118.54, 116.39, 57.87, 19.43.

HRMS $[C_{24}H_{18}F_3N_3O_5 + H]^+$: 486.12713 calculated, 486.12723 found.

3-(3-Chlorophenoxy)-_N_-(4-(5-methoxy-2-oxo-1,3,4-oxadiazol-3(2_H_)-yl)-2-methylphenyl)benzamide (52)

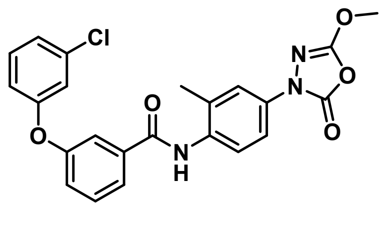

86f (67 mg, 0.27 mmol) was reacted with **12** (30 mg, 0.135 mmol) following General Procedure D. The crude product was purified by preparative HPLC to obtain **52** as a yellow oil (18 mg, 41 µmol, 31%).

^{1}H NMR (400 MHz, $CDCl_3$) δ 7.90 (d, *J* = 8.8 Hz, 1H), 7.75 (s, 1H), 7.69 (dd, *J* = 2.6, 0.8 Hz, 1H), 7.67 – 7.58 (m, 2H), 7.53 (s, 1H), 7.48 (t, *J* = 7.9 Hz, 1H), 7.28 (t, *J* = 8.1 Hz, 1H), 7.20 (ddd, *J* = 8.2, 2.5, 1.0 Hz, 1H), 7.12 (ddd, *J* = 8.0, 2.0, 0.9 Hz, 1H), 7.02 (t, *J* = 2.2 Hz, 1H), 6.92 (ddd, *J* = 8.2, 2.4, 0.9 Hz, 1H), 4.11 (s, 3H), 2.34 (s, 3H).

^{13}C NMR (101 MHz, $CDCl_3$) δ 165.61, 157.51, 157.33, 155.95, 148.37, 136.71, 135.40, 133.56, 132.97, 131.04, 130.89, 130.61, 124.24, 124.20, 122.64, 122.16, 119.88, 119.53, 118.00, 117.34, 116.53, 57.89, 18.18.

HRMS $[C_{23}H_{18}ClN_3O_5 + H]^+$: 452.10077 calculated, 452.10045 found.

***N*-(4-(5-Methoxy-2-oxo-1,3,4-oxadiazol-3(2*H*)-yl)-2-methylphenyl)-3-(m-tolyloxy)benzamide (53)**

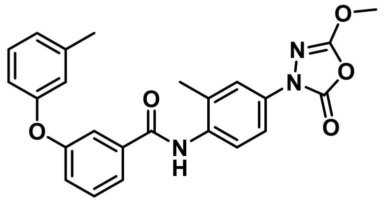

86g (62 mg, 0.271 mmol) was reacted with **12** (30 mg, 0.135 mmol) following General Procedure D. The crude product was purified by column chromatography (0% → 1% MeOH in DCM) to obtain **53** as a yellow oil (21 mg, 41 µmol, 31%).

^{1}H NMR (400 MHz, $CDCl_3$) δ 7.90 (d, *J* = 8.8 Hz, 1H), 7.78 (d, *J* = 6.5 Hz, 1H), 7.66 (d, *J* = 2.4 Hz, 1H), 7.63 – 7.53 (m, 2H), 7.50 (t, *J* = 2.0 Hz, 1H), 7.45 – 7.36 (m, 1H), 7.24 (t, *J* = 7.8 Hz, 1H), 7.16 (dt, *J* = 8.2, 1.6 Hz, 1H), 6.96 (ddt, *J* = 7.5, 1.7, 0.8 Hz, 1H), 6.87 – 6.79 (m, 2H), 4.10 (s, 3H), 2.34 (s, 3H), 2.32 (s, 3H).

^{13}C NMR (101 MHz, $CDCl_3$) δ 165.27, 158.22, 156.37, 155.86, 148.28, 140.31, 136.53, 133.21 (d, *J* = 10.9 Hz), 130.25, 129.75, 124.93, 123.87 (d, *J* = 33.0 Hz), 121.91, 121.32, 120.12, 119.75, 117.24, 116.41 (d, *J* = 4.8 Hz), 57.82, 21.47, 18.09.

HRMS $[C_{24}H_{21}N_3O_5 + H]^+$: 432.15540 calculated, 432.15524 found.

***N*-(4-(5-Methoxy-2-oxo-1,3,4-oxadiazol-3(2*H*)-yl)-2-methylphenyl)-3-(3-(trifluoromethyl)phenoxy)benzamide (54)**

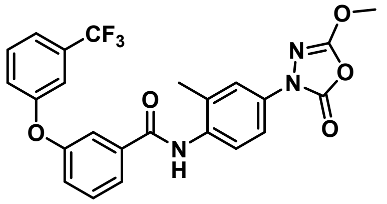

86h (77 mg, 0.27 mmol) was reacted with **12** (30 mg, 0.135 mmol) following General Procedure D. The crude product was purified by preparative HPLC to obtain **54** as a yellow oil (40 mg, 82 µmol, 61%).

^{1}H NMR (400 MHz, $CDCl_3$) δ 7.90 – 7.86 (m, 1H), 7.79 (s, 1H), 7.69 – 7.58 (m, 3H), 7.56 (t, *J* = 2.1 Hz, 1H), 7.51 – 7.44 (m, 2H), 7.41 – 7.37 (m, 1H), 7.27 (t, *J* = 2.0 Hz, 1H), 7.22 – 7.17 (m, 2H), 4.10 (s, 3H), 2.32 (s, 3H).

^{13}C NMR (101 MHz, $CDCl_3$) δ 164.98, 157.13, 157.10, 155.93, 148.33, 136.96, 133.44, 133.07, 132.56 (q, *J* = 32 Hz), 131.01, 130.71, 130.63, 124.19, 123.71 (q, *J* = 273 Hz), 122.55, 122.32, 122.22, 120.55 (q, *J* = 3.8 Hz) 119.82, 118.15, 116.44, 115.97 (q, *J* = 3.8 Hz), 57.87, 18.14.

HRMS $[C_{24}H_{18}F_3N_3O_5 + H]^+$: 486.12713 calculated, 486.12672 found.

Methyl (3-methyl-4-(3-phenoxybenzamido)phenyl)carbamate (55)

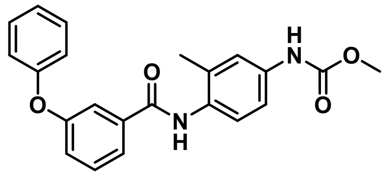

89 (20 mg, 63 µmol) was co-evaporated with toluene and dissolved in DCM (1 mL). Pyridine (10 µL, 0.126 mmol) was added, and the mixture was cooled down to 0°C. Methyl chloroformate (5.3 µL, 69 µmol) was added and the reaction was stirred for 4 h at RT. The mixture was resuspended in excessive amounts of EtOAc and 1

M aq. HCl. The organic layer was washed with brine and subsequently dried ($MgSO_4$), filtered and concentrated *in vacuo*. The resulting crude material was purified by column chromatography (70% Et_2O in pentane) to obtain title compound **55** as a white solid (13 mg, 35 µmol, 55%).

^1H NMR (400 MHz, $CDCl_3$) δ 7.68 (d, *J* = 7.7 Hz, 1H), 7.58 (t, *J* = 2.1 Hz, 1H), 7.46 (t, *J* = 8.0 Hz, 1H), 7.40 – 7.31 (m, 3H), 7.30 – 7.26 (m, 1H), 7.24 – 7.09 (m, 3H), 7.03 (d, *J* = 8.2 Hz, 2H), 3.73 (s, 3H), 2.24 (s, 3H).

^{13}C NMR (101 MHz, $CDCl_3$) δ 168.22, 158.81, 157.68, 156.23, 138.41, 137.16, 135.94, 131.43, 130.85, 130.76, 127.99, 124.67, 122.90, 122.64, 121.42, 119.99, 118.60, 117.50, 52.49, 18.38.

HRMS $[C_{22}H_{20}N_2O_4 + H]^+$: 377.14958 calculated, 377.14952 found.

***N*-(4-Acrylamido-2-methylphenyl)-3-phenoxybenzamide (56)**

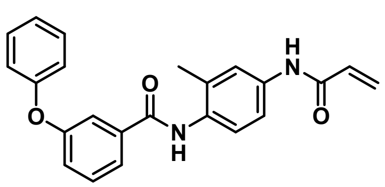

89 (20 mg, 63 µmol) was co-evaporated with toluene and dissolved in DCM (1 mL). Pyridine (10 µL, 0.126 mmol) was added, and the mixture was cooled down to 0°C. Acryloyl chloride (5.3 µL, 69 µmol) was added and the reaction was stirred for 4 h at RT. The mixture was resuspended in excessive amounts of EtOAc and 1 M aq. HCl. The organic layer was washed with brine and subsequently dried ($MgSO_4$), filtered and concentrated *in vacuo*. The resulting crude material was purified by column chromatography (80% Et_2O in pentane) to obtain title compound **56** as a clear oil (10 mg, 27 µmol, 43%).

^1H NMR (300 MHz, MeOD) δ 7.67 – 7.60 (m, 1H), 7.55 (dd, *J* = 8.1, 2.3 Hz, 2H), 7.45 – 7.27 (m, 5H), 7.18 – 7.06 (m, 2H), 7.04 – 6.97 (m, 2H), 6.35 (d, *J* = 5.9 Hz, 2H), 5.71 (t, *J* = 5.9 Hz, 1H), 2.24 (s, 3H).

^{13}C NMR (75 MHz, MeOD) δ 167.25, 165.23, 158.21, 157.01, 137.03, 136.64, 134.48, 132.03, 131.54, 130.42, 130.31, 127.57, 126.75, 125.78, 124.25, 122.62, 122.34, 122.23, 119.58, 118.68, 118.12, 18.21.

HRMS $[C_{23}H_{20}N_2O_3 + H]^+$: 373.1547 calculated, 373.1545 found.

***N*-(4-(2-Chloroacetamido)-2-methylphenyl)-3-phenoxybenzamide (57)**

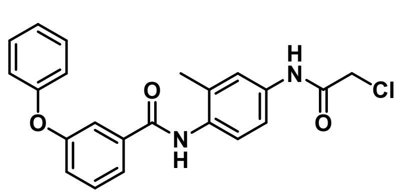

89 (30 mg, 94 µmol) was co-evaporated with toluene and dissolved in DCM (1 mL). K_2CO_3 (13 mg, 94 µmol) was added and the mixture was cooled down to 0°C. Chloroacetyl chloride (7.5 µL, 94 µmol) was added dropwise and the reaction was stirred overnight at RT. H_2O (2 mL) was added and mixture was washed with 1 M aq. HCl. The organic layer was separated and dried ($MgSO_4$), filtered and concentrated *in vacuo*. The resulting crude material was purified by column chromatography (20% → 50% EtOAc in pentane) to obtain title compound **57** as a white solid (9.0 mg, 24 µmol, 25%).

^{1}H NMR (400 MHz, DMSO) δ 10.29 (s, 1H), 9.90 (s, 1H), 7.76 (d, *J* = 7.7 Hz, 1H), 7.60 - 7.57 (m, 1H), 7.54 (t, *J* = 8.0 Hz, 1H), 7.49 (d, *J* = 2.4 Hz, 1H), 7.46 – 7.40 (m, 3H), 7.28 – 7.15 (m, 3H), 7.10 – 7.04 (m, 2H), 4.25 (s, 2H), 2.19 (s, 3H).

^{13}C NMR (101 MHz, DMSO) δ 170.40, 164.57, 156.92, 156.28, 136.40, 136.38, 134.46, 132.03, 130.24, 127.25, 123.93, 122.52, 121.59, 121.06, 119.01, 117.50, 117.19, 43.61, 18.15.

HRMS $[C_{22}H_{19}ClN_2O_3 + H]^+$: 395.11570 calculated, 395.11573 found.

***N*-(4-Isothiocyanato-2-methylphenyl)-3-phenoxybenzamide (58)**

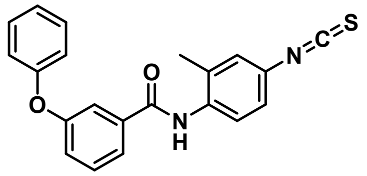

89 (20 mg, 63 µmol) was co-evaporated with toluene and dissolved in H_2O and DCM (0.5 mL, 1:1). $NaHCO_3$ (18 mg, 0.22 mmol) was added and the mixture was cooled down to 0°C. Thiophosgene (6.0 µL, 94 µmol) was dissolved in H_2O and DCM (0.5 mL, 1:1) and this solution was added dropwise to the stirred reaction mixture, which was then stirred for 30 min. The layers were separated and the aqueous layer was extracted with DCM (3×). The combined organic layers were dried ($MgSO_4$), filtered and concentrated *in vacuo*, which yieded the title compound **58** as a white solid (10 mg, 28 µmol, 44%).

^{1}H NMR (400 MHz, $CDCl_3$) δ 8.01 (d, *J* = 8.4 Hz, 1H), 7.64 (s, 1H), 7.55 (ddd, *J* = 7.7, 1.7, 1.0 Hz, 1H), 7.51 – 7.42 (m, 2H), 7.42 – 7.32 (m, 2H), 7.23 – 7.00 (m, 6H), 2.29 (s, 3H).

^{13}C NMR (101 MHz, $CDCl_3$) δ 165.02, 158.33, 156. 37, 136.50, 135.15, 135.03, 130.49, 130.22, 130.18, 127.76, 127.65, 124.40, 124.29, 123.56, 122.15, 121.32, 119.58, 117.17, 17.79.

HRMS $[C_{21}H_{17}N_3O_2 + H]^+$: 361.15467 calculated, 361.15428 found.

***N*-(4-(5-Ethoxy-2-oxo-1,3,4-oxadiazol-3(2*H*)-yl)-2-methylphenyl)-3-phenoxybenzamide (59)**

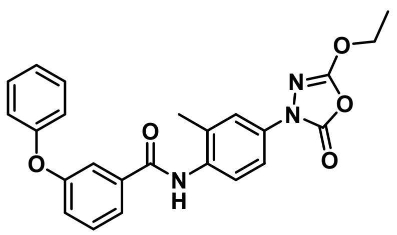

3-Phenoxybenzoic acid (73 mg, 0.34 mmol) was reacted with **67f** (40 mg, 0.17 mmol) following General Procedure D. The crude product was purified by column chromatography (1% MeOH in DCM) to obtain the title compound as a clear oil (34 mg, 67 µmol, 40%).

^{1}H NMR (500 MHz, $CDCl_3$) δ 8.00 – 7.90 (m, 1H), 7.71 – 7.68 (m, 1H), 7.66 (bs, 1H), 7.63 (dd, *J* = 8.8, 2.6 Hz, 1H), 7.57 (d, *J* = 7.6, 1H), 7.52 – 7.49 (m, 1H), 7.44 (t, *J* = 7.9 Hz, 1H), 7.41 – 7.35 (m, 2H), 7.21 – 7.13 (m, 2H), 7.07 – 7.03 (m, 2H), 4.48 (q, *J* = 7.1 Hz, 2H), 2.34 (s, 3H), 1.50 (t, *J* = 7.1 Hz, 3H).

^{13}C NMR (126 MHz, $CDCl_3$) δ 164.83, 158.26, 156.51, 155.28, 148.39, 136.77, 133.41, 133.21, 130.54, 130.40, 130.15, 124.20, 123.91, 122.02, 121.42, 119.89, 119.54, 117.31, 116.59, 67.91, 18.15, 14.25.

HRMS $[C_{24}H_{21}N_3O_5 + H]^+$: 432.15540 calculated, 432.15526 found.

N-(4-(5-Isobutoxy-2-oxo-1,3,4-oxadiazol-3(2H)-yl)-2-methylphenyl)-3-phenoxybenzamide (60)

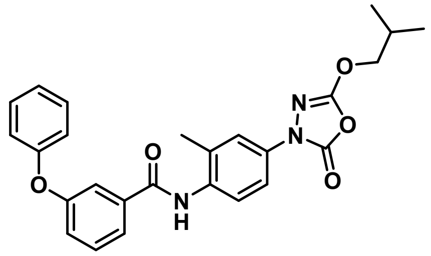

3-Phenoxybenzoic acid (68 mg, 0.32 mmol) was reacted with **67g** (42 mg, 0.16 mmol) following General Procedure D. The crude product was purified by column chromatography (1% MeOH in DCM) to obtain the title compound as a clear oil (77 mg, 0.15 mmol, 95%).

^{1}H NMR (500 MHz, $CDCl_3$) δ 7.91 (d, *J* = 8.8 Hz, 1H), 7.72 (s, 1H), 7.69 – 7.64 (m, 1H), 7.61 (dd, *J* = 8.8, 2.6 Hz, 1H), 7.57 (d, *J* = 7.9, 1H), 7.52 – 7.49 (m, 1H), 7.43 (t, *J* = 7.9 Hz, 1H), 7.40 – 7.32 (m, 2H), 7.20 – 7.11 (m, 2H), 7.07 – 6.97 (m, 2H), 4.17 (d, *J* = 6.6 Hz, 2H), 2.32 (s, 3H), 2.16 (non, *J* = 6.8 Hz, 1H), 1.04 (d, *J* = 6.8 Hz, 6H).

^{13}C NMR (126 MHz, $CDCl_3$) δ 165.18, 158.20, 156.51, 155.51, 148.37, 136.73, 133.42, 133.17, 130.74, 130.35, 130.12, 124.15, 124.02, 121.99, 121.45, 119.85, 119.51, 117.34, 116.52, 77.55, 27.88, 18.81, 18.13.

HRMS $[C_{26}H_{25}N_3O_5 + H]^+$: 460.18670 calculated, 460.18640 found.

N-(4-(5-Butoxy-2-oxo-1,3,4-oxadiazol-3(2H)-yl)-2-methylphenyl)-3-phenoxybenzamide (61)

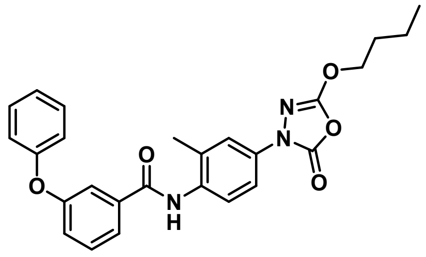

3-Phenoxybenzoic acid (81 mg, 0.38 mmol) was reacted with **67h** (50 mg, 0.19 mmol) following General Procedure D. The crude product was purified by column chromatography (1% MeOH in DCM) to obtain the title compound as a clear oil (82 mg, 0.16 mmol, 85%).

^{1}H NMR (500 MHz, $CDCl_3$) δ 7.94 (d, *J* = 8.8 Hz, 1H), 7.72 – 7.64 (m, 2H), 7.62 (dd, *J* = 8.8, 2.6 Hz, 1H), 7.57 (d, *J* = 7.8 Hz, 1H), 7.52 – 7.49 (m, 1H), 7.44 (t, *J* = 7.9 Hz, 1H), 7.40 – 7.33 (m, 2H), 7.20 – 7.12 (m, 2H), 7.07 – 6.99 (m, 2H), 4.40 (t, *J* = 6.5 Hz, 2H), 2.33 (s, 3H), 1.85 – 1.78 (m, 2H), 1.49 (sext, *J* = 7.4 Hz, 2H), 1.00 (t, *J* = 7.4 Hz, 3H).

^{13}C NMR (126 MHz, $CDCl_3$) δ 165.16, 158.23, 156.51, 155.44, 148.39, 136.76, 133.42, 133.19, 130.61, 130.37, 130.13, 124.18, 123.95, 122.00, 121.43, 119.87, 119.53, 117.32, 116.56, 71.66, 30.52, 18.88, 18.14, 13.69.

HRMS $[C_{26}H_{25}N_3O_5 + H]^+$: 460.18670 calculated, 460.18651 found.

N-(4-(5-(2-Methoxyethoxy)-2-oxo-1,3,4-oxadiazol-3(2H)-yl)-2-methylphenyl)-3-phenoxybenzamide (62)

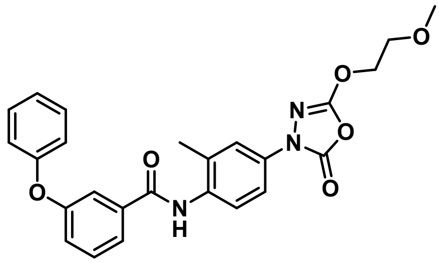

3-Phenoxybenzoic acid (84 mg, 0.39 mmol) was reacted with **67i** (52 mg, 0.20 mmol) following General Procedure D. The crude product was purified by column

chromatography (1% MeOH in DCM) to obtain the title compound as a clear oil (71 mg, 0.14 mmol, 70%).

^{1}H NMR (500 MHz, $CDCl_3$) δ 7.95 (d, *J* = 8.8 Hz, 1H), 7.71 – 7.64 (m, 2H), 7.61 (dd, *J* = 8.8, 2.6 Hz, 1H), 7.57 (d, *J* = 7.8 Hz, 1H), 7.52 – 7.49 (m, 1H), 7.44 (t, *J* = 7.9 Hz, 1H), 7.41 – 7.31 (m, 2H), 7.21 – 7.10 (m, 2H), 7.09 – 6.99 (m, 2H), 4.59 – 4.48 (m, 2H), 3.81 – 3.72 (m, 2H), 3.44 (s, 3H), 2.33 (s, 3H).

^{13}C NMR (126 MHz, $CDCl_3$) δ 165.16, 158.24, 156.51, 155.34, 148.29, 136.74, 133.32, 133.27, 130.61, 130.39, 130.14, 124.19, 123.93, 122.01, 121.43, 119.90, 119.53, 117.31, 116.58, 70.57, 69.71, 59.33, 18.14.

HRMS [$C_{25}H_{23}N_3O_6$ + H]$^+$: 462.16596 calculated, 462.16577 found.

(3-Methyl-4-nitrophenyl)hydrazine (64a)

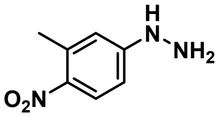

4-Fluoro-2-methyl-1-nitrobenzene (10.0 g, 64.5 mmol) was suspended in ethanol (100 mL), after which hydrazine monohydrate (10.0 mL, 191 mmol) was added dropwise. The mixture was heated to 80°C, and stirred for 16 h. The mixture was then transferred to a beaker and cooled to 0°C, which formed an orange precipitate. The mixture was filtered, and washed on the filter with ice cold EtOH (100 mL). The residue was collected and concentrated *in vacuo* to give the title compound as an orange powder (8.60 g, 51.4 mmol, 80%).

^{1}H NMR (400 MHz, DMSO) δ 8.17 (s, 1H), 7.95 (d, *J* = 9.2 Hz, 1H), 6.67 – 6.61 (m, 2H), 4.41 (s, 2H), 2.50 (s, 3H).

^{13}C NMR (101 MHz, DMSO) δ 156.21, 137.16, 136.03, 128.02, 112.12, 108.19, 22.46.

(3-Methoxy-4-nitrophenyl)hydrazine (64c)

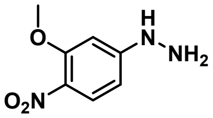

4-Fluoro-2-methoxy-1-nitrobenzene (1.00 g, 5.84 mmol) was dissolved in NMP (1 mL), after which hydrazine hydrate (700 µL, 13.4 mmol) was added dropwise. The mixture was heated to 65°C and stirred for 75 min, after which the mixture was cooled to RT and H_2O (10 mL) was added. The precipitate was put over a filter, and subsequently the precipitate was recrystallized from isopropanol to give the title compound as a yellow solid (790 mg, 4.31 mmol, 74%).

^{1}H NMR (400 MHz, DMSO) δ 8.26 (s, 1H), 7.84 (d, *J* = 9.3 Hz, 1H), 6.50 (s, 1H), 6.32 (d, *J* = 9.3 Hz, 1H), 4.47 (s, 2H), 3.84 (s, 3H).

^{13}C NMR (101 MHz, DMSO) δ 158.15, 157.00, 128.72, 126.40, 102.96, 92.48, 55.91.

(3-Chloro-4-nitrophenyl)hydrazine (64d)

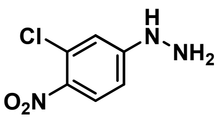

2-Chloro-4-fluoro-1-nitrobenzene (1.00 g, 5.70 mmol) was dissolved in NMP (1 mL), after which hydrazine hydrate (700 µL, 13.4 mmol) was added dropwise. The mixture was heated to 65°C and stirred for 45 min, after which the mixture was cooled to RT and H_2O (10 mL) was added. The precipitate was put over a filter, and subsequently the precipitate was recrystallized from isopropanol to give the title compound as orange crystals (817 mg, 4.36 mmol, 76%).

^{1}H NMR (400 MHz, DMSO) δ 8.47 (s, 1H), 7.98 (d, *J* = 9.3 Hz, 1H), 6.88 (s, 1H), 6.70 (d, *J* = 9.6 Hz, 1H), 4.55 (s, 2H).

^{13}C NMR (101 MHz, DMSO) δ 156.85, 133.85, 129.98, 129.62, 111.58, 109.28.

5-Hydrazineyl-2-nitrobenzonitrile (64e)

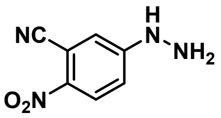

5-Fluoro-2-benzonitrile (500 mg, 3.01 mmol) was dissolved in NMP (5 mL), after which the mixture was cooled to 0°C, followed by dropwise addition of hydrazine hydrate (250 µL, 4.78 mmol). After stirring for 4 h, H_2O (45 mL) was added to the mixture, and the precipitate was put over a filter. The residue was recrystallized from MeOH to give the title compound as a yellow solid (415 mg, 2.33 mmol, 77%).

^{1}H NMR (400 MHz, DMSO) δ 8.85 (s, 1H), 8.13 (d, *J* = 9.4 Hz, 1H), 7.33 – 6.82 (m, 2H), 4.70 (s, 2H).

^{13}C NMR (101 MHz, DMSO) δ 156.05, 134.26, 128.33, 116.66, 115.58, 112.56, 109.20.

Methyl 2-(3-methyl-4-nitrophenyl)hydrazine-1-carboxylate (65a)

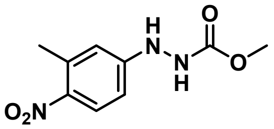

64a (203 mg, 1.21 mmol) and NMP (104 µL, 1.09 mmol) were dissolved in pyridine (1.2 mL). The mixture was cooled to 0°C, after which methyl chloroformate (0.141 mL, 1.80 mmol) was added dropwise. The reaction mixture was stirred for 90 min, after which the mixture was resuspended in excessive amounts of EtOAc and 1 M aq. HCl. The organic layer was washed with brine, and subsequently dried ($MgSO_4$), filtered and concentrated *in vacuo*. Purification of the crude material by column chromatography (10% → 50% EtOAc in pentane) yielded the title compound as a yellow oil (246 mg, 1.09 mmol, 90%).

^{1}H NMR (400 MHz, DMSO) δ 9.38 (bs, 1H), 8.80 (s, 1H), 7.99 (d, *J* = 9.1 Hz, 1H), 6.60 (dd, *J* = 9.1, 2.6 Hz, 1H), 6.58 – 6.50 (m, 1H), 3.63 (s, 3H), 2.52 (s, 3H).

^{13}C NMR (101 MHz, DMSO) δ 157.02, 153.78, 138.71, 136.78, 127.81, 113.15, 108.87, 52.17, 21.77.

Methyl 2-(4-nitrophenyl)hydrazine-1-carboxylate (65b)

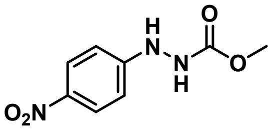

1-Fluoro-4-nitrobenzene (**63b**) (706 mg, 5.0 mmol) was suspended in ethanol (10 mL), after which hydrazine monohydrate (0.78 mL, 15 mmol) was added dropwise. The mixture was heated to 80°C, and stirred for 3 h. The mixture was then transferred cooled to 0°C, which formed an orange precipitate. The mixture was filtered, and washed on the filter with ice cold EtOH (50 mL). The remaining residue was collected and concentrated *in vacuo* to give the hydrazine intermediate as orange crystals (146 mg, 0.95 mmol, 19%). The crystals and NMP (83 µL, 0.86 mmol) were dissolved in pyridine (3 mL). The mixture was cooled to 0°C, after which methyl chloroformate (81 µL, 1.05 mmol) was added dropwise. The reaction mixture was stirred for 16 h, after which the mixture was resuspended in EtOAc and 1 M aq. HCl, followed by extraction with EtOAc (2×). The organic layers were washed with brine, and subsequently dried ($MgSO_4$), filtered and concentrated *in vacuo* to yield the title compound (122 mg, 0.58 mmol, 61%).

^1H NMR (400 MHz, DMSO) δ 9.46 (s, 1H), 9.01 (s, 1H), 8.11 – 8.06 (m, 2H), 6.78 – 6.72 (m, 2H), 3.65 (s, 3H).

^{13}C NMR (101 MHz, DMSO) δ 162.93, 154.49, 138.08, 126.01, 110.33, 52.18.

Methyl 2-(3-methoxy-4-nitrophenyl)hydrazine-1-carboxylate (65c)

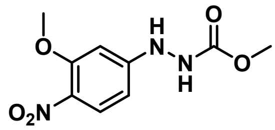

64c (172 mg, 0.94 mmol) and NMP (81 µL, 0.85 mmol) were dissolved in pyridine (3 mL). The mixture was cooled to 0°C, after which methyl chloroformate (80 µL, 1.03 mmol) was added dropwise. The reaction mixture was stirred for 90 min, after which the mixture was resuspended in excessive amounts of EtOAc and 1 M aq. HCl. The organic layer was washed with brine, and subsequently dried ($MgSO_4$), filtered and concentrated *in vacuo* to yield the title compound (103 mg, 0.43 mmol, 46%).

^1H NMR (400 MHz, $CDCl_3$) δ 7.89 (d, *J* = 9.0 Hz, 1H), 7.80 (s, 1H), 7.27 (s, 1H), 6.40 (d, *J* = 2.2 Hz, 1H), 6.35 (dd, *J* = 9.0, 2.2 Hz, 1H), 3.85 (s, 3H), 3.75 (s, 3H).

^{13}C NMR (101 MHz, $CDCl_3$) δ 157.61, 156.26, 154.75, 130.78, 128.78, 103.36, 95.38, 56.22, 53.04.

Methyl 2-(3-chloro-4-nitrophenyl)hydrazine-1-carboxylate (65d)

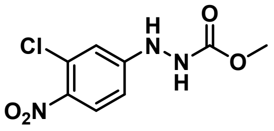

64d (180 mg, 0.960 mmol) and pyridine (155 µL, 1.92 mmol) were dissolved in DCM (5 mL). The mixture was cooled to 0°C, after which methyl chloroformate (82.0 µL, 1.06 mmol) was added dropwise. After stirring for 16 h 1 M aq. HCl was added, the layers were separated, and the aqueous layer was extracted twice with EtOAc. The combined organic layers were washed with brine, dried ($MgSO_4$), filtered, and concentrated *in vacuo* to yield the title compound as a yellow oil (220 mg, 0.90 mmol, 93%).

^{1}H NMR (400 MHz, DMSO) δ 9.49 (s, 1H), 9.07 (s, 1H), 8.04 (d, *J* = 9.1 Hz, 1H), 6.77 (d, *J* = 2.4 Hz, 1H), 6.72 (dd, *J* = 9.1, 2.6 Hz, 1H), 3.64 (s, 3H).

^{13}C NMR (101 MHz, DMSO) δ 156.90, 156.30, 154.11, 136.50, 129.11, 112.11, 109.65, 52.35.

Methyl 2-(3-cyano-4-nitrophenyl)hydrazine-1-carboxylate (65e)

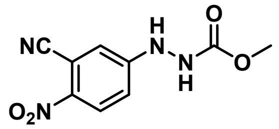

64e (190 mg, 1.07 mmol) was dissolved in pyridine (5 mL). The mixture was cooled to 0°C, after which methyl chloroformate (91 µL, 1.17 mmol) was added dropwise. After stirring for 20 min 1 M aq. HCl was added, the layers were separated, and the aqueous layer was extracted with EtOAc (2×). The combined organic layers were washed with brine, dried ($MgSO_4$), filtered, and concentrated *in vacuo* to yield the title compound as an off-white solid (232 mg, 0.98 mmol, 92%).

^{1}H NMR (400 MHz, DMSO) δ 9.39 (d, *J* = 3.3 Hz, 1H), 8.24 (d, *J* = 9.3, 1H), 7.14 (s, 1H), 7.01 (d, *J* = 9.3, 1H), 3.65 (s, 3H).

^{13}C NMR (101 MHz, DMSO) δ 156.71, 154.16, 137.57, 128.42, 113.77, 109.08, 52.42.

Ethyl 2-(3-methyl-4-nitrophenyl)hydrazine-1-carboxylate (65f)

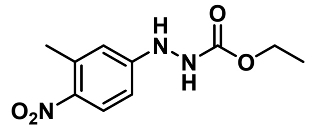

64a (502 mg, 3.00 mmol) and pyridine (485 µL, 6.00 mmol) were dissolved in DCM (20 mL). The mixture was cooled to 0°C, after which ethyl chloroformate (325 µL, 3.30 mmol) was added dropwise. The reaction mixture was stirred for 15 min and was then warmed up to RT to stir for 30 additional min. The mixture was resuspended in excessive amounts of EtOAc and 1 M aq. HCl. The organic layer was washed with brine, and subsequently dried ($MgSO_4$), filtered and concentrated *in vacuo* without any further purification to yield the title compound as a yellow oil (500 mg, 2.09 mmol, 70%).

^{1}H NMR (500 MHz, $CDCl_3$) δ 7.99 (d, *J* = 9.0 Hz, 1H), 6.84 (s, 1H), 6.63 (dd, *J* = 9.0, 2.6 Hz, 1H), 6.59 (d, *J* = 2.6 Hz, 1H), 4.22 (q, *J* = 7.1 Hz, 2H), 2.54 (s, 3H), 1.40 – 1.20 (m, 3H).

^{13}C NMR (126 MHz, $CDCl_3$) δ 157.10, 152.29, 141.35, 137.26, 127.71, 114.91, 109.75, 62.53, 22.01, 14.54.

Isobutyl 2-(3-methyl-4-nitrophenyl)hydrazine-1-carboxylate (65g)

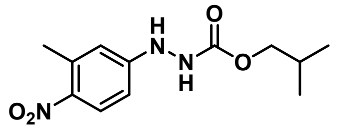

64a (502 mg, 3.00 mmol) and pyridine (485 µL, 6.00 mmol) were dissolved in DCM (20 mL). The mixture was cooled to 0°C, after which isobutyl chloroformate (437 µL, 3.30 mmol) was added dropwise. The reaction mixture was stirred for 15 min and was then warmed up to RT to stir for 30 additional min. The mixture was resuspended in excessive amounts of EtOAc and 1 M aq. HCl. The organic layer was washed with brine, and subsequently dried ($MgSO_4$), filtered and concentrated *in vacuo* without any further purification to yield the title compound as a yellow oil (732 mg, 2.74 mmol, 91%).

^1H NMR (500 MHz, $CDCl_3$) δ 7.99 (d, *J* = 9.0 Hz, 1H), 6.86 (s, 1H), 6.64 (dd, *J* = 9.0, 2.6 Hz, 1H), 6.59 (d, *J* = 2.7 Hz, 1H), 3.95 (d, *J* = 6.7 Hz, 2H), 2.54 (s, 3H), 1.97 (m, 1H), 1.10 – 0.33 (m, 6H).

^{13}C NMR (126 MHz, $CDCl_3$) δ 157.24, 152.33, 141.34, 137.26, 127.72, 114.92, 109.74, 72.43, 28.01, 22.02, 18.93.

Butyl 2-(3-methyl-4-nitrophenyl)hydrazine-1-carboxylate (65h)

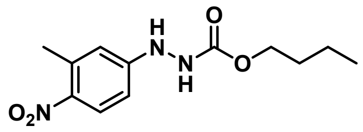

64a (502 mg, 3.00 mmol) and pyridine (485 µL, 6.00 mmol) were dissolved in DCM (20 mL). The mixture was cooled to 0°C, after which butyl chloroformate (428 µL, 3.30 mmol) was added dropwise. The reaction mixture was stirred for 15 min and was then warmed up to RT to stir for 30 additional min. The mixture was resuspended in excessive amounts of EtOAc and 1 M aq. HCl. The organic layer was washed with brine, and subsequently dried ($MgSO_4$), filtered and concentrated *in vacuo* without any further purification to yield the title compound as a yellow oil (684 mg, 2.56 mmol, 85%).

^1H NMR (500 MHz, $CDCl_3$) δ 7.96 (d, *J* = 9.0 Hz, 1H), 6.96 (bs, 1H), 6.83 (bs, 1H), 6.61 (dd, *J* = 9.0, 2.6 Hz, 1H), 6.56 (d, *J* = 2.6 Hz, 1H), 4.16 (t, *J* = 6.7 Hz, 2H), 2.51 (s, 3H), 1.83 – 1.51 (m, 2H), 1.51 – 1.11 (m, 2H), 1.11 – 0.49 (m, 3H).

^{13}C NMR (126 MHz, $CDCl_3$) δ 157.30, 152.38, 141.07, 137.21, 127.65, 114.78, 109.62, 66.28, 30.85, 21.96, 18.94, 13.67.

2-Methoxyethyl 2-(3-methyl-4-nitrophenyl)hydrazine-1-carboxylate (65i)

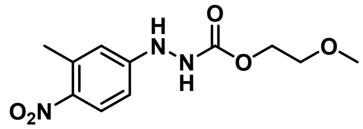

64a (502 mg, 3.00 mmol) and pyridine (485 µL, 6.00 mmol) were dissolved in DCM (20 mL). The mixture was cooled to 0°C, after which 2-methoxyethyl chloroformate (384 µL, 3.30 mmol) was added dropwise. The reaction mixture was stirred for 15 min and was then warmed up to RT to stir for 30 additional min. The mixture was resuspended in excessive amounts of EtOAc and 1 M aq. HCl. The organic layer was washed with brine, and subsequently dried ($MgSO_4$), filtered and concentrated *in vacuo* without any further purification to yield the title compound as a yellow oil (750 mg, 2.79 mmol, 93%).

^1H NMR (400 MHz, $CDCl_3$) δ 8.02 (d, *J* = 8.9 Hz, 1H), 6.97 (s, 1H), 6.66 (dd, *J* = 8.9, 2.6 Hz, 1H), 6.64 – 6.60 (m, 1H), 6.49 (s, 1H), 4.33 (t, *J* = 4.5 Hz, 2H), 3.68 – 3.57 (m, 2H), 3.48 – 3.32 (m, 3H), 2.57 (s, 3H).

^{13}C NMR (101 MHz, $CDCl_3$) δ 156.84, 152.11, 141.57, 137.29, 127.75, 115.00, 109.89, 70.58, 65.24, 59.06, 22.05.

5-Methoxy-3-(3-methyl-4-nitrophenyl)-1,3,4-oxadiazol-2(3*H*)-one (66a)

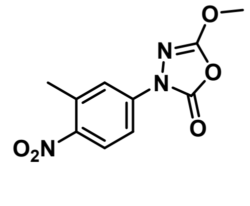

65a (500 mg, 2.22 mmol) was reacted according to General Procedure E. Purification of the crude material by column chromatography (5% → 10%

EtOAc in pentane) yielded the desired product as an off-white solid (484 mg, 1.93 mmol, 87%).

^{1}H NMR (400 MHz, DMSO) δ 8.16 (d, *J* = 8.9 Hz, 1H), 7.74 (dd, *J* = 8.9, 2.5 Hz, 1H), 7.71 (d, *J* = 2.5 Hz, 1H), 4.11 (s, 3H), 2.58 (s, 3H).

^{13}C NMR (101 MHz, DMSO) δ 155.78, 147.85, 144.79, 139.52, 135.37, 126.64, 119.86, 115.16, 58.43, 20.47.

5-Methoxy-3-(4-nitrophenyl)-1,3,4-oxadiazol-2(3*H*)-one (66b)

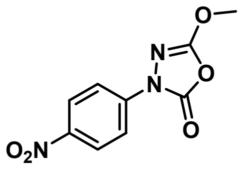

65b (122 mg, 0.58 mmol) was reacted according to General Procedure E. Purification of the crude material by column chromatography (10% → 50% EtOAc in pentane) yielded the desired product as an off-white solid (50 mg, 0.21 mmol, 37%).

^{1}H NMR (400 MHz, $CDCl_3$) δ 8.32 (d, *J* = 8.8 Hz, 2H), 8.03 (d, *J* = 8.9 Hz, 2H), 4.18 (s, 3H).

^{13}C NMR (75 MHz, $CDCl_3$) δ 144.47, 141.16, 125.16, 117.64, 58.22.

5-Methoxy-3-(3-methoxy-4-nitrophenyl)-1,3,4-oxadiazol-2(3H)-one (66c)

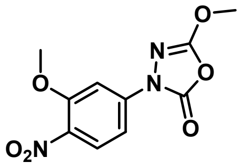

65c (106 mg, 0.44 mmol) was reacted according to General Procedure E. Purification of the crude material by column chromatography (DCM) yielded the desired product as an off-white solid (23 mg, 86 µmol, 20%).

^{1}H NMR (400 MHz, $CDCl_3$) δ 8.01 (d, *J* = 9.0 Hz, 1H), 7.67 (d, *J* = 2.2 Hz, 1H), 7.46 (dd, *J* = 9.0, 2.2 Hz, 1H), 4.16 (s, 3H), 4.02 (s, 3H).

^{13}C NMR (101 MHz, $CDCl_3$) δ 156.27, 154.60, 147.98, 141.11, 135.94, 127.62, 108.85, 102.25, 58.25, 56.88.

3-(3-Chloro-4-nitrophenyl)-5-methoxy-1,3,4-oxadiazol-2(3*H*)-one (66d)

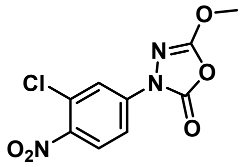

65d (103 mg, 0.42 mmol) was reacted according to General Procedure E. Purification of the crude material by column chromatography (DCM) yielded the desired product as an off-white solid (48 mg, 0.18 mmol, 42%).

^{1}H NMR (400 MHz, $CDCl_3$) δ 8.07 – 8.01 (m, 2H), 7.91 (dd, *J* = 9.1, 2.3 Hz, 1H), 4.17 (s, 3H).

^{13}C NMR (101 MHz, $CDCl_3$) δ 156.46, 147.74, 143.94, 139.76, 129.10, 127.18, 120.05, 115.79, 58.38.

5-(5-Methoxy-2-oxo-1,3,4-oxadiazol-3(2*H*)-yl)-2-nitrobenzonitrile (66e)

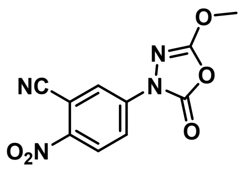

65e (140 mg, 0.59 mmol) was reacted according to General Procedure E. Purification of the crude material by column chromatography (DCM) yielded the desired product as a yellow solid (26 mg, 99 µmol, 17%).

^1H NMR (400 MHz, $CDCl_3$) δ 8.43 (d, *J* = 9.2 Hz, 1H), 8.36 (d, *J* = 2.3 Hz, 1H), 8.32 (t, *J* = 2.3 Hz, 1H), 4.21 (s, 3H).

^{13}C NMR (101 MHz, $CDCl_3$) δ 156.75, 153.29, 147.61, 140.78, 129.93, 127.33, 123.38, 114.64, 109.67, 58.63.

5-Ethoxy-3-(3-methyl-4-nitrophenyl)-1,3,4-oxadiazol-2(3*H*)-one (66f)

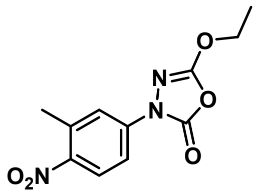

65f (500 mg, 2.09 mmol) was reacted according to General Procedure E. Purification of the crude material by column chromatography (6% EtOAc in pentane) yielded the desired product as a yellow solid (420 mg, 1.58 mmol, 76%).

^1H NMR (300 MHz, $CDCl_3$) δ 8.07 – 7.93 (m, 1H), 7.77 – 7.62 (m, 2H), 4.49 (q, *J* = 7.1 Hz, 2H), 2.60 (s, 3H), 1.51 (t, *J* = 7.1 Hz, 3H).

^{13}C NMR (75 MHz, $CDCl_3$) δ 155.46, 147.80, 145.19, 139.55, 135.89, 126.32, 120.42, 115.19, 68.35, 21.16, 14.04.

5-Isobutoxy-3-(3-methyl-4-nitrophenyl)-1,3,4-oxadiazol-2(3*H*)-one (66g)

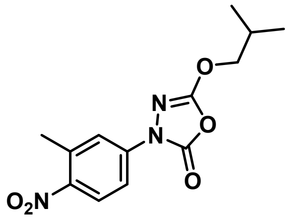

65g (732 mg, 2.74 mmol) was reacted according to General Procedure E. Purification of the crude material by column chromatography (5% EtOAc in pentane) yielded the desired product as a yellow solid (673 mg, 2.29 mmol, 84%).

^1H NMR (300 MHz, $CDCl_3$) δ 7.98 – 7.92 (m, 1H), 7.70 – 7.62 (m, 2H), 4.17 (d, *J* = 6.6 Hz, 2H), 2.56 (s, 3H), 2.14 (nonet, *J* = 6.7 Hz, 1H), 1.02 (d, *J* = 6.7 Hz, 6H).

^{13}C NMR (75 MHz, $CDCl_3$) δ 155.63, 147.68, 145.03, 139.46, 135.75, 126.18, 120.27, 115.05, 77.77, 27.64, 21.04, 18.52.

5-Butoxy-3-(3-methyl-4-nitrophenyl)-1,3,4-oxadiazol-2(3*H*)-one (66h)

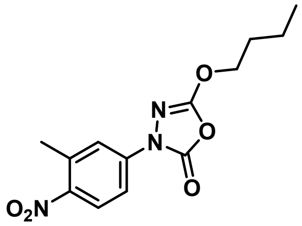

65h (684 mg, 2.56 mmol) was reacted according to General Procedure E. Purification of the crude material by column chromatography (5% EtOAc in pentane) yielded the desired product as a yellow oil (565 mg, 1.93 mmol, 75%).

^{13}C NMR (75 MHz, $CDCl_3$) δ 155.62, 147.81, 145.18, 139.56, 135.88, 126.32, 120.42, 115.20, 72.02, 30.28, 21.15, 18.70, 13.52.

5-(2-Methoxyethoxy)-3-(3-methyl-4-nitrophenyl)-1,3,4-oxadiazol-2(3*H*)-one (66i)

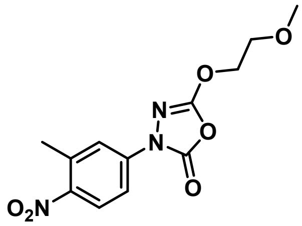

65i (750 mg, 2.79 mmol) was reacted according to General Procedure E. Purification of the crude material by column

chromatography (10% EtOAc in pentane) yielded the desired product as an off-white solid (538 mg, 1.82 mmol, 65%).

^{1}H NMR (400 MHz, $CDCl_3$) δ 8.11 (dd, *J* = 8.5, 0.9 Hz, 1H), 7.83 – 7.76 (m, 2H), 4.61 – 4.55 (m, 2H), 3.82 – 3.77 (m, 2H), 3.45 (s, 3H), 2.68 (s, 3H).

^{13}C NMR (101 MHz, $CDCl_3$) δ 155.72, 147.91, 145.56, 139.63, 136.13, 126.59, 120.75, 115.51, 70.96, 69.58, 59.35, 21.37.

3-(4-Aminophenyl)-5-methoxy-1,3,4-oxadiazol-2(3*H*)-one (67b)

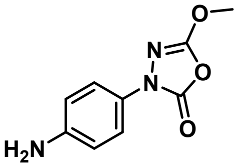

66b (50 mg, 0.21 mmol) was dissolved in DCM (3 mL) and MeOH (3 mL), and was reacted with Pd/C catalyst (10 wt%, 150 mg, 0.14 mmol) following General Procedure F. The filtrate was concentrated to give the title compound as an orange solid (35 mg, 0.17 mmol, 80%).

^{1}H NMR (400 MHz, $CDCl_3$) δ 7.51 (d, *J* = 8.6 Hz, 2H), 6.70 (d, *J* = 8.6 Hz, 2H), 4.07 (s, 3H), 3.80 (bs, 2H).

^{13}C NMR (101 MHz, $CDCl_3$) δ 155.76, 148.66, 144.72, 127.60, 120.39, 115.33, 57.66, 29.79.

3-(4-Amino-3-methoxyphenyl)-5-methoxy-1,3,4-oxadiazol-2(3*H*)-one (67c)

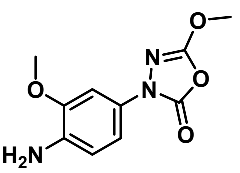

66c (23 mg, 86 µmol) was dissolved in DCM (3 mL) and MeOH (3 mL) was reacted with Pd/C catalyst (10 wt%, 10 mg, 9.4 µmol) following General Procedure F. The title compound was obtained as an off-white solid (19 mg, 80 µmol, 93%) without further need for purification.

^{1}H NMR (400 MHz, $CDCl_3$) δ 7.24 (d, *J* = 2.3 Hz, 1H), 7.14 (dd, *J* = 8.4, 2.3 Hz, 1H), 6.72 (d, *J* = 8.4 Hz, 1H), 4.08 (s, 3H), 3.89 (s, 3H), 3.83 (bs, 2H).

^{13}C NMR (101 MHz, $CDCl_3$) δ 155.64, 148.54, 147.23, 134.36, 127.54, 114.47, 111.68, 102.32, 57.62, 55.70.

3-(4-Amino-3-chlorophenyl)-5-methoxy-1,3,4-oxadiazol-2(3*H*)-one (67d)

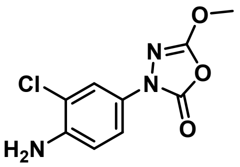

A solution of **66d** (24 mg, 88 µmol) in dioxane (2 mL) was stirred with Raney nickel (±5 mg, 50% slurry in H_2O) under hydrogen atmosphere at room pressure for 2 h. Afterwards the reaction mixture flushed with nitrogen and was then filtered through a Celite® filter. The filtrate was concentrated *in vacuo* to give the title compound as a red solid (19 mg, 79 µmol, 92%).

^{1}H NMR (400 MHz, MeOD) δ 7.58 (d, *J* = 2.5 Hz, 1H), 7.42 (dd, *J* = 8.8, 2.5 Hz, 1H), 6.86 (d, *J* = 8.8 Hz, 1H), 4.09 (s, 3H), 3.66 (s, 2H).

^{13}C NMR (101 MHz, MeOD) δ 157.39, 150.09, 143.84, 128.23, 121.01, 119.78, 119.48, 116.73, 58.54.

2-Amino-5-(5-methoxy-2-oxo-1,3,4-oxadiazol-3(2*H*)-yl)benzonitrile (67e)

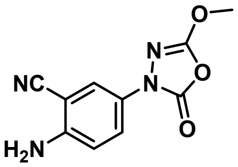

66e (26 mg, 99 µmol) was dissolved in DCM (3 mL) and MeOH (3 mL), and was reacted with Pd/C catalyst (10 wt%, 8.0 mg, 7.5 µmol) following General Procedure F. Purification of the crude material by column chromatography (0% → 0.25% MeOH in DCM) gave the title compound as a clear white solid (6.0 mg, 26 µmol, 26%).

^{1}H NMR (400 MHz, $CDCl_3$) δ 7.85 (dd, *J* = 9.0, 2.6 Hz, 1H), 7.80 (d, *J* = 2.6 Hz, 1H), 6.83 (d, *J* = 9.0 Hz, 1H), 4.47 (bs, 2H), 4.13 (s, 3H).

^{13}C NMR (101 MHz, $CDCl_3$) δ 156.03, 148.27, 147.50, 127.36, 124.66, 121.87, 116.88, 115.95, 96.25, 57.93.

3-(4-Amino-3-methylphenyl)-5-ethoxy-1,3,4-oxadiazol-2(3*H*)-one (67f)

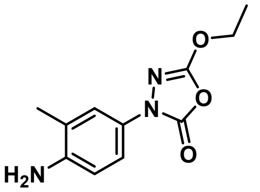

66f (420 mg, 1.58 mmol) was dissolved in DCM (25 mL) and MeOH (15 mL), and reacted with Pd/C catalyst (20 wt%, 150 mg, 0.28 mmol) following General Procedure F. The title compound was obtained as an off-white solid (223 mg, 0.950 mmol, 60%) without further need for purification.

^{1}H NMR (400 MHz, $CDCl_3$) δ 7.40 (d, *J* = 2.6 Hz, 1H), 7.36 (dd, *J* = 8.5, 2.6 Hz, 1H), 6.67 (d, *J* = 8.5 Hz, 1H), 4.42 (q, *J* = 7.1 Hz, 2H), 3.65 (s, 2H), 2.17 (s, 3H), 1.46 (t, *J* = 7.1 Hz, 3H).

^{13}C NMR (101 MHz, $CDCl_3$) δ 155.02, 148.69, 142.95, 127.44, 122.94, 121.35, 118.10, 115.00, 67.56, 17.57, 14.19.

3-(4-Amino-3-methylphenyl)-5-isobutoxy-1,3,4-oxadiazol-2(3*H*)-one (67g)

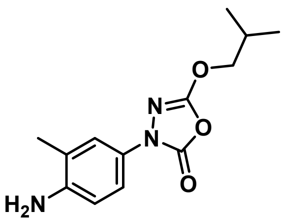

66g (673 mg, 2.30 mmol) was dissolved in DCM (25 mL) and MeOH (15 mL), and was reacted with Pd/C catalyst (20 wt%, 70.0 mg, 0.13 mmol) following General Procedure F. The title compound was obtained as an off-white solid (105 mg, 0.400 mmol, 17%) without further need for purification.

^{1}H NMR (400 MHz, $CDCl_3$) δ 7.42 (d, *J* = 2.6 Hz, 1H), 7.38 (dd, *J* = 8.5, 2.6 Hz, 1H), 6.68 (d, *J* = 8.5 Hz, 1H), 4.13 (d, *J* = 6.6 Hz, 2H), 3.65 (s, 2H), 2.19 (s, 3H), 2.17 – 2.08 (m, 1H), 1.03 (d, *J* = 6.8 Hz, 6H).

^{13}C NMR (101 MHz, $CDCl_3$) δ 155.34, 148.73, 142.93, 127.55, 123.00, 121.43, 118.18, 115.07, 77.26, 27.84, 18.81, 17.62.

3-(4-Amino-3-methylphenyl)-5-butoxy-1,3,4-oxadiazol-2(3*H*)-one (67h)

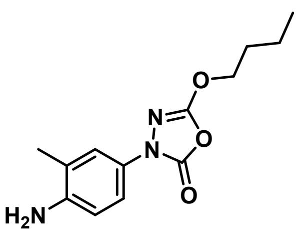

66h (565 mg, 1.93 mmol) was dissolved in DCM (25 mL) and MeOH (15 mL), and reacted with Pd/C catalyst (5 wt%, 210 mg, 99 µmol) following General Procedure F. The title compound was obtained as an off-white solid (163 mg, 0.620 mmol, 32%) without further need for purification.

^{1}H NMR (400 MHz, $CDCl_3$) δ 7.41 (d, *J* = 2.6 Hz, 1H), 7.37 (dd, *J* = 8.5, 2.6 Hz, 1H), 6.68 (d, *J* = 8.5 Hz, 1H), 4.36 (t, *J* = 6.5 Hz, 2H), 3.65 (s, 2H), 2.18 (s, 3H), 1.80 (quint, *J* = 6.5 Hz, 2H), 1.48 (sext, *J* = 7.4 Hz, 2H), 0.98 (t, *J* = 7.4 Hz, 3H).

^{13}C NMR (101 MHz, $CDCl_3$) δ 155.22, 148.72, 142.93, 127.50, 122.97, 121.39, 118.14, 115.03, 71.33, 30.47, 18.84, 17.59, 13.66.

3-(4-Amino-3-methylphenyl)-5-(2-methoxyethoxy)-1,3,4-oxadiazol-2(3*H*)-one (67i)

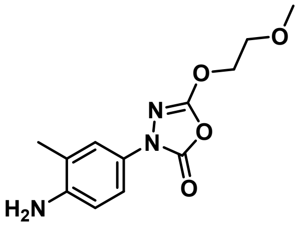

66i (500 mg, 1.69 mmol) was dissolved in DCM (15 mL) and MeOH (15 mL), and reacted with Pd/C catalyst (5 wt%, 360 mg, 0.17 mmol) following General Procedure F. The title compound was obtained as an off-white solid (440 mg, 1.66 mmol, 98%) without further need for purification.

^{1}H NMR (400 MHz, $CDCl_3$) δ 7.40 (d, *J* = 2.5, 1H), 7.37 (dd, *J* = 8.5, 2.6 Hz, 1H), 6.69 (d, *J* = 8.5 Hz, 1H), 4.53 – 4.46 (m, 2H), 3.86 – 3.64 (m, 4H), 3.43 (s, 3H), 2.19 (s, 3H).

^{13}C NMR (101 MHz, $CDCl_3$) δ 155.08, 148.60, 142.68, 127.53, 123.14, 121.37, 118.10, 115.17, 70.27, 69.66, 59.24, 17.58.

Methyl 2-(*m*-tolyl)hydrazine-1-carboxylate (70)

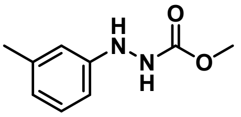

m-Tolylhydrazine hydrochloride (801 mg, 5.05 mmol) and pyridine (2.00 mL, 25.0 mmol) and were dissolved in dry DCM (20 mL). The mixture was cooled to 0°C, after which methyl chloroformate (430 µL, 5.55 mmol) was added dropwise. The mixture was stirred for 2 h , after which the reaction mixture was diluted with H_2O (20 mL), and subsequently extracted with Et_2O (100 mL). The organic layer was concentrated to about 10 mL, and subsequently cooled at to 0°C, forming crystals. Using filtration, crystals were collected yielding the title compound as a yellow solid (546 mg, 3.03 mmol, 60%).

^{1}H NMR (400 MHz, $CDCl_3$) δ 7.10 (t, 1H, *J* = 7.5 Hz), 6.72 (bs, 1H), 6.70 (d, *J* = 7.5 Hz, 1H), 6.64 – 6.56 (m, 2H), 5.21 (bs, 1H), 3.73 (s, 3H), 2.28 (s, 3H).

^{13}C NMR (101 MHz, $CDCl_3$) δ 157.78, 147.96, 139.18, 129.14, 121.97, 113.82, 110.27, 52.93, 21.62.

Methyl 2-(4-amino-3-methylphenyl)hydrazine-1-carboxylate (71)

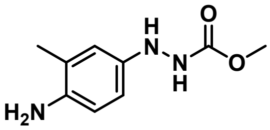

65a (100 mg, 0.44 mmol) was dissolved in DCM (5 mL) and MeOH (5 mL), and was reacted with Pd/C catalyst (10 wt%, 50 mg, 47 µmol) following General Procedure F. Purification of the crude material by column chromatography (0% → 2% MeOH in DCM) was performed to give the title compound as a yellowish solid (54 mg, 0.28 mmol, 62%).

^{1}H NMR (300 MHz, $CDCl_3$) δ 6.92 (bs, 1H), 6.58 – 6.46 (m, 3H), 5.55 (bs, 1H), 3.71 (s, 3H), 3.28 (bs, 2H), 2.09 (s, 3H).

^{13}C NMR (75 MHz, $CDCl_3$) δ 140.37, 138.93, 123.84, 116.54, 116.08, 112.73, 52.73, 17.62.

3-Bromo-*N*-(4-(5-methoxy-2-oxo-1,3,4-oxadiazol-3(2*H*)-yl)-2-methylphenyl)benzamide (73)

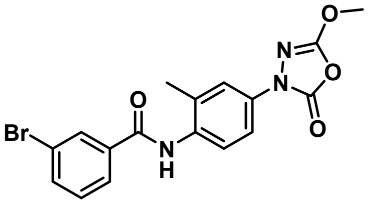

2-Bromobenzoic acid (182 mg, 0.90 mmol) was reacted with **12** (100 mg, 0.45 mmol) following General Procedure D. The crude product was purified with column chromatography (0% → 2% MeOH in DCM) to obtain title compound **73** as a yellow solid (78 mg, 0.45 mmol, 43%).

^{1}H NMR (400 MHz, DMSO) δ 10.08 (s, 1H), 8.16 (t, *J* = 1.9 Hz, 1H), 7.98 (dt, *J* = 7.8, 1.3 Hz, 1H), 7.81 (ddd, *J* = 8.0, 2.1, 1.0 Hz, 1H), 7.63 – 7.55 (m, 2H), 7.51 (t, *J* = 7.9 Hz, 1H), 7.44 (d, *J* = 8.6 Hz, 1H), 4.08 (s, 3H), 2.28 (s, 3H).

^{13}C NMR (101 MHz, DMSO) δ 163.98, 155.43, 148.03, 136.54, 135.00, 134.38, 133.84, 133.39, 130.75, 130.34, 127.47, 126.82, 121.77, 119.28, 115.51, 58.15, 18.16.

Methyl 3-ethynylbenzoate (75)

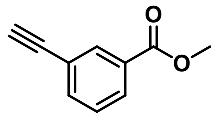

3-Ethynyl benzoic acid (200 mg, 1.37 mmol) and K_2CO_3 (567 mg, 4.11 mmol) were dissolved in DMF (10 mL) followed by cooling on ice. Methyl iodide (0.171 mL, 2.74 mmol) was then added dropwise, and the mixture was stirred overnight. The reaction mixture was diluted with EtOAc and washed with H_2O (3×), dried (Na_2SO_4), filtered and concentrated *in vacuo* to yield the title compound as a yellow oil (210 mg, 1.37 mmol, 96%).

^{1}H NMR (300 MHz, $CDCl_3$) δ 8.17 (t, *J* = 1.7 Hz, 1H), 8.01 (dt, *J* = 7.9, 1.5 Hz, 1H), 7.66 (dt, *J* = 7.7, 1.5 Hz, 1H), 7.40 (t, *J* = 7.8 Hz, 1H), 3.92 (s, 3H), 3.13 (s, 1H).

^{13}C NMR (75 MHz, $CDCl_3$) δ 166.31, 136.31, 133.32, 130.53, 129.87, 128.56, 122.65, 82.64, 78.28, 52.39.

Methyl 3-(phenylethynyl)benzoate (76)

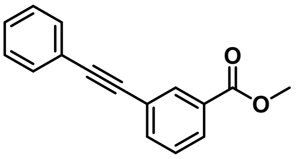

One flask was was charged with methyl 3-ethynylbenzoate (**75**) (100 mg, 0.624 mmol), iodobenzene (0.139 mL, 1.25 mmol), and triethylamine (1 mL), and a second flask was charged with CuI (23.8

mg, 0.125 mmol), PPh_3 (16.4 mg, 62.0 µmol), $Pd(PPh_3)_2Cl_2$ (43.8 mg, 62.0 µmol), and triethylamine (4 mL). Both mixtures were purged with argon while sonicating for 15 min, followed by addition of the second mixture to the first. The reaction mixture was warmed to 40°C and was stirred overnight, after which the solvent was evaporated under nitrogen flow. The residue was resuspended in 1 M aq. HCl and EtOAc, and the resulting aqueous layer was extracted with EtOAc (3×). The combined organic layers were washed with brine, dried ($MgSO_4$), filtered, and concentrated *in vacuo*. Purification of the crude material by column chromatography (5% EtOAc in pentane) yielded the title compound (107 mg, 0.451 mmol, 72%).

^{1}H NMR (400 MHz, $CDCl_3$) δ 8.20 (t, *J* = 1.7 Hz, 1H), 7.98 (dt, *J* = 7.9, 1.5 Hz, 1H), 7.68 (dt, *J* = 7.7, 1.5 Hz, 1H), 7.56 – 7.50 (m, 2H), 7.40 (t, *J* = 7.8 Hz, 1H), 7.36 – 7.30 (m, 3H), 3.91 (s, 3H).

^{13}C NMR (101 MHz, $CDCl_3$) δ 166.43, 135.72, 132.78, 131.72, 130.47, 129.25, 128.61, 128.55, 128.46, 123.79, 122.90, 90.36, 88.38, 52.31.

3-(Phenylethynyl)benzoic acid (77)

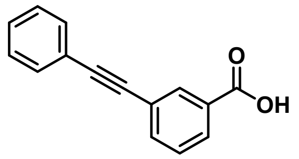

76 (107 mg, 0.451 mmol) was dissolved in MeOH (5 mL) and THF (5 mL) in a 250 mL round-bottom flask, after which KOH (275 mg, 4.90 mmol) was added. The mixture was stirred overnight, after which it was diluted with H_2O (100 mL). Carefully, 50% aq. H_2SO_4 (30 mL) was added, followed by additional stirring (30 min). The precipitate was filtered, washed with H_2O and dried *in vacuo.* Purification by column chromatography afforded **77** as an off-white solid (75.0 mg, 0.337 mmol, 75%).

^{1}H NMR (400 MHz, $CDCl_3$) δ 12.14 (bs, 1H), 8.29 (t, *J* = 1.7 Hz, 1H), 8.07 (dt, *J* = 7.9, 1.5 Hz, 1H), 7.75 (dt, *J* = 7.7, 1.5 Hz, 1H), 7.55 (dd, *J* = 6.6, 3.0 Hz, 2H), 7.45 (t, *J* = 7.8 Hz, 1H), 7.39 – 7.32 (m, 3H).

^{13}C NMR (101 MHz, $CDCl_3$) δ 171.47, 136.64, 133.42, 131.77, 129.84, 129.64, 128.72, 128.69, 128.49, 124.05, 122.84, 90.59, 88.16.

Ethyl 3-(pyridin-3-yloxy)benzoate (79)

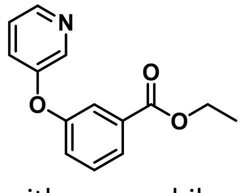

A flask containing pyridin-3-ol (114 mg, 1.2 mmol), picolinic acid (25 mg, 0.20 mmol), K_3PO_4 (425 mg, 2.0 mmol), and CuI (19 mg, 0.10 mmol) was purged with argon and subsequently DMSO (2 mL) and ethyl 3-iodobenzoate (168 µL, 1.0 mmol) were added. The solution was purged with argon while sonicating for 15 min, after which the reaction mixture was stirred vigorously at 110°C overnight. EtOAc (10 mL) and H_2O (1 mL) were added and the mixture was stirred for 3 min. The aqueous layer was extracted with EtOAc (2×), and the combined organic layers were dried ($MgSO_4$), filtered and concentrated *in vacuo*. The resulting crude material was

purified by column chromatography (10% → 30% EtOAc in pentane) to obtain **79** as a yellow oil (88 mg, 0.36 mmol, 36%).

^{1}H NMR (400 MHz, $CDCl_3$) δ 8.42 (dd, *J* = 2.6, 1.0 Hz, 1H), 8.41 – 8.38 (m, 1H), 7.85 (ddd, *J* = 7.8, 1.6, 1.1 Hz, 1H), 7.69 (dd, *J* = 2.6, 1.5 Hz, 1H), 7.44 (t, *J* = 8.2 Hz, 1H), 7.31 – 7.28 (m, 2H), 7.23 (ddd, *J* = 8.2, 2.6, 1.1 Hz, 1H), 4.37 (q, *J* = 7.1 Hz, 2H), 1.38 (t, *J* = 7.1 Hz, 3H).

^{13}C NMR (101 MHz, $CDCl_3$) δ 165.76, 156.50, 153.49, 144.86, 141.56, 132.63, 130.07, 125.68, 125.20, 124.25, 123.37, 119.78, 61.32, 14.31.

3-(Pyridin-3-yloxy)benzoic acid (80)

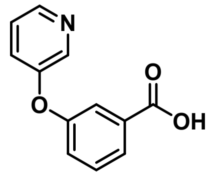

79 (88 mg, 0.36 mmol) was dissolved in 6 M aq. HCl (4 mL) and was stirred overnight at 110°C in a capped microwave vial. The mixture was washed with EtOAc (2×), and the aqueous layer was concentrated *in vacuo* to obtain the hydrochloride salt of **80** as a white powder (81 mg, 0.32 mmol, 89%).

^{1}H NMR (400 MHz, MeOD) δ 8.78 – 8.67 (m, 2H), 8.27 (dd, *J* = 8.8, 2.5 Hz, 1H), 8.14 (dd, *J* = 8.8, 5.5 Hz, 1H), 8.00 – 7.93 (m, 1H), 7.82 (t, *J* = 1.8 Hz, 1H), 7.65 (t, *J* = 7.9 Hz, 1H), 7.52 (dd, *J* = 8.1, 2.5 Hz, 1H).

^{13}C NMR (101 MHz, MeOD) δ 168.06, 158.20, 155.39, 137.36, 135.84, 134.75, 133.32, 132.19, 129.97, 128.46, 125.73, 122.10.

Methyl 3-(pyridin-2-yloxy)benzoate (82)

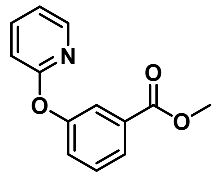

A flask containing 2-bromopyridine (1.9 mL, 20 mmol), picolinic acid (0.49 g, 4.0 mmol), K_3PO_4 (8.5 g, 40 mmol), and CuI (0.38 g, 2.0 mmol) was purged with argon and subsequently DMSO (40 mL) and methyl 3-hydroxybenzoate (3.7 g, 24 mmol) were added. The solution was purged with argon while sonicating for 15 min, and the reaction mixture was stirred vigorously at 110°C overnight. After the reaction was completed, 2 mL of the mixture (5%, 1.0 mmol max.) was taken for further purification. EtOAc (10 mL) and H_2O (1 mL) were added and the aqueous layer was extracted with EtOAc (2×), and the combined organic layers were dried ($MgSO_4$), filtered and concentrated *in vacuo*. The resulting crude material was purified by column chromatography (0% → 2% MeOH in DCM) to obtain **82** as a yellow oil (65 mg, 0.28 mmol, 28%).

^{1}H NMR (400 MHz, $CDCl_3$) δ 8.14 – 8.06 (m, 1H), 7.86 – 7.74 (m, 2H), 7.67 – 7.55 (m, 1H), 7.54 – 7.25 (m, 2H), 6.97 – 6.83 (m, 2H), 3.81 (m, 3H).

^{13}C NMR (101 MHz, $CDCl_3$) δ 166.07, 162.98, 153.92, 147.27, 139.48, 131.49, 129.41, 125.68, 125.50, 122.05, 118.67, 111.55, 51.93.

3-(Pyridin-2-yloxy)benzoic acid (83)

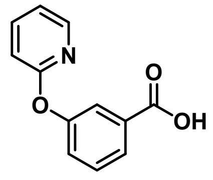

82 (65 mg, 0.28 mmol) was dissolved in 6 M aq. HCl (6 mL) and was stirred overnight at 110°C in a capped microwave vial. The mixture was washed with EtOAc (2×), and the aqueous layer was concentrated *in vacuo* to obtain the hydrochloride salt of **83** (81 mg, 0.32 mmol, 89%) as a white solid.

^{1}H NMR (300 MHz, MeOD) δ 8.59 (d, *J* = 5.6 Hz, 1H), 8.51 (t, *J* = 8.1 Hz, 1H), 7.93 (d, *J* = 7.3 Hz, 1H), 7.82 (s, 1H), 7.78 – 7.57 (m, 3H), 7.16 (d, *J* = 8.6 Hz, 1H).

^{13}C NMR (75 MHz, $CDCl_3$) δ 168.21, 160.46, 152.52, 150.42, 141.08, 133.99, 132.44, 129.56, 126.64, 122.59, 121.65, 113.26.

Methyl 3-(4-(trifluoromethyl)phenoxy)benzoate (85a)

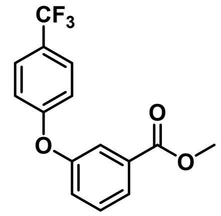

(4-(Trifluoromethyl)phenyl)boronic acid (380 mg, 2.0 mmol) was subjected to General Procedure G to obtain **85a** as a yellow oil (193 mg, 0.65 mmol, 33%).

^{1}H NMR (500 MHz, $CDCl_3$) δ 7.87 (ddd, *J* = 7.8, 1.6, 1.1 Hz, 1H), 7.73 – 7.69 (m, 1H), 7.60 – 7.56 (m, 2H), 7.45 (t, *J* = 7.9 Hz, 1H), 7.25 (ddd, *J* = 8.2, 2.6, 1.1 Hz, 1H), 7.07 – 7.01 (m, 2H), 3.90 (s, 3H).

^{13}C NMR (126 MHz, $CDCl_3$) δ 166.30, 160.07, 156.03, 132.40, 130.21, 127.38 (q, *J* = 3.8 Hz), 125.63, 125.49 (q, *J* = 32 Hz), 124.41, 124.20 (q, *J* = 273 Hz) 120.74, 118.23, 52.37.

Methyl 3-(4-chlorophenoxy)benzoate (85b)

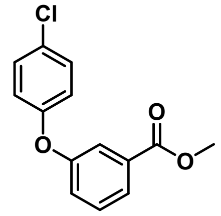

(4-Chlorophenyl)boronic acid (313 mg, 2.0 mmol) was subjected to General Procedure G to obtain **85b** as a clear oil (164 mg, 0.62 mmol, 31%).

^{1}H NMR (400 MHz, $CDCl_3$) δ 7.79 (dt, *J* = 7.8, 1.3 Hz, 1H), 7.63 (dd, *J* = 2.6, 1.5 Hz, 1H), 7.40 (t, *J* = 8.0 Hz, 1H), 7.34 – 7.25 (m, 2H), 7.19 (ddd, *J* = 8.2, 2.6, 1.1 Hz, 1H), 6.98 – 6.89 (m, 2H), 3.88 (s, 3H).

^{13}C NMR (101 MHz, $CDCl_3$) δ 166.38, 157.12, 155.40, 132.07, 132.06 129.96, 128.83, 124.73, 123.38, 120.36, 119.55, 52.33.

Methyl 3-(*p*-tolyloxy)benzoate (85c)

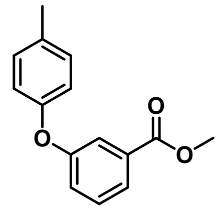

p-Tolylboronic acid (272 mg, 2.0 mmol) was subjected to General Procedure G to obtain **85c** as a clear oil (165 mg, 0.68 mmol, 34%).

^{1}H NMR (400 MHz, $CDCl_3$) δ 7.73 (dt, *J* = 7.8, 1.4 Hz, 1H), 7.63 (dd, *J* = 2.7, 1.5 Hz, 1H), 7.35 (t, *J* = 8.0 Hz, 1H), 7.19 – 7.10 (m, 3H), 6.93 – 6.88 (m, 2H), 3.86 (s, 3H), 2.32 (s, 3H).

Methyl 3-(4-methoxyphenoxy)benzoate (85d)

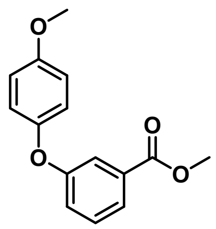

(4-Methoxyphenyl)boronic acid (304 mg, 2.0 mmol) was subjected to General Procedure G to obtain **85d** as a yellow oil (138 mg, 0.53 mmol, 27%).

^{1}H NMR (300 MHz, DMSO) δ 7.71 (ddd, *J* = 7.7, 1.6, 1.1 Hz, 1H), 7.61 – 7.54 (m, 1H), 7.35 (t, *J* = 8.1 Hz, 1H), 7.14 (ddd, *J* = 8.2, 2.6, 1.1 Hz, 1H), 7.05 – 6.79 (m, 4H), 3.87 (s, 3H), 3.80 (s, 3H).

^{13}C NMR (75 MHz, $CDCl_3$) δ 166.68, 158.76, 156.28, 149.62, 131.81, 129.71, 123.63, 122.18, 121.06, 118.24, 115.08, 55.69, 52.28.

Methyl 3-(4-fluorophenoxy)benzoate (85e)

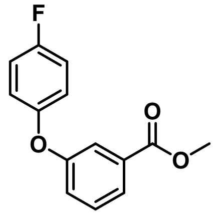

(4-Fluorophenyl)boronic acid (280 mg, 2.0 mmol) was subjected to General Procedure G to obtain **85e** as a clear oil (133 mg, 0.54 mmol, 27%).

^{1}H NMR (400 MHz, $CDCl_3$) δ 7.76 (dt, *J* = 7.7, 1.2 Hz, 1H), 7.60 (dd, *J* = 2.6, 1.5 Hz, 1H), 7.38 (t, *J* = 7.9 Hz, 1H), 7.16 (ddd, *J* = 8.2, 2.6, 1.1 Hz, 1H), 7.09 – 6.93 (m, 4H), 3.88 (s, 3H).

^{13}C NMR (101 MHz, $CDCl_3$) δ 166.49, 159.13 (d, *J* = 243 Hz), 157.92, 152.37 (d, *J* = 2.6 Hz), 131.99, 129.87, 124.27, 122.76, 120.87 (d, *J* = 8.2 Hz), 118.89, 116.59 (d, *J* = 23 Hz), 52.31.

Methyl 3-(3-chlorophenoxy)benzoate (85f)

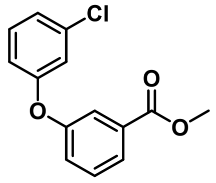

(3-Chlorophenyl)boronic acid (313 mg, 2.0 mmol) was subjected to General Procedure G to obtain **85f** as a clear oil (150 mg, 0.57 mmol, 29%).

^{1}H NMR (400 MHz, $CDCl_3$) δ 7.81 (dt, *J* = 7.8, 1.3 Hz, 1H), 7.67 (dd, *J* = 2.6, 1.6 Hz, 1H), 7.41 (t, *J* = 8.0 Hz, 1H), 7.32 – 7.15 (m, 2H), 7.08 (ddd, *J* = 8.0, 2.0, 1.0 Hz, 1H), 6.98 (t, *J* = 2.2 Hz, 1H), 6.87 (ddd, *J* = 8.3, 2.4, 0.9 Hz, 1H), 3.88 (s, 3H).

^{13}C NMR (101 MHz, $CDCl_3$) δ 166.26, 157.76, 156.55, 135.20, 132.13, 130.68, 129.98, 125.07, 123.79, 123.75, 120.12, 119.09, 116.91, 52.28.

Methyl 3-(*m*-tolyloxy)benzoate (85g)

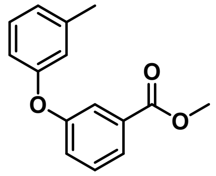

m-Tolylboronic acid (272 mg, 2.0 mmol) was subjected to General Procedure G to obtain **85g** as a clear oil (120 mg, 0.50 mmol, 25%).

^{1}H NMR (400 MHz, $CDCl_3$) δ 7.76 (dt, *J* = 7.7, 1.3 Hz, 1H), 7.65 (dd, *J* = 2.6, 1.5 Hz, 1H), 7.36 (t, *J* = 7.9 Hz, 1H), 7.24 – 7.15 (m, 2H), 7.24 – 7.16 (m, 1H), 6.85 – 6.77 (m, 2H), 3.86 (s, 3H), 2.31 (s, 3H).

^{13}C NMR (101 MHz, $CDCl_3$) δ 166.56, 157.63, 156.69, 140.16, 131.90, 129.74, 129.66, 124.62, 124.21, 123.30, 119.83, 119.56, 116.14, 52.23, 21.41.

Methyl 3-(3-(trifluoromethyl)phenoxy)benzoate (85h)

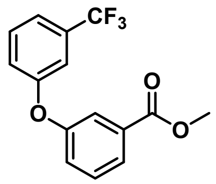

(3-(Trifluoromethyl)phenyl)boronic acid (380 mg, 2.0 mmol) was subjected to General Procedure G to obtain **85h** as a yellow oil (193 mg, 0.65 mmol, 33%).

^{1}H NMR (400 MHz, $CDCl_3$) δ 7.85 (ddd, *J* = 7.7, 1.5, 1.0 Hz, 1H), 7.70 – 7.68 (m, 1H), 7.48 – 7.42 (m, 2H), 7.39 – 7.35 (m, 1H), 7.27 – 7.20 (m, 2H), 7.18 – 7.14 (m, 1H), 3.90 (s, 3H).

^{13}C NMR (101 MHz, $CDCl_3$) δ 166.00, 157.45, 156.49, 132.50 (*J* = 32 Hz), 132.36, 130.60, 130.20, 125.38, 123.95, 123.72 (q, *J* = 273 Hz) 121.88, 120.30, 120.27 (q, *J* = 3.8 Hz), 115.70 (q, *J* = 3.8 Hz), 52.42.

3-(4-(Trifluoromethyl)phenoxy)benzoic acid (86a)

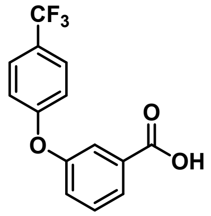

85a (193 mg, 0.65 mmol) was subjected to General Procedure H to obtain **86a** as a white solid (164 mg, 0.65 mmol, 89%).

^{1}H NMR (400 MHz, MeOD) δ 7.85 (dt, *J* = 7.7, 1.3 Hz, 1H), 7.68 – 7.61 (m, 3H), 7.50 (t, *J* = 7.9 Hz, 1H), 7.32 – 7.25 (m, 1H), 7.10 (d, *J* = 8.5 Hz, 2H).

^{13}C NMR (101 MHz, MeOD) δ 168.78, 161.59, 157.35, 134.18, 131.38, 128.42 (q, *J* = 3.8 Hz), 126.74, 126.36 (q, *J* = 32 Hz), 125.63 (q, *J* = 273 Hz), 125.31, 121.60, 119.40.

3-(4-Chlorophenoxy)benzoic acid (86b)

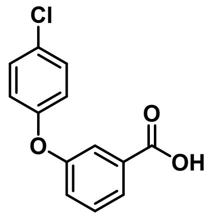

85b (164 mg, 0.63 mmol) was subjected to General Procedure H to obtain **86b** as a white solid (143 mg, 0.58 mmol, 92%).

^{1}H NMR (400 MHz, MeOD) δ 7.78 (d, *J* = 7.7 Hz, 1H), 7.58 (dd, *J* = 2.7, 1.5 Hz, 1H), 7.46 (t, *J* = 7.9 Hz, 1H), 7.40 – 7.31 (m, 2H), 7.29 – 7.25 (m, 1H), 7.03 – 6.95 (m, 2H).

^{13}C NMR (101 MHz, MeOD) δ 168.83, 160.36, 155.36, 133.07, 131.16, 131.02, 129.34, 125.85, 124.24, 121.57, 120.40.

3-(*p*-Tolyloxy)benzoic acid (86c)

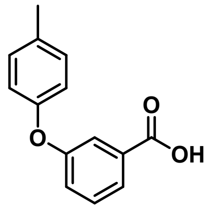

85c (193 mg, 0.80 mmol) was subjected to General Procedure H to obtain **86c** as a white solid (151 mg, 0.66 mmol, 83%).

^{1}H NMR (400 MHz, MeOD) δ 7.72 (dt, *J* = 7.7, 1.3 Hz, 1H), 7.52 (dd, *J* = 2.6, 1.5 Hz, 1H), 7.41 (t, *J* = 8.0 Hz, 1H), 7.22 – 7.13 (m, 3H), 6.94 – 6.86 (m, 2H), 2.33 (s, 3H).

^{13}C NMR (101 MHz, MeOD) δ 169.16, 159.65, 155.48, 134.90, 133.64, 131.51, 130.88, 124.97, 123.55, 120.51, 119.59, 20.76.

3-(4-Methoxyphenoxy)benzoic acid (86d)

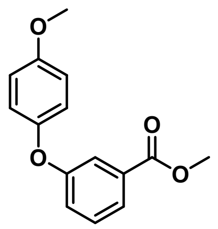

85d (138 mg, 0.53 mmol) was subjected to General Procedure H to obtain **86d** as a white solid (108 mg, 0.44 mmol, 83%).

^{1}H NMR (400 MHz, MeOD) δ 7.69 (dt, *J* = 7.7, 1.3 Hz, 1H), 7.49 (dd, *J* = 2.6, 1.5 Hz, 1H), 7.38 (t, *J* = 8.0 Hz, 1H), 7.13 (ddd, *J* = 8.2, 2.7, 1.1 Hz, 1H), 7.00 – 6.90 (m, 4H), 3.78 (s, 3H).

^{13}C NMR (101 MHz, MeOD) δ 169.22, 160.27, 157.87, 150.79, 133.54, 130.82, 124.63, 122.90, 122.12, 118.91, 116.10, 56.04.

3-(4-Fluorophenoxy)benzoic acid (86e)

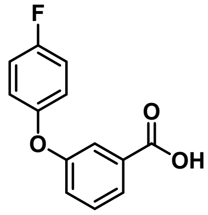

85e (133 mg, 0.54 mmol) was subjected to General Procedure H, with heating to 55°C. **86e** was obtained as a white solid (115 mg, 0.50 mmol, 92%).

^{1}H NMR (400 MHz, MeOD) δ 7.73 (dt, *J* = 7.7, 1.3 Hz, 1H), 7.53 (dd, *J* = 2.6, 1.5 Hz, 1H), 7.40 (t, *J* = 8.0 Hz, 1H), 7.16 (ddd, *J* = 8.2, 2.6, 1.0 Hz, 1H), 7.13 – 6.96 (m, 4H).

^{13}C NMR (101 MHz, MeOD) δ 169.02, 160.5 (d, *J* = 243 Hz), 159.32, 153.76 (d, *J* = 2.6 Hz), 133.72, 131.00, 125.33, 123.55, 122.11 (d, *J* = 8.2 Hz), 119.65, 117.47 (d, *J* = 23 Hz).

3-(3-Chlorophenoxy)benzoic acid (86f)

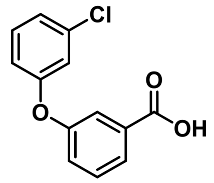

85f (150 mg, 0.57 mmol) was subjected to General Procedure H with heating to 55°C. **86f** was obtained as a white solid (126 mg, 0.51 mmol, 89%).

^{1}H NMR (400 MHz, MeOD) δ 7.80 (dt, *J* = 7.7, 1.2 Hz, 1H), 7.60 (dd, *J* = 2.6, 1.5 Hz, 1H), 7.44 (t, *J* = 7.9 Hz, 1H), 7.30 (t, *J* = 8.2 Hz, 1H), 7.21 (ddd, *J* = 8.2, 2.6, 1.1 Hz, 1H), 7.11 (ddd, *J* = 8.0, 2.0, 0.9 Hz, 1H), 6.99 (t, *J* = 2.2 Hz, 1H), 6.89 (ddd, *J* = 8.3, 2.4, 0.9 Hz, 1H).

^{13}C NMR (101 MHz, MeOD) δ 168.85, 159.14, 158.00, 136.23, 133.95, 132.04, 131.17, 126.19, 124.78, 124.59, 120.86, 120.07, 118.09.

3-(*m*-Tolyloxy)benzoic acid (86g)

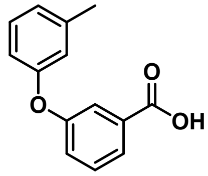

85g (120 mg, 0.50 mmol) was subjected to General Procedure H, with heating to 55°C. **86g** was obtained as a white solid (101 mg, 0.44 mmol, 89%).

^{1}H NMR (400 MHz, MeOD) δ 7.73 (dt, *J* = 7.7, 1.3 Hz, 1H), 7.55 (dd, *J* = 2.6, 1.5 Hz, 1H), 7.39 (t, *J* = 7.9 Hz, 1H), 7.24 – 7.12 (m, 2H), 6.94 (ddt, *J* = 7.5, 1.6, 0.8 Hz, 1H), 6.82 – 6.79 (m, 1H), 6.78 – 6.74 (m, 1H), 2.28 (s, 3H).

^{13}C NMR (101 MHz, MeOD) δ 169.12, 159.15, 157.84, 141.40, 133.60, 130.88, 130.73, 125.73, 125.20, 123.93, 120.90, 120.07, 117.28, 21.39.

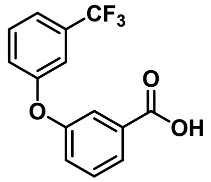

3-(3-(Trifluoromethyl)phenoxy)benzoic acid (86h)

85h (120 mg, 0.41 mmol) was subjected to General Procedure H with heating to 55°C. **86h** was obtained as white solid (105 mg, 0.37 mmol, 92%).

^{1}H NMR (400 MHz, MeOD) δ 7.82 (dt, *J* = 7.7, 1.3 Hz, 1H), 7.62 (dd, *J* = 2.6, 1.5 Hz, 1H), 7.55 – 7.36 (m, 3H), 7.28 – 7.15 (m, 3H).

^{13}C NMR (101 MHz, MeOD) δ 168.81, 158.79, 157.80, 134.09, 133.33 (q, *J* = 32 Hz), 132.01, 131.29, 126.44, 125.08 (q, *J* = 273 Hz) 124.72, 123.13, 121.17 (q, *J* = 3.8 Hz), 121.03, 116.45 (q, *J* = 3.8 Hz).

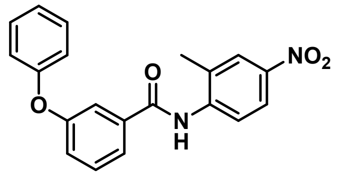

***N*-(2-Methyl-4-nitrophenyl)-3-phenoxybenzamide (88)**

3-Phenoxybenzoic acid (2.0 g, 9.3 mmol) was dissolved in $SOCl_2$ (27 mL) and this mixture was stirred at 60°C overnight. $SOCl_2$ was removed from the reaction mixture by nitrogen flow and the mixture was subsequently co-evaporated with toluene (3×). The crude mixture was redissolved in DCM (30 mL) and K_2CO_3 (2.1 g, 15 mmol) was added. A solution of 2-methyl-4-nitroaniline (1.1 g, 7.5 mmol) in DCM (20 mL) was added dropwise to the stirring reaction mixture. The reaction mixture was stirred for 2 weeks at RT. The mixture was washed with sat. aq. Na_2CO_3 (3×), and the organic layer was dried ($MgSO_4$), filtered and concentrated *in vacuo*. The crude product was purified by column chromatography (6% → 9% EtOAc in pentane) to obtain **88** as a yellow solid (405 mg, 1.2 mmol, 13%).

^{1}H NMR (400 MHz, $CDCl_3$) δ 8.38 (d, *J* = 8.6 Hz, 1H), 8.13 – 8.05 (m, 2H), 7.96 (s, 1H), 7.56 (ddd, *J* = 7.7, 1.7, 1.0 Hz, 1H), 7.52 – 7.42 (m, 2H), 7.42 – 7.33 (m, 2H), 7.25 – 7.12 (m, 2H), 7.08 – 7.00 (m, 2H), 2.40 (s, 3H).

^{13}C NMR (101 MHz, $CDCl_3$) δ 165.02, 158.40, 156.17, 143.71, 141.92, 136.01, 130.55, 130.16, 128.15, 125.77, 124.36, 122.96, 122.38, 121.24, 121.20, 119.58, 117.12, 17.83.

***N*-(4-Amino-2-methylphenyl)-3-phenoxybenzamide (89)**

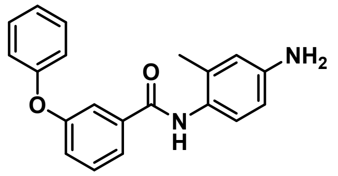

88 (405 mg, 1.2 mmol) was dissolved in MeOH and DCM (14 mL, 1:1), and was reacted with Pd/C catalyst (10 wt%, 70 mg, 66 µmol) following General Procedure F. The resulting crude material was purified by column chromatography (20% → 30% EtOAc in pentane) to obtain title compound **89** (350 mg, 1.1 mmol, 92%) as a brown solid.

^{1}H NMR (300 MHz, MeOD) δ 7.72 – 7.64 (m, 1H), 7.57 (dd, *J* = 2.5, 1.7 Hz, 1H), 7.47 (t, *J* = 7.9 Hz, 1H), 7.42 – 7.30 (m, 2H), 7.21 – 7.08 (m, 2H), 7.08 – 6.95 (m, 3H), 6.65 (d, *J* = 2.5 Hz, 1H), 6.58 (dd, *J* = 8.3, 2.6 Hz, 1H), 2.16 (s, 3H).

^{13}C NMR (75 MHz, MeOD) δ 168.67, 159.17, 158.12, 147.62, 137.74, 136.65, 131.12, 131.06, 128.87, 127.42, 124.92, 123.12, 122.75, 120.25, 118.73, 118.27, 114.50, 18.24.

NMR spectra data key compounds

H NMR compound **1**

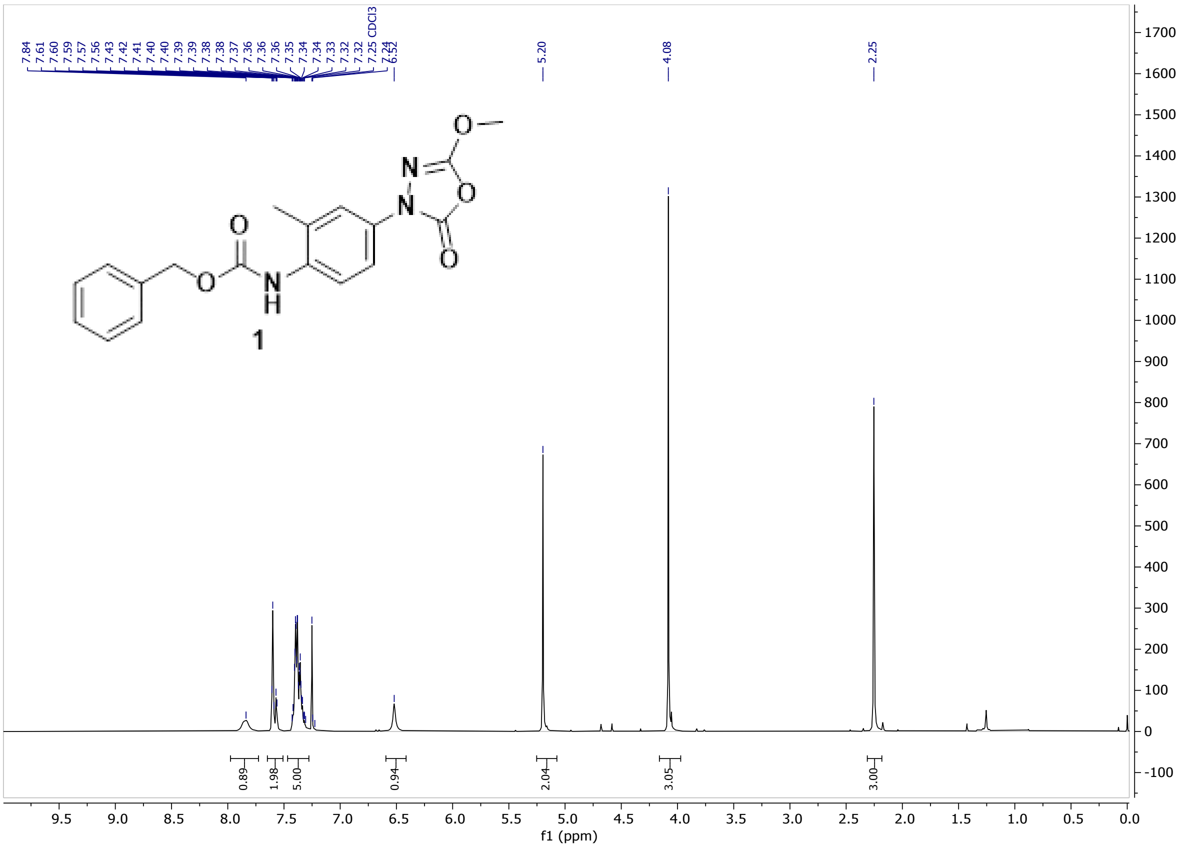

C NMR compound **1**

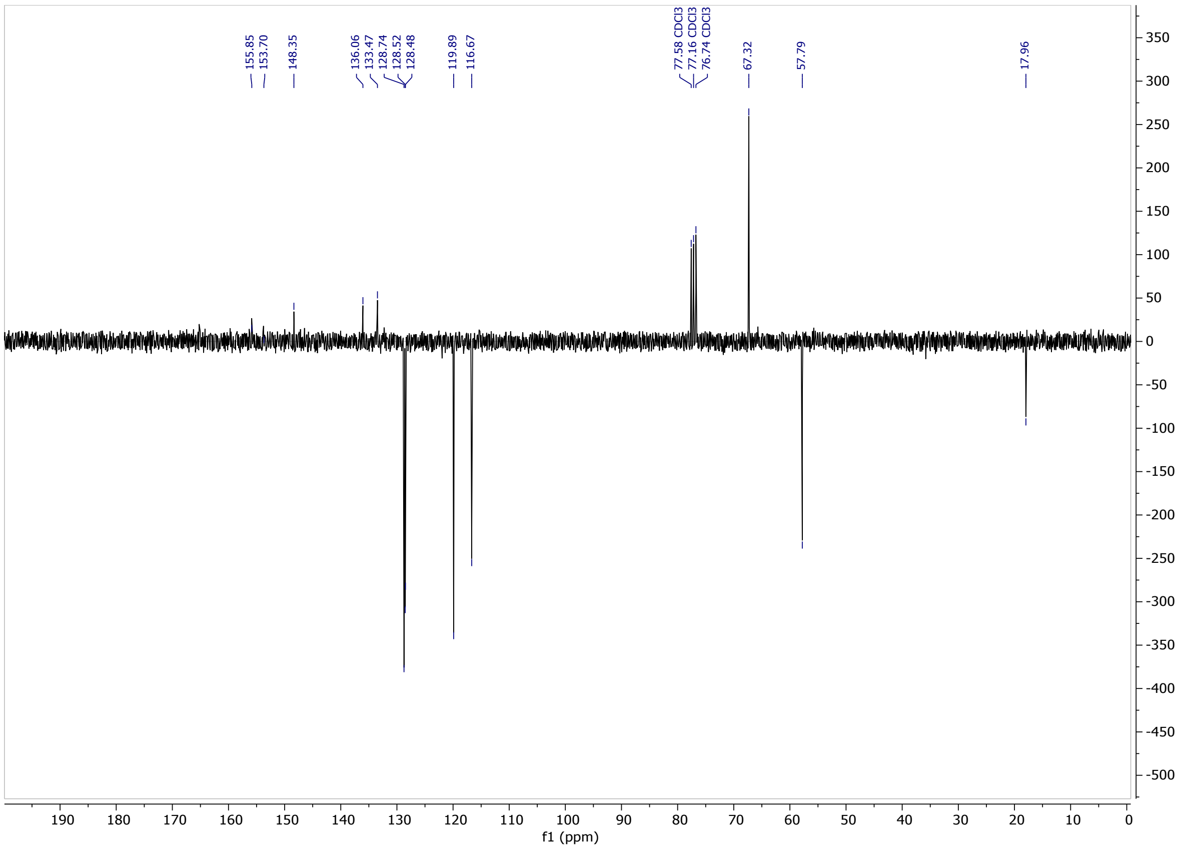

^{1}H NMR compound **2**

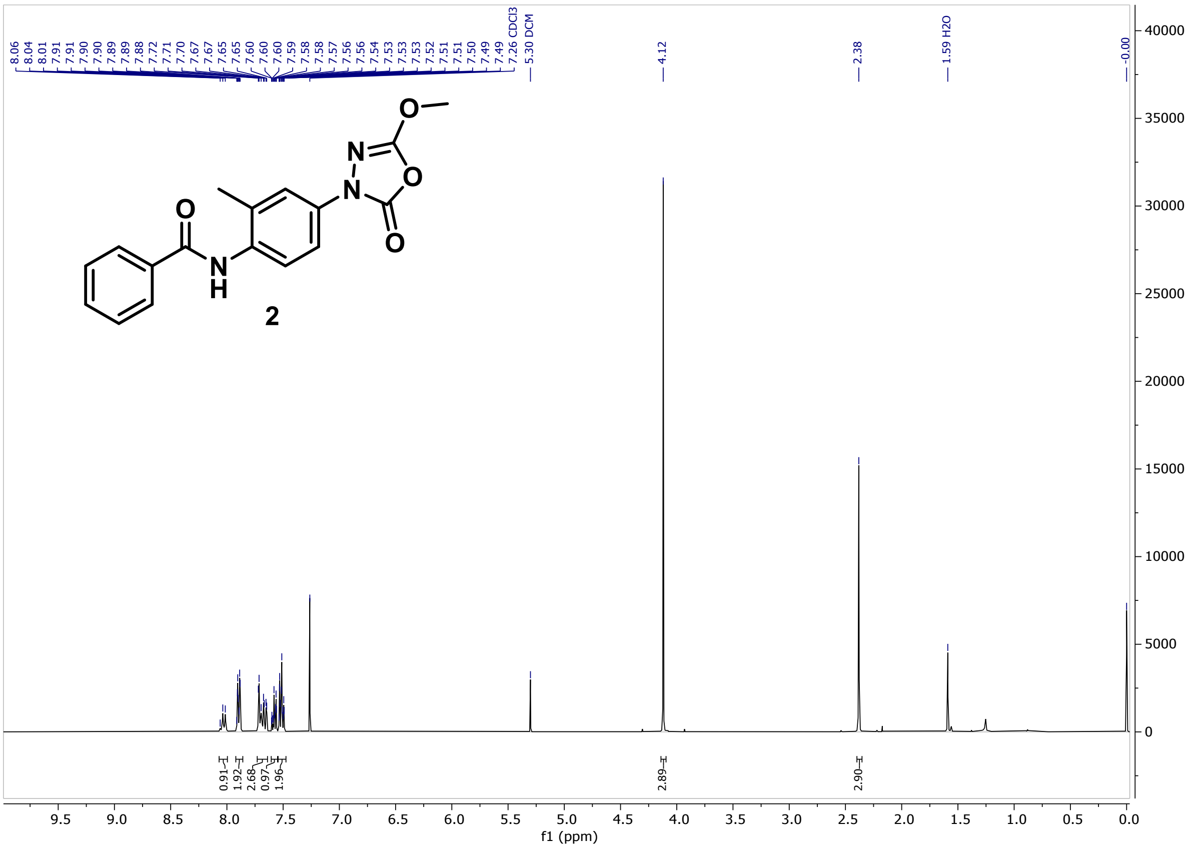

^{13}C NMR compound **2**

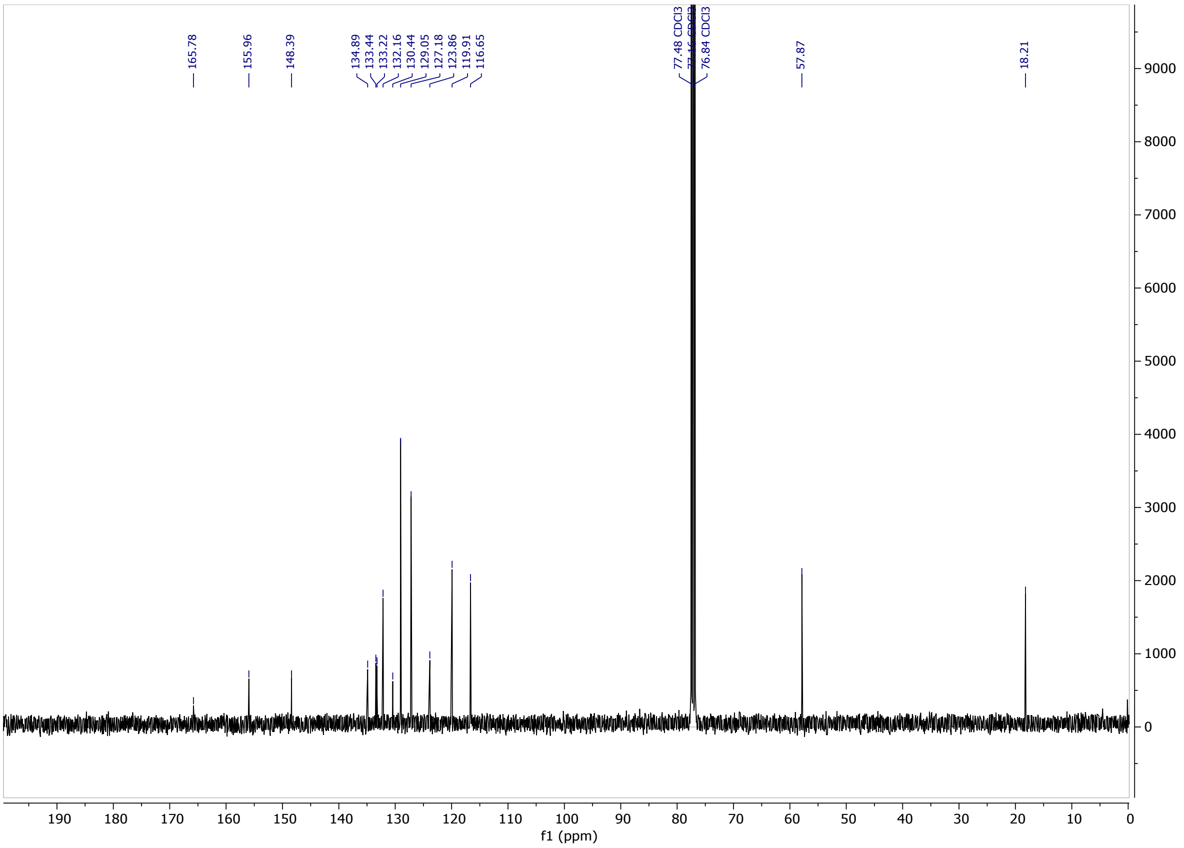

^{1}H NMR compound **3**

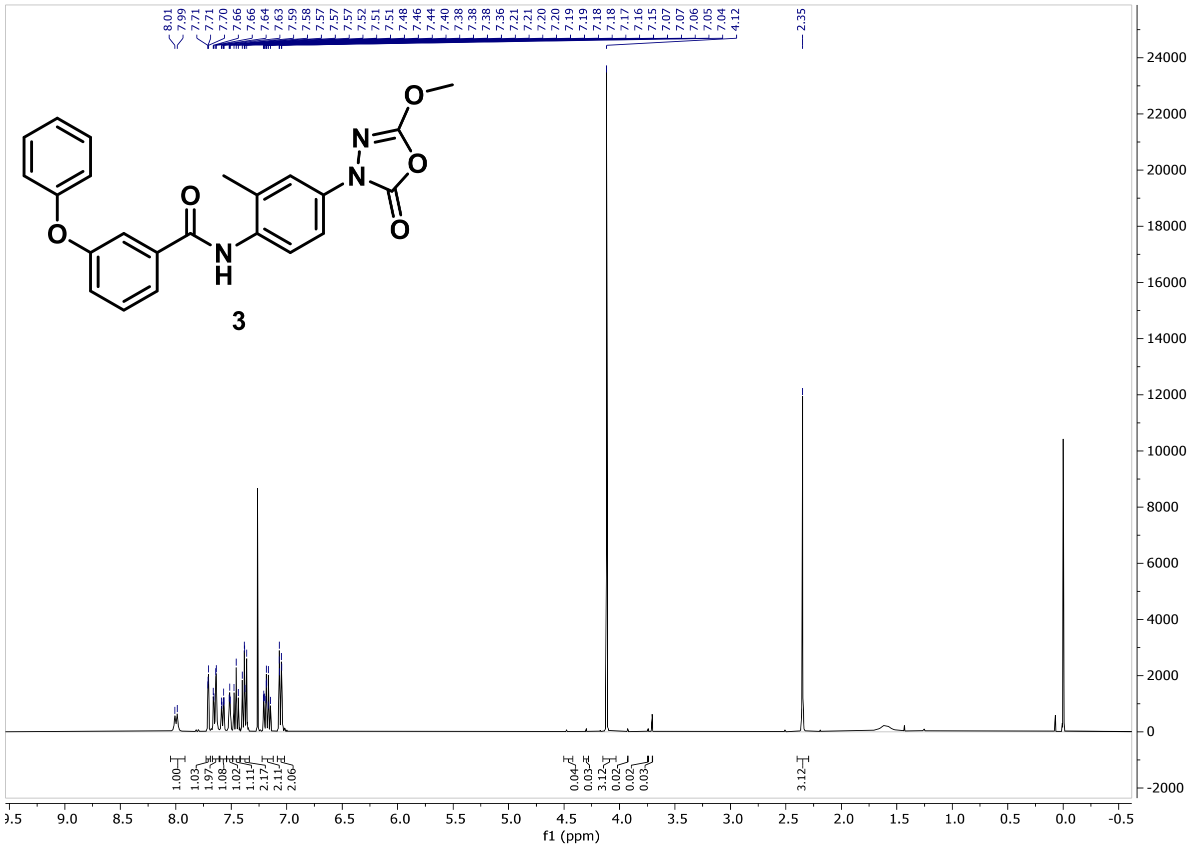

^{13}C NMR compound **3**

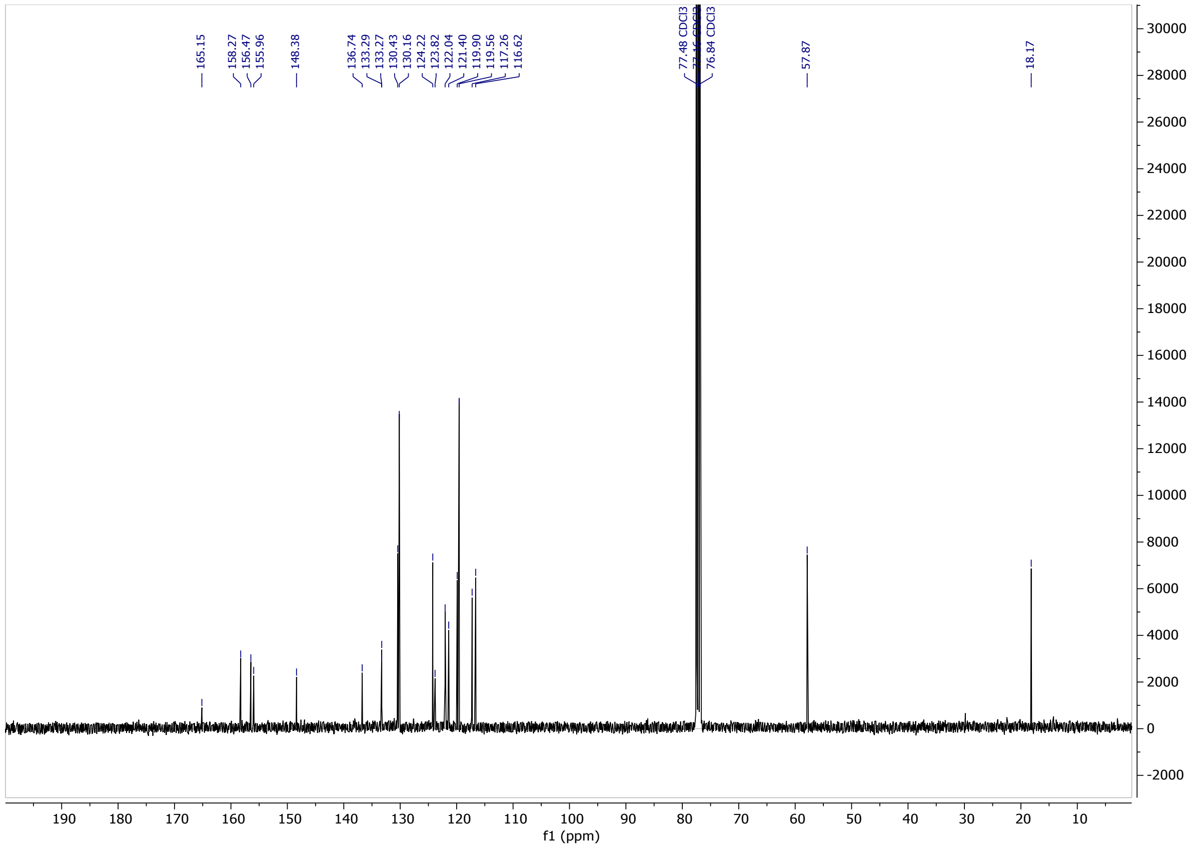

^{1}H NMR compound **4**

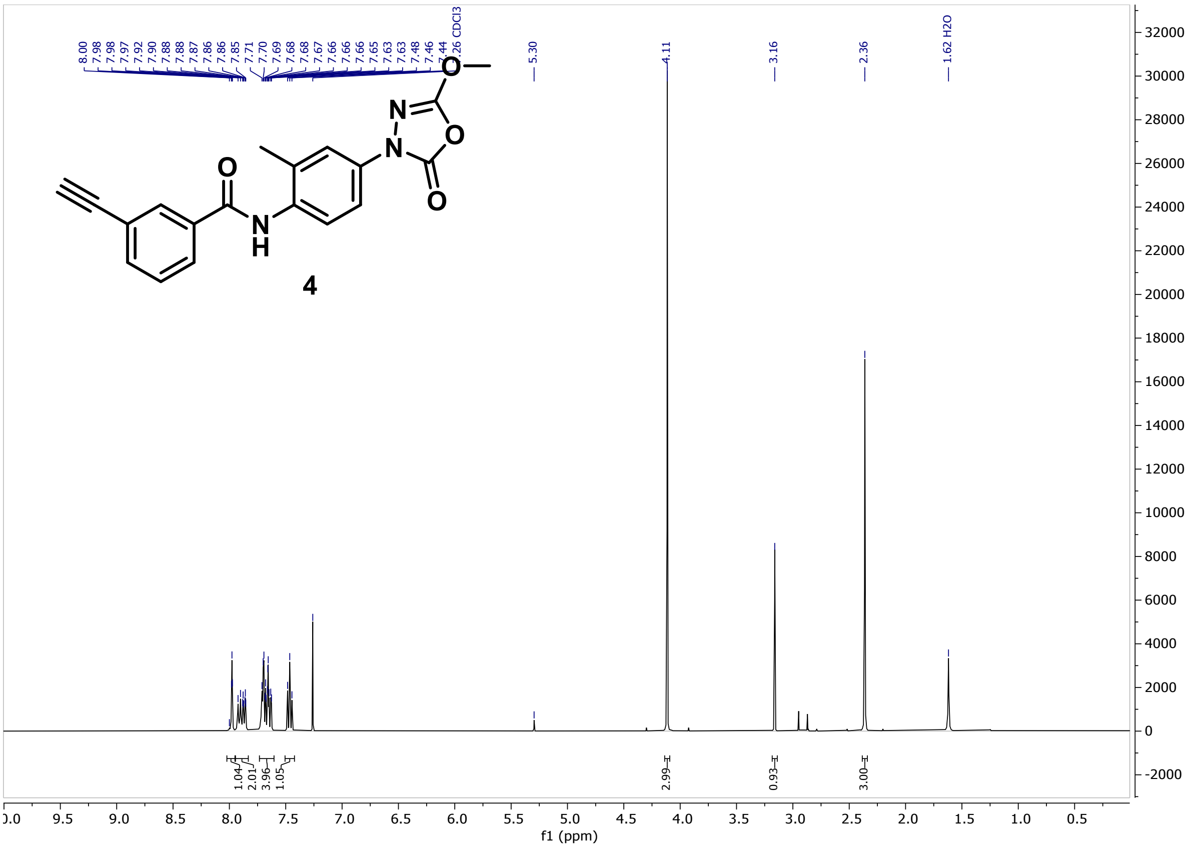

^{13}C NMR compound **4**

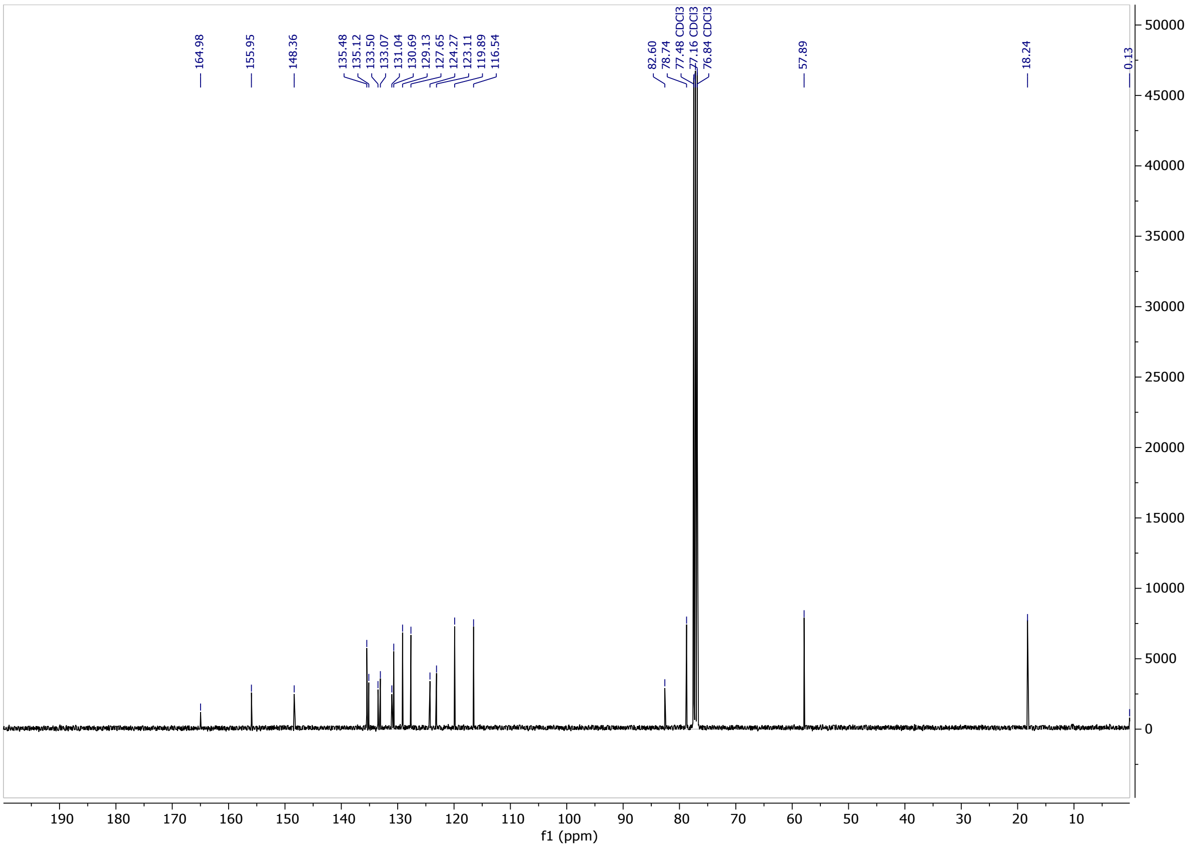

^{1}H NMR compound **5**

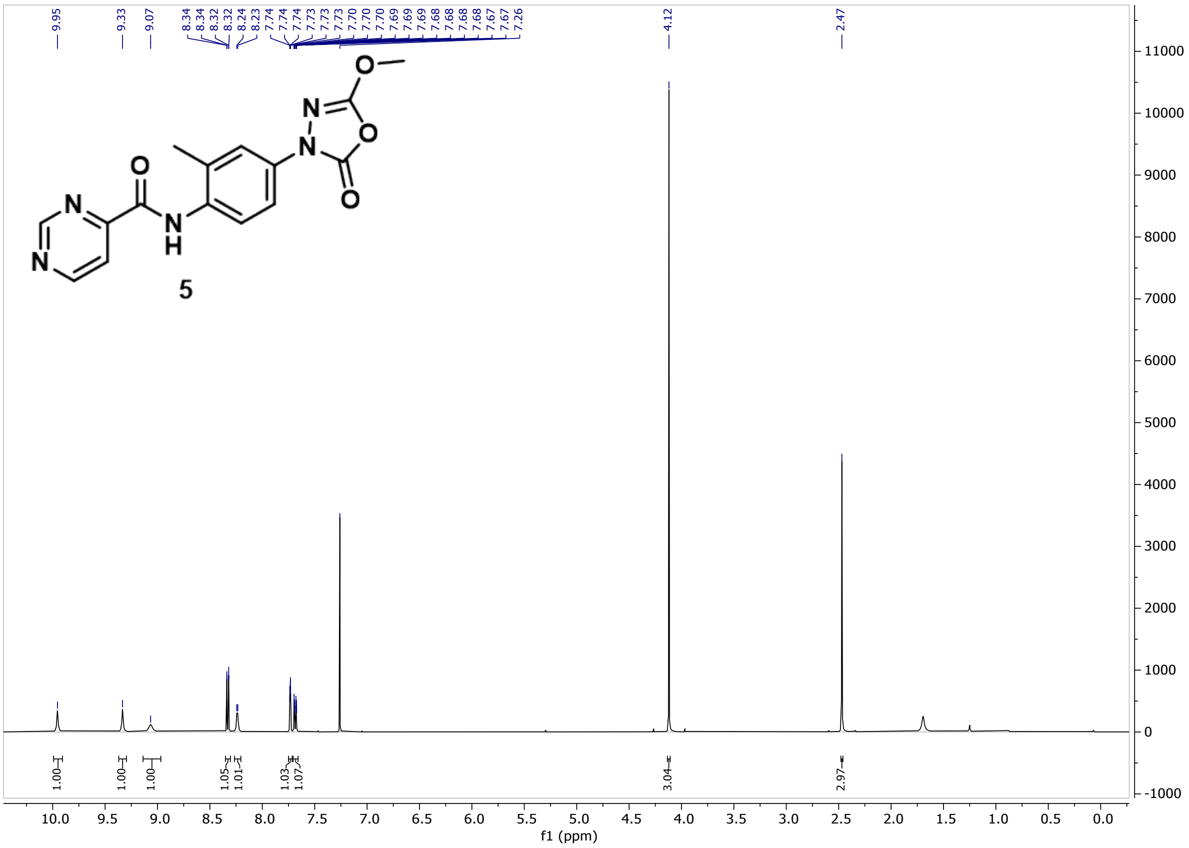

^{13}C NMR compound **5**

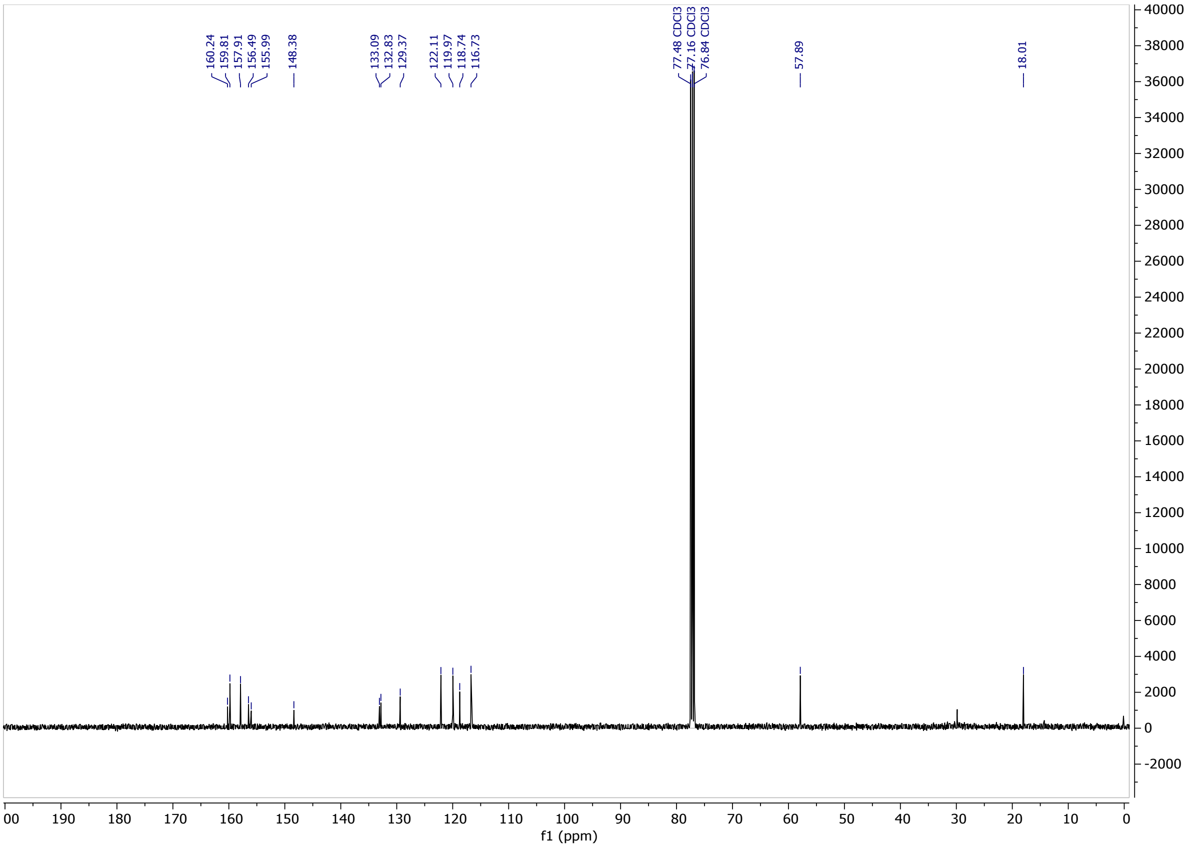

^1H NMR compound **6**

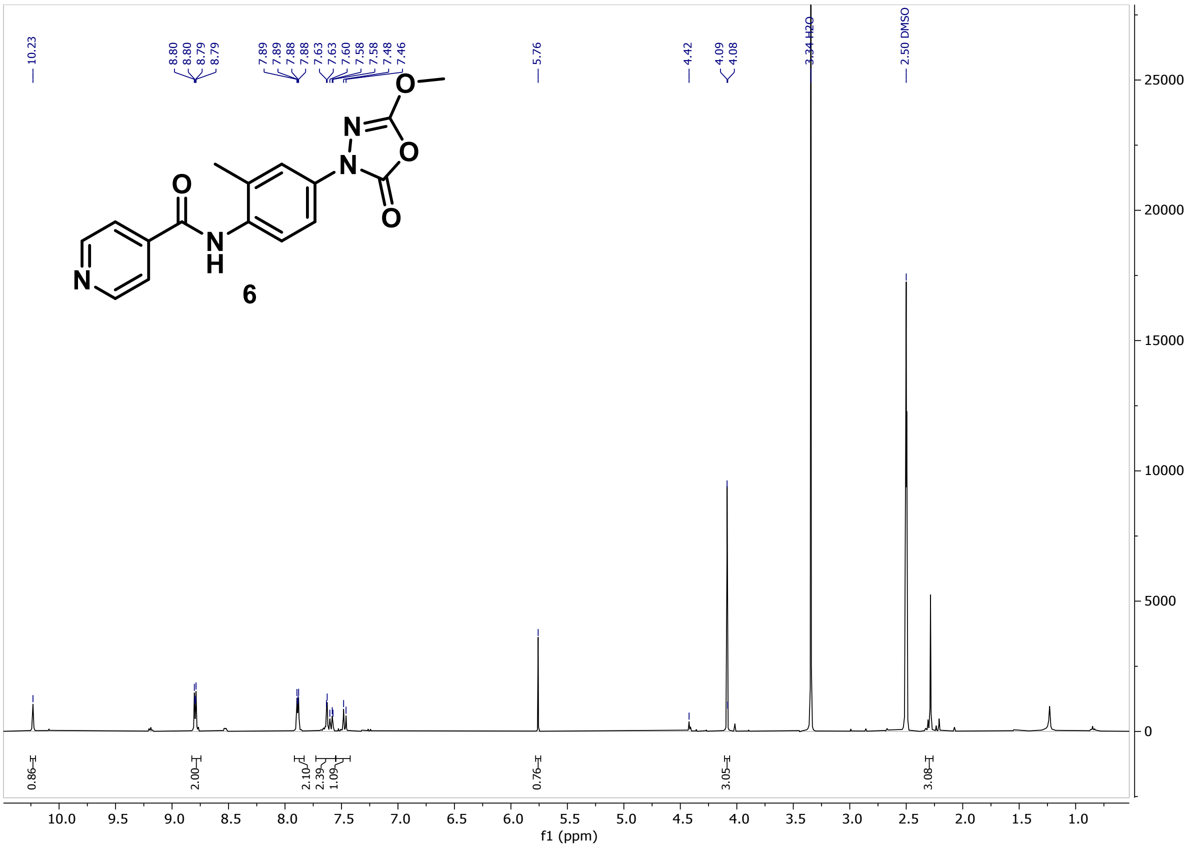

^{13}C NMR compound **6**

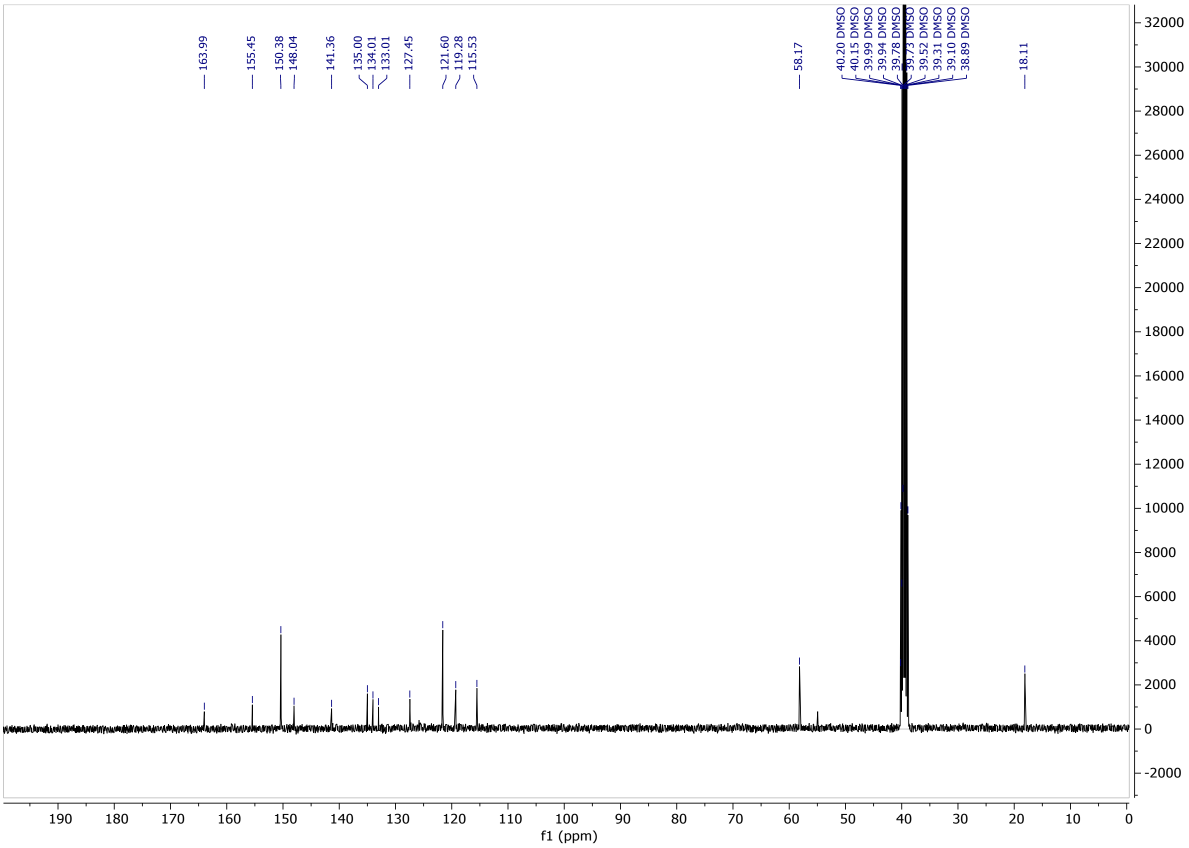

^{1}H NMR compound **7**

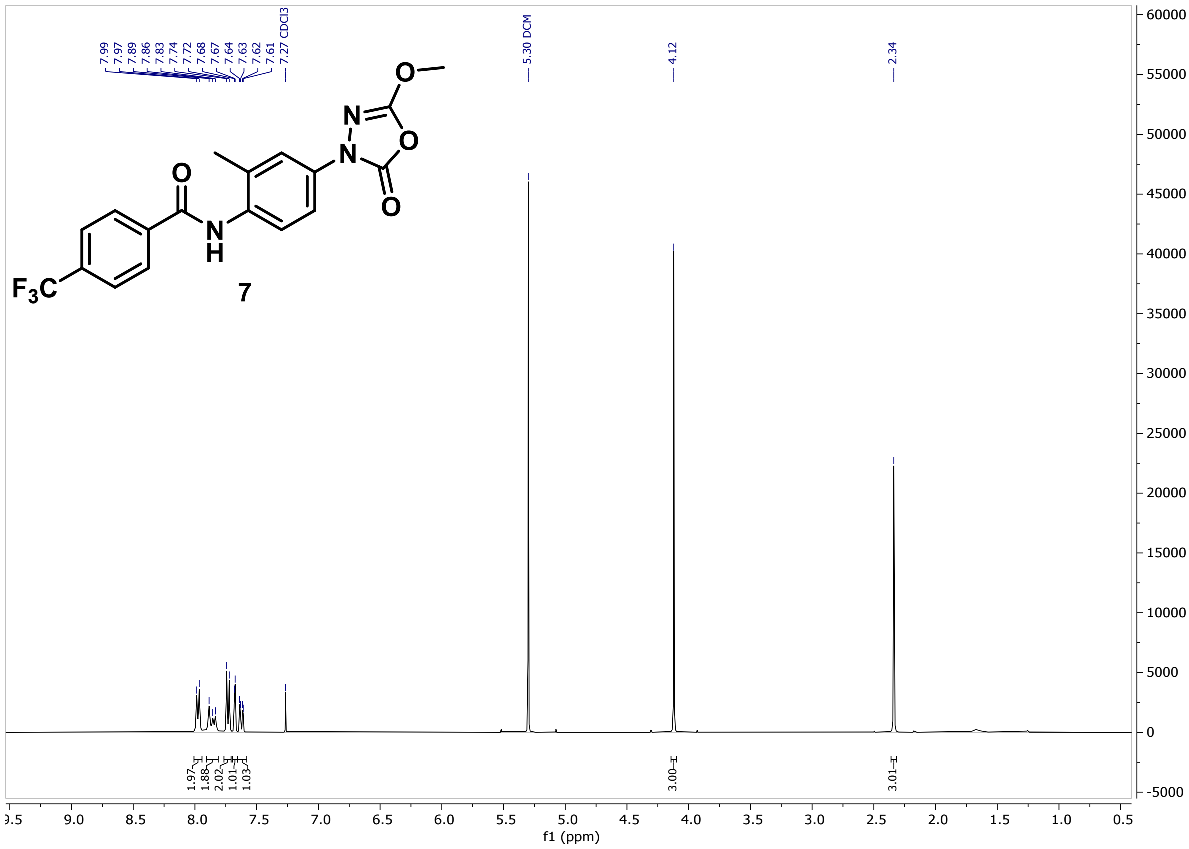

^{13}C NMR compound **7**

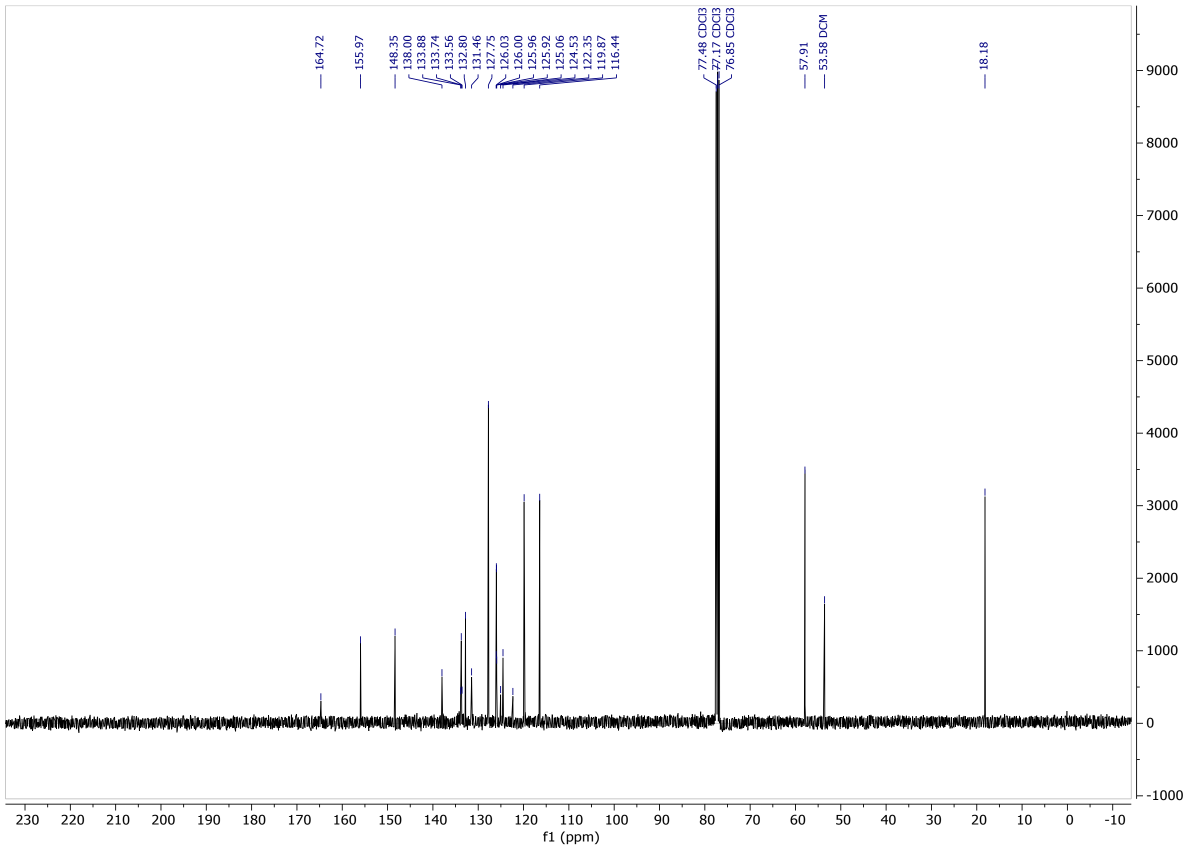

References

1. https://www.sigmaaldrich.com/NL/en/technical-documents/protocol/cell-culture-and-cell-culture-analysis/cell-counting-and-health-analysis/cell-proliferation-kit-i-mtt.
2. Van Rooden, E. J. *et al.* Mapping in vivo target interaction profiles of covalent inhibitors using chemical proteomics with label-free quantification. *Nat. Protoc.* **13**, 752–767 (2018).
3. Green, M. R. & Sambrook, J. *Molecular Cloning: A Laboratory Manual*. (2012).
